# Therapy induced senescence promotes immunogenicity in acute myeloid Leukemia through reduced EZH2 activity

**DOI:** 10.1038/s41467-026-76853-1

**Published:** 2026-09-04

**Authors:** Diego Gilioli, Simona Fusco, Teresa Tavella, Tatiana Volpari, Antonella Santoro, Martin Schönlein, Kety Giannetti, Edoardo Carsana, Nicolò Gualandi, Roberta Noberini, Chiara Brombin, Salvatore Russo, Sara Feola, Yvonne Giannoula, Rui M. M. Branca, Janne Lehtiö, Tiina M. Sikanen, Markus Haapala, Laura Passerini, Angela Andrisani, Giacomo Farina, Alessia Zangari, Anastasia Conti, Lucrezia della Volpe, Matteo Barcella, Stefano Beretta, Federico Mario Aletti, Matteo Giovanni Carrabba, Silvia Gregori, Chiara Bonini, Ivan Merelli, Fabio Ciceri, Vincenzo Cerullo, Tiziana Bonaldi, Luca Vago, Clemens A. Schmitt, Raffaella Di Micco

**Affiliations:** 1 San Raffaele Telethon Institute for Gene Therapy (SR-TIGET), IRCCS San Raffaele Scientific Institute, Milan, Italy; 2 Vita-Salute San Raffaele University, Milan, Italy; 3 University School of Advanced Studies IUSS, Pavia, Italy; 4 Research Institute of Molecular Pathology (IMP), Wien, Austria; 5 Medical Department of Hematology, Oncology and Tumor Immunology, Charité- Universitätsmedizin, Berlin, Germany; 6 Department of Experimental Oncology, IEO, European Institute of Oncology IRCCS, Milan, Italy; 7 University Center for Statistics in the Biomedical Sciences, Vita-Salute San Raffaele University, Milan, Italy; 8 Drug Research Program (DRP), ImmunoViroTherapy Lab (IVT), Division of Pharmaceutical Biosciences, Faculty of Pharmacy, University of Helsinki, Helsinki, Finland; 9 Helsinki Institute of Life Science (HiLIFE), University of Helsinki, Helsinki, Finland; 10 Translational Immunology Program (TRIMM), Faculty of Medicine Helsinki University, University of Helsinki; Haartmaninkatu 8, Helsinki, Finland; 11 Digital Precision Cancer Medicine Flagship (iCAN), University of Helsinki, Helsinki, Finland; 12 Science for Life Laboratory, Department of Oncology-Pathology, Karolinska Institutet, Solna, Sweden; 13 Drug Research Program, Division of Pharmaceutical Chemistry and Technology, Faculty of Pharmacy, University of Helsinki; Viikinkaari 5E, Helsinki, Finland; 14 Unit of Immunogenetics, Leukaemia Genomics and Immunobiology, Division of Immunology, Transplantation and Infectious Disease, IRCCS San Raffaele Scientific Institute, Milan, Italy; 15 Hematology and Bone Marrow Transplantation Unit, IRCCS San Raffaele Scientific Institute, Milan, Italy; 16 Unit of Experimental Hematology, Division of Immunology, Transplantation and Infectious Disease, IRCCS San Raffaele Scientific Institute, Milan, Italy; 17 Institute for Biomedical Technologies, National Research Council, Segrate, Italy; 18 Department of Molecular Medicine and Medical Biotechnology, Naples University Federico II; S. Pansini 5, Naples, Italy; 19 Department of Oncology and Haemato-Oncology, University of Milan, Milan, Italy; 20 Johannes Kepler University, Linz, Austria; 21 Kepler University Hospital, Department of Hematology and Oncology, Linz, Austria; 22 Max-Delbrück-Center for Molecular Medicine in the Helmholtz Association, Berlin, Germany; 23 Molecular Cancer Research Center, Charité - Universitätsmedizin, Berlin, Germany

**Keywords:** Chemotherapy, Senescence

## Abstract

Chemotherapy resistance and disease relapse are major determinants of treatment failure in acute myeloid leukemia (AML). Therapy-induced senescence (TIS) is one outcome of chemotherapy, but its immunological consequences in AML remain unclear. Here we show that ex vivo chemotherapy induces senescence in a subset of therapy-naïve AML samples. TIS is marked by elevated interferon signaling, upregulation of human leukocyte antigen (HLA) class I and II molecules, and increased presentation of leukemia- and senescence-associated peptides, conferring AML cells antigen-presenting cell-like features. These changes enhance autologous CD4^+^ and CD8^+^ T cell responses against AML, both ex vivo and in patient-derived xenograft models. TIS also restores AML sensitivity to immune checkpoint blockade therapy. Mechanistically, we identify reduced Polycomb Repressive Complex 2 (PRC2) activity as central to TIS induction and its immunogenicity. PRC2 inhibition reactivates senescence-related genes and HLA expression in non-senescent AML cells, enabling T cell activation. These findings uncover a senescence-driven immune mechanism with potential to improve therapy outcomes in AML.

## Abstract (web summary)

Acute myeloid leukemia responses to chemotherapy vary, and the effects of therapy induced senescence on anti-tumor immunity remain unclear. Here, the authors show that chemotherapy-induced senescence increases antigen presentation and T-cell recognition of leukemia cells and enhances responses to immune checkpoint blockade, while EZH2 inhibition can restore these immune features in less responsive leukemias.

## Introduction

Acute myeloid leukemia (AML) is an aggressive and heterogeneous blood cancer characterized by the abnormal expansion of myeloid progenitors in the bone marrow (BM) and peripheral blood (PB). Standard treatment has traditionally relied on chemotherapy with anthracyclines and cytarabine (AraC), often followed by allogeneic hematopoietic stem cell transplantation (allo-HSCT) for patients with specific molecular risk profiles^1^. Despite therapeutic advances, chemotherapy response remains variable, with 30-50% of patients failing to achieve complete remission after induction therapy^2^. The genetic, epigenetic and functional heterogeneity of leukemic clones contributes to the emergence of blast subpopulations associated with chemoresistance and relapse. In addition, leukemic stem cells (LSCs) may evade standard chemotherapy and contribute to disease persistence. Moreover, the BM microenvironment further promotes AML progression by creating a protective niche that shields leukemic cells from treatment^3^.

Cancer cells respond to anticancer therapies through apoptosis or by entering therapy-induced senescence (TIS), a state of prolonged growth arrest. TIS is characterized by a persistent DNA damage response (DDR), increased senescence-associated-β-galactosidase (SA-β-GAL) lysosomal enzyme activity and the activation of the senescence-associated secretory phenotype (SASP), a pro-inflammatory program involving a variety of cytokines, growth factors and metalloproteases^4,5^. Through paracrine signaling, the SASP alters stromal cells and modulates immune responses^6^. TIS can have dual effects in cancer^7,8^, acting as both a tumor-suppressive and tumor-promoting mechanism. On the one hand, senescence serves as an anti-tumorigenic barrier, inducing permanent cell cycle arrest and contributing to the initial therapeutic response^9–11^. On the other hand, if persistent, senescent cells can drive chronic inflammation and immunosuppression, fostering the emergence of therapy-resistant clones^12–14^. Recent studies in solid tumors highlight the complexity of senescence within the tumor immune microenvironment, revealing that reprogramming of senescence-associated pathways also contributes to cancer progression and immune evasion^15,16^. Notably, senescent cells also engage with the innate immune system to promote immune clearance^17–19^, upregulating activating ligands and facilitating recognition and clearance by macrophages^20^, natural killer (NK) cells^21^, dendritic cells (DCs), and invariant NK-T (iNKT)-cells^22^. Beyond innate immunity, senescence-driven immune responses can also engage the adaptive immune system, supporting cytotoxic CD8^+^ T cell responses and promoting CD4^+^ T cell activity^23–26^, thereby facilitating macrophage-mediated clearance^18,27^. In melanoma and pancreatic ductal adenocarcinoma (PDAC) models, senescent tumor cells, through the SASP, increase antigen presentation, enhancing CD8⁺ T cell–mediated immune surveillance and promoting tumor eradication^26^. Furthermore, another recent study in solid tumors demonstrated that immunization with senescent cancer cells elicits robust antitumor protection mediated by DCs and CD8⁺ T cells, surpassing that induced by immunogenic cell death (ICD)^25^.

The role of TIS in shaping AML immunogenicity and T-cell responses remains poorly defined. Although immune checkpoint blockade has shown clinical activity in several solid tumors, its effectiveness in AML remains limited.

Here, we show that ex vivo chemotherapy induces senescence in a subset of therapy-naïve AML samples and that this response is associated with increased interferon signaling, HLA class I and II expression, and presentation of leukemia- and senescence-associated peptides. These changes enhance autologous CD4^+^ and CD8^+^ T-cell responses and responses to immune checkpoint blockade ex vivo and in patient-derived xenograft models. We further identify reduced PRC2 activity as a determinant of TIS-associated immunogenicity and show that EZH2 inhibition increases HLA expression and T-cell activation in Sen Low AML. Together, these findings link TIS to adaptive immune recognition in AML and support senescence competence as a potential basis for patient stratification and therapeutic combination strategies.

## Results

### Chemotherapy triggers senescence only in a distinct subgroup of AML patients

To investigate the molecular events triggered by chemotherapy in AML, we analyzed the response to treatment in 21 newly diagnosed, chemotherapy-naïve AML patients, bearing diverse genetic and cytogenetic alterations (Supplementary Data 1). Patient-derived leukemic blasts were purified from AML samples based on CD45low/CD34 expression or, if CD34-negative, by CD45low/CD33 expression (Supplementary Fig. S1A). Purified cells were either left untreated or treated with cytarabine (AraC) and cultured for five days (120 h). Initial dose-escalation studies in primary samples showed that 400 nM AraC effectively reduced cell proliferation with only limited induction of apoptosis (Supplementary Fig. S1B, C).

**Fig. 1:**
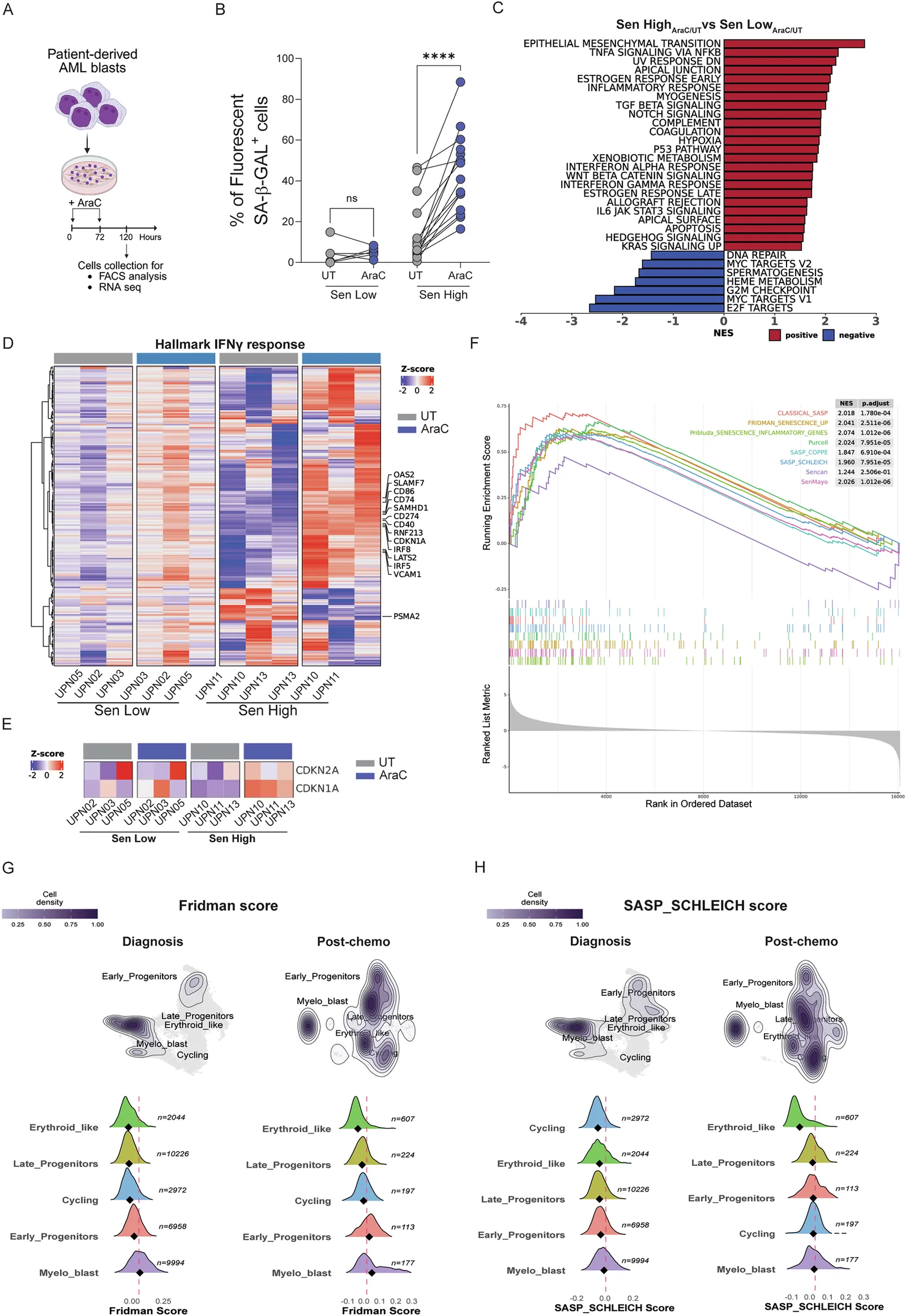
Chemotherapy triggers senescence in a distinct subgroup of AML patients. **A** Experimental design of ex vivo chemotherapy treatment in primary AML blasts. AraC was added twice (at 0 and 72 h) at a 400 nM final concentration. After 5 days of culture (120 hours), cells were collected for downstream analyses. **B** Percentages of fluorescent SA-β-GAL positive cells in untreated (UT, dots in grey) or AraC-treated (AraC, dots in light blue) samples; *n* = 6 Sen Low, *n* = 15 Sen High biologically independent patient samples. Statistical test: Two-tailed Wilcoxon matched-pairs signed-rank test. ****p < 0.0001. Source data are provided as a Source Data file. **C** Barplot showing the Normalized Enrichment Score (NES) of significant categories obtained from the GSEA. The analysis was performed over genes ranked according to the interaction-log_2_FC computed from the contrast between Sen High (AraC/UT) vs Sen Low (AraC/UT); *n* = 3 Sen Low and *n* = 3 Sen High biologically independent patient samples. See Materials and Methods for the model description. The reported categories belong to the hallmark gene set within the MSigDB v 2.7. **D** Heatmap depicting genes from IFN-γ response hallmark categories (selected genes are highlighted) induced by AraC treatment in Sen Low and Sen High patients. **E** Heatmap showing *CDKN2A* and *CDKN1A* gene expression in untreated and AraC-treated Sen Low and Sen High samples. **F** Enrichment plot showing GSEA results for published senescence signatures positively enriched in Sen High compared to Sen Low samples_._ **G**,** H** Contour (top) and ridge (bottom) plots illustrating respectively the density distribution of cells expressing senescence programs from either Fridman and SASP_SCHLEICH signatures, and the distribution of the scores across clusters, arranged in ascending order of mean expression for the *NPM1*^-mut^ scRNA-seq dataset. The red dashed line indicates the 3^rd^ quartile of the module score distribution calculated on the overall cell population. The cutoff was used to select cells with the highest average expression of the signature tested.

We first assessed proliferation (via EdU incorporation), and apoptosis (via AnnexinV staining) in paired untreated and AraC-treated samples from eight AML patients using imaging-based flow cytometry (Supplementary Fig. S1D). AraC treatment reduced proliferation across all tested samples (Supplementary Fig. S1E), with minimal impact on cell viability (Supplementary Fig. S1F). To determine whether AraC treatment was associated with the establishment of a senescence program, we quantified fluorescent senescence-associated β-galactosidase (fluorescent SA-β-GAL) activity by conventional flow cytometry across the entire cohort of AML samples following AraC treatment (Fig. 1A). Based on post-treatment SA-β-GAL positivity, samples were classified as Senescence High (Sen High) when > 10% of cells were SA-β-GAL-positive after AraC exposure and when there was a positive fold-change relative to the untreated condition. Samples that did not meet these criteria were defined as Senescence Low (Sen Low) (Fig. 1B and Supplementary Fig. S1G).

Initially, we explored transcriptional differences between Sen Low (*n* = 3) and Sen High (*n* = 3) samples at baseline, identifying 281 downregulated and 290 upregulated genes in Sen High compared to Sen Low (Supplementary Fig. S1H and Supplementary Data 2.1). Gene Set Enrichment Analysis (GSEA) revealed that Sen High samples were primarily enriched for MYC and E2F target genes, along with general metabolism-related gene categories, whereas WNT/β-catenin signaling was downregulated (Supplementary Fig. S1I). We next investigated the transcriptomic changes induced by AraC in the two groups. Principal component analysis (PCA) of bulk RNA-seq data revealed a clear separation between untreated and AraC-treated samples, as well as between Sen High and Sen Low groups, indicating distinct transcriptional responses to chemotherapy (Supplementary Fig. S1J). To identify pathways differentially regulated by chemotherapy treatment between Sen High and Sen Low groups, we applied an interaction model controlling for intra-patient variability (see Supplementary Materials; Supplementary Data 2.2). GSEA revealed that AraC-treated Sen High samples were enriched for pathways linked to epithelial-to-mesenchymal transition (EMT), inflammatory signaling (TNFα signaling via NFkB, Inflammatory response), DDR (p53 pathway, UV-response-DN), and interferon response (Interferon gamma response, Interferon alpha response). Conversely, E2F targets, MYC targets and G2M checkpoint pathways were downregulated (Fig. 1C and Supplementary Data 2.3). Consistent with the enrichment of interferon- and inflammation-related pathways, canonical interferon gamma (IFNγ) target genes (e.g., *IRF8, IRF5, OAS2)* showed increased expression in AraC-treated Sen High compared with Sen Low samples (Fig. 1D). Of note, when analyzing two well-known senescence-associated cell cycle inhibitors *CDKN1A* (p21) and *CDKN2A* (p16), we found that both were enriched in AraC-treated Sen High compared with Sen Low samples, with the former reaching statistical significance (Fig. 1E). Moreover, by probing our dataset with several published senescence-related gene lists^28–35^, we confirmed their significant enrichment in the Sen High group following AraC treatment (Fig. 1F and Supplementary Data 2.4).

We next assessed *GLB1* expression, which encodes the SA-β-GAL enzyme, in untreated and AraC-treated groups. Notably, *GLB1* levels increased in Sen High samples after treatment, while Sen Low samples showed no relevant changes (Supplementary Fig. S1K). Finally, we evaluated p21 and γH2AX protein expression in available samples from both Sen Low and Sen High cohorts. Consistent with the transcriptional data, AraC treatment induced higher p21 protein levels selectively in Sen High samples, with no evident increase in Sen Low samples. Similarly, γH2AX levels were more prominently increased in Sen High samples following treatment, supporting enhanced DDR signaling in this subgroup (Supplementary Fig. S1L, M).

To assess senescence in vivo across distinct AML subpopulations, we analyzed a published single-cell RNA-seq dataset of AML samples collected pre- and post-induction therapy^36^. In a cohort of 10 *NPM1* mutant (*NPM1*^-mut^) AML patients, the leukemic subcluster labeled as myelo-monoblasts exhibited elevated baseline TIS signatures, as determined by the Fridman and SASP gene modules. In chemotherapy-treated samples, all identified leukemic subclusters, especially myelo-monoblasts and early progenitors, showed high senescence signature scores (Fig. 1G, H). Furthermore, in a cohort of three AML patients bearing a deletion of chromosome 7 (del7 AML), the DC-like and late progenitor subclusters showed the highest baseline TIS signature scores. The myelo-monoblast cluster displayed senescence signature enrichment only at day 14 post-chemotherapy, but was no longer detectable by day 30. At that point, elevated TIS signatures were again observed in the DC-like and late progenitor clusters (Supplementary Fig. S1N, O), suggesting that senescence competence is shared across distinct leukemic subpopulations in vivo following chemotherapy.

Overall, our transcriptomic and multiparametric flow cytometry analyses indicate that chemotherapy separates AML patients based on their ability to initiate a TIS response. This response, evident in myelo-monoblasts as well as in AML progenitors, is characterized by proliferation arrest, p53-DDR pathway activation, and a robust activation of inflammatory programs.

### Senescent AML blasts upregulate antigen processing and presentation machinery

**Fig. 2:**
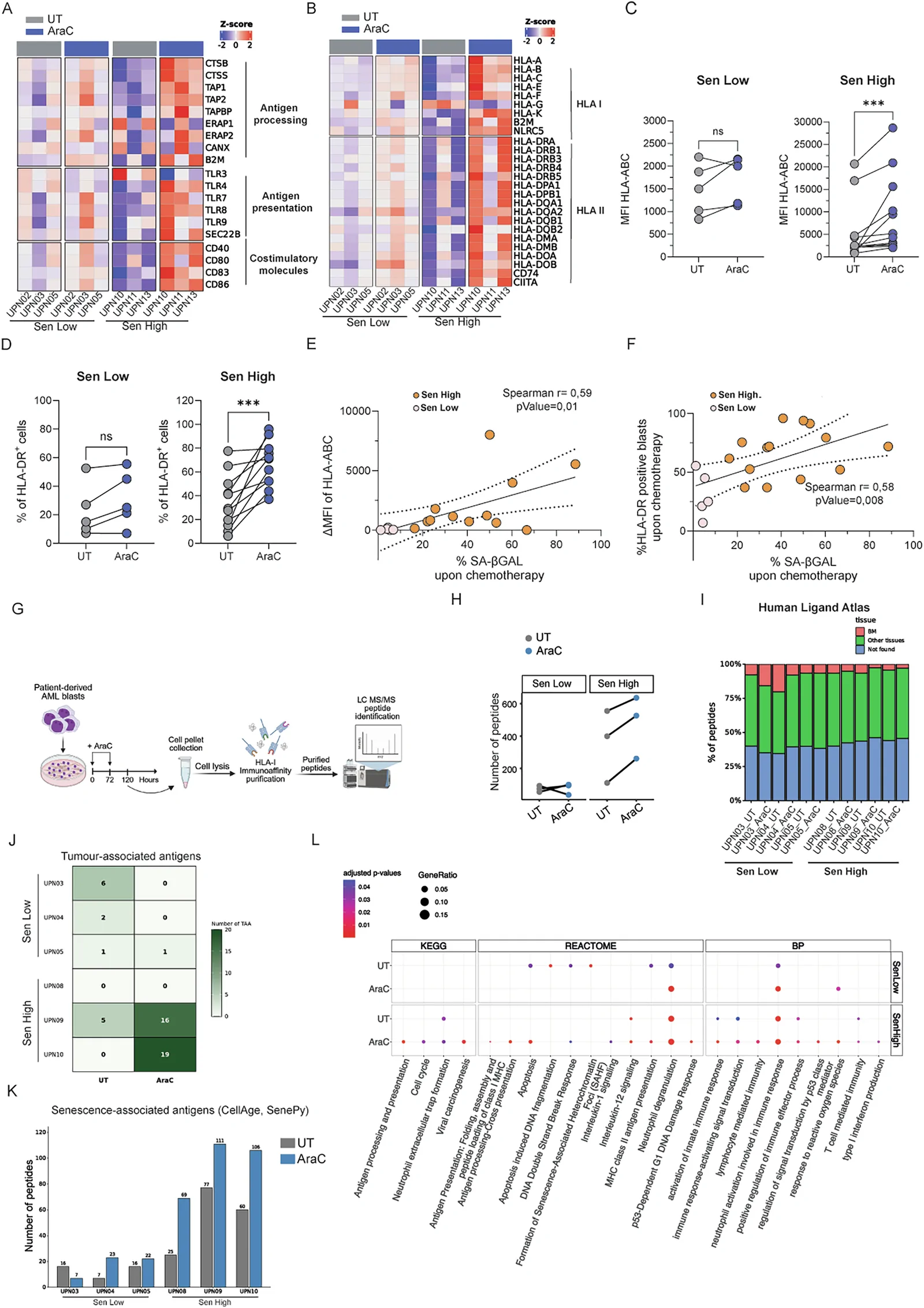
Senescent AML cells upregulate antigen processing and presentation machinery. **A**,** B** Heatmaps of genes belonging to the antigen processing, presentation, co-stimulatory molecules (**A**) or HLA class I/II genes and regulators (**B**), induced by AraC treatment in Sen Low and Sen High patients (*n* = 3 biological replicates). **C** Median Fluorescent Intensity (MFI) of HLA-ABC as detected by FACS analysis in untreated (UT) and AraC-treated primary Sen Low (left, *n* = 5) and Sen High (right, *n* = 12) biological replicates. Statistical test: One-tailed Wilcoxon matched pairs signed rank test. ***p = 0.0005. **D** Percentage of HLA-DR positive cells as detected by FACS analysis in untreated (UT) and AraC treated Sen Low (left, *n* = 5) and Sen High (right, *n* = 12) biological replicates. Statistical test: One-tailed Wilcoxon matched pairs signed rank test. ***p = 0.0002. **E** Correlation between SA-β-GAL-positive cells upon AraC treatment and HLA-ABC-ΔMFI (ΔMFI = MFI_AraC_ − MFI_UT_) signal. Negative ΔMFI values = 0. Each dot represents a biological replicate (Sen High, orange, *n* = 12; Sen Low, light orange, *n* = 5). Statistical analysis: two-sided Spearman rank correlation (*n* = 17; ${r}_{s}\,$= 0.5921, 95% CI [0.1407, 0.8397], exact *p* = 0.0140). **F** Correlation between SA-β-GAL positive cells upon AraC treatment and HLA-DR (class II) positive cells; each dot represents a biological replicate (Sen High, orange, *n* = 14; Sen Low, light orange, *n* = 5). Statistical analysis: two-sided Spearman rank correlation (*n* = 19; ${r}_{s}\,$= 0.5838, 95% CI [0.1624, 0.8252], approximate *p* = 0.0087). **G** Experimental design of immunopeptidomic analysis in AML samples (*n* = 3 biological replicates each cohort). **H** Number of peptides retrieved from untreated (UT, grey) and AraC-treated (light blue) Sen Low and Sen High samples after the filtering steps described in Methods. **I** Correspondence between peptides retrieved in each sample and the Human Ligand Atlas, classified as HLA class I/ II- or dual-binding peptides. **J** Number of AML-associated tumor antigens detected in each sample. **K** Barplot reporting the number of senescence-associated antigens from CellAge and Senepy databases. **L** Manually curated enrichment results for gene sets associated with peptides identified by immunopeptidomics in each condition (FDR < 0.05). Source data are provided as a Source Data file.

Considering the enrichment in transcripts belonging to inflammation and IFNγ production, we then investigated whether TIS establishment may enhance the immunogenicity of AML blasts. Since IFNγ is known to upregulate components of the antigen processing and presentation machinery, we assessed the expression of relevant genes involved in this pathway in Sen High and Sen Low AML samples following AraC treatment. Of note, transcripts involved in peptides cleavage and transport (i.e., cathepsin B, *CTSB*), antigen recognition (i.e., *TLR4)*, and costimulatory molecules (i.e., *CD40, CD86)* were significantly upregulated in Sen High compared to Sen Low samples AraC treated (Fig. 2A). Along this line, expression of human leukocyte antigen (HLA) I and II genes, among which *HLA-B, HLA-C, HLA-F, HLA-K, HLA-DMA, HLA-DMB, HLA-DRB1/3/4, HLA-DPA1, HLA-DPB1*, were also upregulated in the Sen High compared to the Sen Low samples upon treatment (Fig. 2B and Supplementary Data 2.5). Importantly, flow cytometry analysis further confirmed the increased surface expression of HLA class I (HLA-ABC) and HLA class II (HLA-DR) in Sen High blasts post-treatment (Fig. 2C, D and Supplementary Fig. S2A), and this upregulation was positively correlated with fluorescent SA-β-GAL activity (Fig. 2E, F).

To extend these observations at single-cell resolution, we analyzed a previously published single-cell RNA-seq dataset of ex vivo chemotherapy-treated AML samples^13^. HLA class I and II genes were significantly enriched in clusters with high Fridman senescence scores (clusters 0, 3, 4 and 6) (Supplementary Fig. S2B and Supplementary Data 3). To determine whether the increase in TIS signatures observed in a single-cell dataset of in vivo chemotherapy-treated AML patients^36^ was associated with upregulation of HLA molecules, we performed correlation analyses between HLA class I (HLA-ABC) and class II (HLA-DR) expression scores and the Fridman senescence score. In *NPM1*^-mut^ patients, the myelo-monoblast cluster exhibited the strongest correlation with both HLA-ABC and HLA-DR expression at baseline, which was further enhanced following chemotherapy, despite a reduction in blast cell numbers. Similarly, the late progenitor population showed a modest post-chemotherapy correlation between HLA expression and the TIS signature, whereas early progenitors displayed no significant association (Supplementary Fig. S2C). In the del7 cohort, all the leukemic subclusters showed a positive correlation with HLA-ABC expression post-chemotherapy treatment, while HLA-DR expression positively correlated with the senescence signature score post-treatment in myelo-monoblast and DC-like subclusters (Supplementary Fig. S2D). To define the cellular identity of these senescence-enriched AML populations, we performed reference mapping of the AraC-treated clusters from the Duy et al. dataset using multiple independent scRNA-seq atlases, including healthy BM and AML references^36–39^. Across datasets, the clusters showing the strongest enrichment for senescence-associated programs and HLA class I/II expression (cluster 0, 4, 6) were consistently assigned to a monocyte state (Supplementary Fig. S2E). In line with this, these clusters also showed higher expression of canonical monocytic markers, including *CD14, CD36, CD68, S100A8, S100A9* and *MNDA* (Supplementary Fig. S2F). Together, these analyses support the idea that senescence preferentially arises within a monocyte-primed myeloid compartment and is associated with enhanced antigen-presentation features.

To determine whether the overall increase in antigen presentation machinery was accompanied by qualitative changes in the presented repertoire, we performed HLA class I immunopeptidomics on paired untreated and AraC-treated primary AML samples (Fig. 2G). Using immunoaffinity purification followed by LC-MS/MS, we found that Sen High samples displayed a broader peptide repertoire than Sen Low samples at baseline, which was further expanded upon chemotherapy, whereas Sen Low samples remained comparatively poor in peptide yield across conditions (Fig. 2H). To explore the disease-associated nature of the identified peptides, we compared them to a reference dataset^40^ of peptides eluted from healthy tissues, including healthy BM samples. Strikingly, most peptides identified in AML samples were not detected in the healthy tissue atlas (Fig. 2I), suggesting that the AML immunopeptidome is enriched for disease-associated peptide species.

We next interrogated peptides mapping to known tumor-associated antigens (TAAs)^41^ across Sen Low and Sen High samples and compared untreated (UT) and AraC-treated conditions (Fig. 2J). This analysis revealed a distinct group-level pattern: Sen Low samples displayed very few TAA-associated peptides and no evident treatment-dependent increase, whereas Sen High samples showed an AraC-associated increase in TAA presentation. We then focused on senescence-associated antigens by matching peptide source genes to curated senescence-related gene lists, including CellAge^42^ and SenePy^43^. AraC treatment increased the number of senescence-associated peptides in all three Sen High samples, with a stronger and more consistent magnitude than that observed in Sen Low samples. Although a modest increase was detectable in a subset of Sen Low cases, this effect was less pronounced and less reproducible than in Sen High samples (Fig. 2K and Supplementary Data 4).

Finally, we performed functional enrichment analysis on genes encoding the source proteins of the identified peptides. Enriched categories revealed a significant over-representation of pathways related to p53-mediated DDR, interleukin signaling, innate immune system activation, T cell-mediated immunity and antigen processing and presentation in AraC-treated Sen High samples. Conversely, peptides from Sen Low samples were preferentially enriched in pathways related to apoptosis, DDR, reactive oxygen species (ROS) and Formation of Senescence-Associated Heterochromatin Foci (SAHF) (Fig. 2L). Consistent with this enhanced immunogenicity, bulk RNA-seq analysis revealed that Sen High samples also upregulated transcriptional programs associated with conventional dendritic cells (DC) type 1 and type 2 (cDC1, cDC2) (Supplementary Fig. S2G and Supplementary Data 5), which are known to initiate T cell responses via antigen cross-presentation and HLA- restricted presentation, respectively^44^.

Taken together, these findings indicate that chemotherapy-induced senescence AML states are coupled to enhanced antigen-presentation programs and diversification of the HLA-bound peptide repertoire, potentially strengthening leukemia-immune interactions with the adaptive immune system.

### T-cell activation in the presence of senescent AML cells occurs in an HLA-dependent manner

To investigate the immunological consequences of HLA upregulation during TIS, we first performed co-culture experiments using the AML monocytic cell line THP-1, rendered senescent by treatment with AraC, and CD3^+^ T cells from healthy donors. AraC treatment at the dose selected from dose-escalation experiments (200 nM, Supplementary Fig. S3A–E) increased both fluorescent SA-β-GAL signal (Supplementary Fig. S3F) and HLA class I and II surface expression in THP-1 cells (Supplementary Fig. S3G, H).

**Fig. 3:**
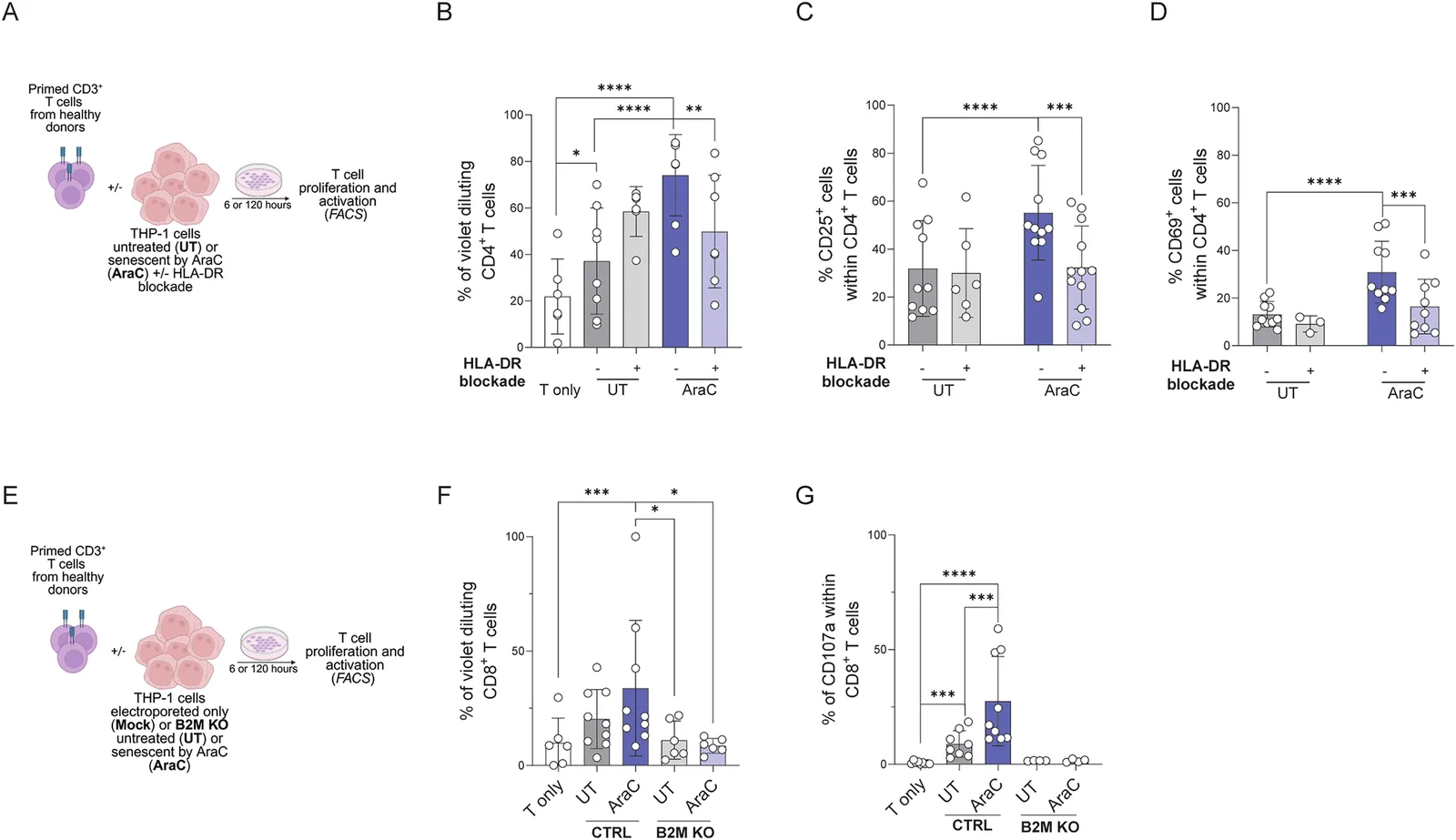
T-cell activation in the presence of senescent AML cells occurs in an HLA-dependent manner. **A** Experimental design of the Mixed Lymphocyte Culture assay. **B** Proliferating CD4^+^ T cells (healthy donors) measured as % of violet diluting T cells co-cultured with untreated (UT, grey, *n* = 6) or AraC-treated cells (AraC, light blue) in the presence or not of HLA-DR blockade. (T alone, *n* = 6; UT, *n* = 6; AraC, *n* = 7; UT + HLA-DR blockade, *n* = 8; AraC + HLA-DR blockade, *n* = 8). Data shown as mean ± SD. Statistical test: LME model raw outcome. ****p < 0.0001; **p < 0.01; *p < 0.01. **C**, **D** Percentage of CD25^+^ (**C**) cells or CD69^+^ (**D**) within CD4^+^ T cells detected by FACS analysis after co-culture with different target cells. For each condition, *n* refers to panel **C** and panel **D**, respectively: UT, *n* = 10/10; AraC, *n* = 11/10; UT + HLA-DR blockade, *n* = 6/3; AraC + HLA-DR blockade, *n* = 12/9. Data shown as mean ± SD. Statistical test: LME model (**C**) raw or (**D**) cubic root-transformed outcome. ****p < 0.0001; ***p < 0.001; **p < 0.01; *p < 0.05. **E** Experimental design of the Mixed Lymphocyte Culture assay with B2M KO THP-1 cells. **F** Proliferating CD8^+^ T cells measured as % of violet diluting T cells co-cultured with different targets as indicated in panel **E**. (T alone, *n* = 6; UT, *n* = 9; AraC, *n* = 9; UT B2M KO, *n* = 6; AraC B2M KO, *n* = 6). Data shown as mean ± SD. Statistical test: LME model square-root transformed outcome. ****p < 0.0001; **p < 0.01; *p < 0.05. **G** Percentage of degranulating CD8^+^ T cells (measured by flow cytometry as CD107a^+^ cells, 6 h post-co culture) alone or co-cultured with targets as indicated in panel (**E**). (T alone, *n* = 9; UT, *n* = 8; AraC, *n* = 9; UT B2M KO, *n* = 4; AraC B2M KO, *n* = 4). Data shown as mean ± SD. Statistical test: LME model ordernorm-transformed outcome. ****p < 0.0001; ***p < 0.001. Source data are provided as a Source Data file.

To determine the functional role of HLA class II, we employed an HLA-DR blocking antibody in untreated or AraC-treated THP-1 cells (Fig. 3A). T cells were first primed through a one-week co-colture leukemic cells bearing enhanced HLA class II and class I expression following IFN-γ-pretreatment^45^. T-cell proliferation (via CellTrace Violet dye dilution) and activation (measured by CD69 and CD25 expression levels) were assessed to monitor their functional engagement. While untreated leukemic cells elicited minimal CD4^+^ T-cell responses, senescent cells induced robust proliferation and activation, which were significantly reduced upon HLA-DR blockade treatment (Fig. 3B–D and Supplementary Fig. S3I, J). To investigate the role of HLA class I, we genetically inactivated β2-microglobulin (B2M) via CRISPR-Cas9 technology (Supplementary Fig. S3K). Wild-type or B2M-KO cells, either left untreated or AraC-treated were co-cultured with CD3^+^ T cells (Fig. 3E). While AraC-treated wild-type cells promoted strong CD8^+^ T-cell proliferation, this response was abolished in B2M-deficient cells (Fig. 3F). Similarly, CD107a-mediated degranulation was reduced in CD8^+^ T cells when co-cultured with B2M knockout cells, regardless of AraC treatment (Fig. 3G and Supplementary Fig. S3L).

Collectively, these results show that, upon TIS establishment in this AML model, efficient CD8^+^ and CD4^+^ T cell activation relies on HLA class I- and HLA class II-mediated antigen presentation, respectively.

### TIS enhances AML immunogenicity and synergizes with immune checkpoint blockade both ex vivo and in vivo

**Fig. 4:**
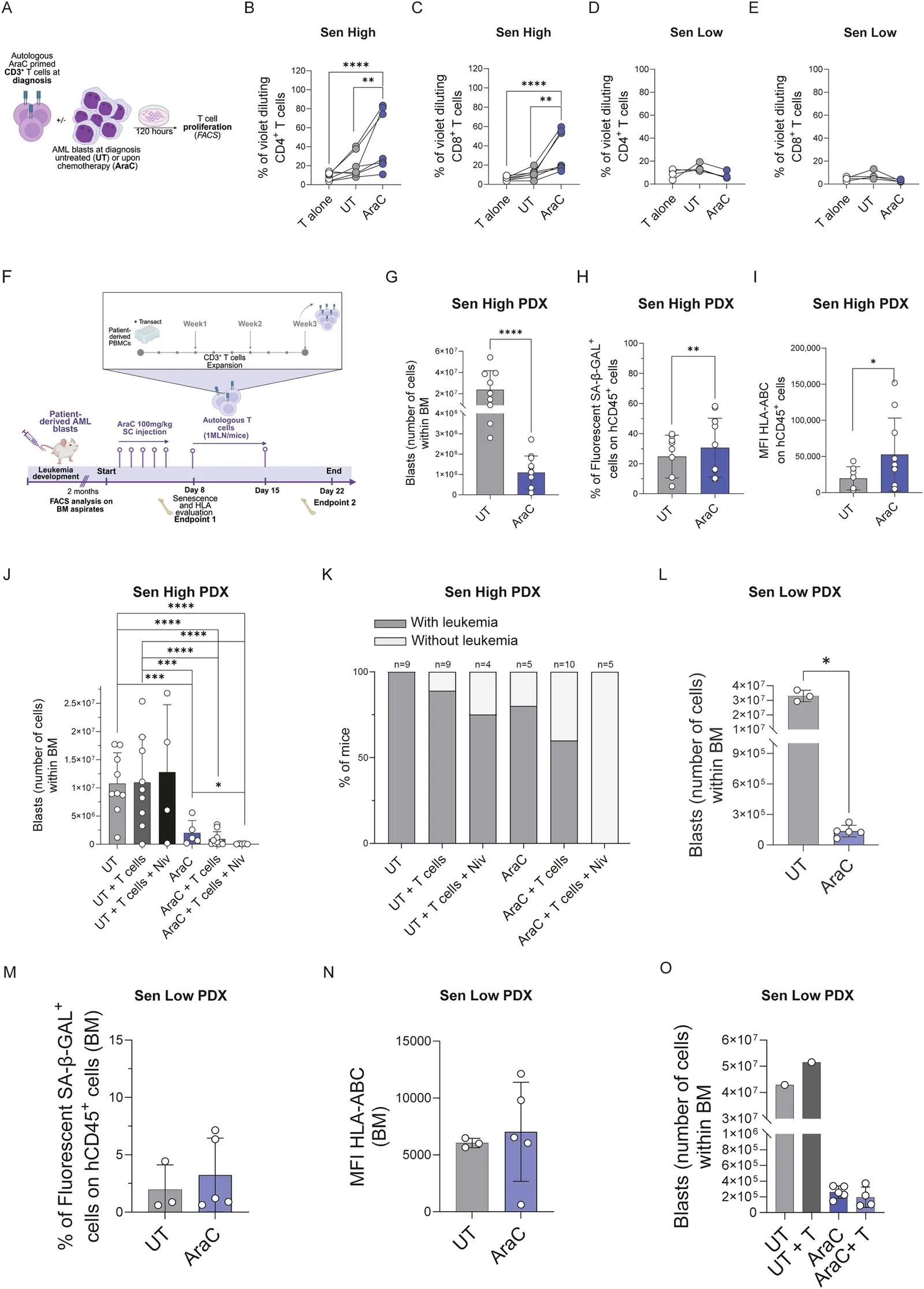
TIS enhances AML immunogenicity and synergizes with ICBs ex vivo and in vivo. **A** MLR assay design. **B**, **C** Proliferating CD4^+^ (**B**) or CD8^+^ (**C**) T cells, measured as violet diluting cells upon co-culture with untreated (UT, grey) or AraC-treated (light blue) Sen High samples (*n* = 7 biological replicates). Statistical test: LME model (**B**) cubicroot-transformed or (**C**) log-transformed outcome. **D**, **E** Proliferating CD4^+^ (**D**) or CD8^+^ (**E**) T cells measured as violet diluting cells upon co-culture with UT (grey) or AraC (light blue) Sen Low samples (*n* = 4 biological replicates). **F** Sen High PDX experimental design. **G** Number of blasts in UT (grey, *n* = 9) or AraC (light blue, *n* = 12) mice at endpoint 1. **H** SA-β-GAL positive cells in UT (grey; *n* = 9) or AraC (light blue; *n* = 12) mice at TP1. **I** HLA-ABC MFI on hCD45^+^ cells in UT (grey; *n* = 7) and AraC (light blue; *n* = 10) mice at TP1. **J** Number of blasts at endpoint 2 in UT (grey; *n* = 9), UT + T cell (dark grey; *n* = 9), UT + T cells + Nivolumab (black; *n* = 4), AraC (light blue; *n* = 5), AraC + T cell (blue; *n* = 10), and AraC + T cell + Nivolumab (dark blue; *n* = 5) mice. **K** Mice with (grey) or without leukemia (light grey) at TP2. **L** Number of blasts in UT (grey, *n* = 3) or AraC (light blue, *n* = 5) mice from a Sen Low sample at TP1. Statistical test: One-tailed Mann-Whitney test. *p < 0.0179. **M** SA-β-GAL positive cells in UT (grey, *n* = 3) or AraC (light blue, *n* = 5) mice at TP1. **N** HLA-ABC MFI on hCD45^+^ cells in UT (grey, *n* = 5) and AraC (light blue, *n* = 5) mice at TP1. **O** Number of blasts at TP2 in UT (grey, *n* = 1), UT + Tcells (dark grey, *n* = 1), AraC (blue, *n* = 5) and AraC + T cells (light blue, *n* = 4) mice. Unless otherwise specified: data shown as Mean ± SD, statistical test LME model log transformed outcome, exact *p-*values in Supplementary Table 1. Source data are provided as a Source Data file.

To evaluate TIS-induced immunogenicity in primary AML, we performed co-culture assays using patient-derived autologous CD3^+^ T cells and AML blasts either untreated (UT) or treated with AraC (Fig. 4A). After five days of co-culture, we measured T cells proliferation. In the Sen High group, AraC-treated blasts led to a significant increase in proliferation of both CD4^+^ and CD8^+^ T cells (Fig. 4B, C). In contrast, T cells from Sen Low patients showed minimal proliferation regardless of AraC treatment (Fig. 4D, E). To determine whether this differential response could be attributed to intrinsic differences in T cell phenotypes, we analyzed exhaustion markers (PD-1, TIM-3, and LAG-3) and the distribution of T cells subsets from available Sen Low and Sen High AML samples at diagnosis. Notably, we found no significant differences in the expression of these markers between the Sen High and Sen Low groups (Supplementary Fig. S4A). Similarly, a comprehensive immunophenotypic analysis of T cell subsets, including markers for the identification of naive, stem cell memory (SCM), central memory (CM), effector memory (EM) and terminal effector (EFF) cells, did not reveal any major difference between Sen High and Sen Low patients (Supplementary Fig. S4B).

We next investigated whether T cell activity in response to TIS could be further enhanced by immune checkpoint blockade (ICB) treatment. We performed a co-culture assay using autologous CD3^+^ T cells and matched AML blasts from both Sen High and Sen Low samples, treated with ipilimumab (anti-CTLA-4), nivolumab (anti-PD-1), or durvalumab (anti-PD-L1) (Supplementary Fig. S4C). In Sen Low samples, CD8⁺ and CD4⁺ T cell proliferation remained low across all conditions, and ICB treatment failed to enhance T cell response, regardless of AraC exposure (Supplementary Fig. S4D, E). Conversely, in Sen High samples, AraC-treated blasts further enhanced CD8⁺ T-cell proliferation in the presence of ICBs, while untreated blasts remained unresponsive (Supplementary Fig. S4F). Instead, CD4⁺ T cells did not display further enhancement with ICB in both conditions (Supplementary Fig. S4G). These functional results are consistent with our bulk RNA-seq data, which revealed higher AraC-induced upregulation of immune-inhibitory ligands, such as PD-L1 (*CD274*), in Sen High compared to Sen Low samples (Supplementary Fig. S4H). Altogether, these findings indicate that TIS enhances adaptive T cell responses against AML and that this immunogenic reprogramming can be exploited to boost T cell activity, particularly when combined with ICBs.

We next extended these observations in vivo using a humanized NOD-SCID-IL2Rg^-^/^-^ (NSG) model transplanted with AML blasts from a Sen High patient of our cohort (Fig. 4F). Following leukemia engraftment, a group of mice was treated with AraC for five consecutive days and euthanized at day 8. At the first experimental endpoint, AraC treatment induced a marked reduction in leukemia burden early after chemotherapy treatment (Fig. 4G) and was accompanied by increased fluorescent SA-β-GAL accumulation (Fig. 4H) and HLA-I upregulation in residual AML blasts (Fig. 4I).

To evaluate T cell-mediated leukemia control, additional mice received two infusions of autologous patient-derived CD3^+^ T cells.

Before infusion, T cells were expanded ex vivo using a previously optimized clinical protocol^46^, resulting in an approximately ten-fold increase in CD3^+^ cell number while preserving CD4^+^/CD8^+^ ratios (Supplementary Fig. S4I) and overall T cell subsets composition (Supplementary Fig. S4J). At the second experimental endpoint, we first quantified the frequency of T cells within the human graft in the BM and found detectable T-cell engraftment/infiltration in the BM, with higher T-cell frequencies in the chemotherapy group (Supplementary Fig. S4K). We then also assessed the expression of the activation markers CD25 and CD69 on T cells. Notably, while CD25 expression showed no appreciable changes, CD69, a well-established activation marker, was higher in the AraC + T group compared with UT + T one (Supplementary Fig. S4L). We next evaluated leukemia burden in mice receiving infusions of autologous patient-derived CD3^+^ T cells, either alone or in combination with the anti PD-1 mAb, nivolumab. AraC-treated mice displayed reduced leukemic burden in the BM, which was further decreased by autologous T cell infusion and even more evident upon nivolumab treatment. Instead, infused T cells had no appreciable effect in chemotherapy-naïve mice, irrespectively of nivolumab treatment (Fig. 4J). Consistently, when mice were stratified according to the presence or absence of detectable leukemia at endpoint, the AraC + T cells + nivolumab group showed the highest proportion of leukemia-free (mice with < 1% of human CD45^+^ cells) animals, with all evaluable mice being leukemia-free at the end of the experiment (Fig. 4K).

Finally, we performed an experiment in a Sen Low PDX using the same AraC-based regimen and autologous T cell transfer schedule applied to the Sen High model (Fig. 4F). Also in this setting, autologous T cells were expanded ex vivo before infusion and did not report any difference in terms of CD4^+^/CD8^+^ ratios (Supplementary Fig S4M). We observed a marked AraC-induced reduction in leukemia burden (Fig. 4L), consistent with its strong cytotoxic effect. However, unlike in the Sen High model, residual leukemic cells did not show a clear accompanying increase in SA-β-GAL activity (Fig. 4M) or in HLA class I expression (Fig. 4N). Accordingly, subsequent administration of autologous T cells did not confer an additional measurable reduction in leukemia burden at endpoint (Fig. 4O).

Together, these in vivo findings indicate that Sen High and Sen Low AML are comparable in terms of initial sensitivity to chemotherapy but differ in their capacity to mount a TIS-associated immunogenic response that can be functionally leveraged by autologous T cells.

### EZH2 inhibition enhances immunogenicity in Senescence Low samples

To investigate the mechanisms underlying differences in senescence competence and immunogenicity between Sen High and Sen Low AML patients, we first compared genetic alterations (Supplementary Fig. S5A) and karyotypic profiles across the two groups (Supplementary Data 1). Mutational and cytogenetic features did not reveal an obvious segregation according to senescence status, although the cohort size does not allow formal exclusion of genotype-associated effects (Supplementary Fig. S5A). We next asked whether baseline cellular composition might account for these differences. Deconvolution of our bulk RNA-seq data using a reference scRNA-seq dataset from *NPM1*^-mut^ AML^36^ revealed a comparable proportion of TIS-susceptible myeloblast-like populations in the two groups, with only a small fraction of early and late progenitor-like cells detected in Sen Low samples (Supplementary Fig. S5B). This difference appeared insufficient to account for the markedly distinct transcriptional and immune responses observed after chemotherapy.

**Fig. 5:**
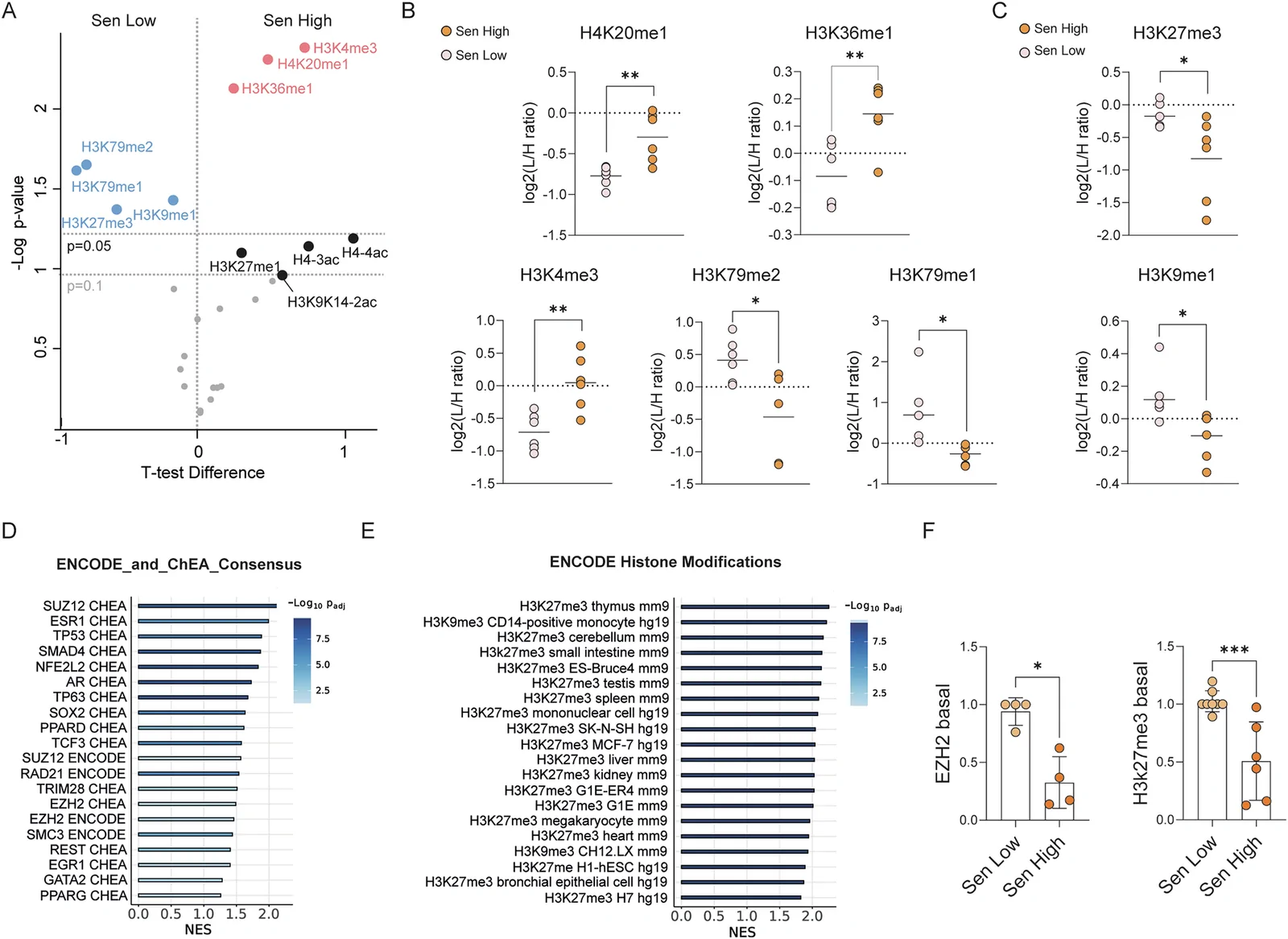
Increased H3K27me3 and EZH2 basal levels in Sen Low samples. **A** Volcano plot showing histone post-translational modifications (PTMs) quantified by mass spectrometry analysis. Histone PTMs with *p*-value between 0.1 and 0.05 are highlighted in black; histone PTMs with *p*-value < 0.05 are highlighted in blue for the Sen Low group and in red for the Sen High group. Data were obtained from *n* = 3 biologically independent AML patient samples per group, each analyzed in two independent experiments performed on separate days, resulting in *n* = 6 measurements per group. Statistical test performed: two-tailed T-test. **B**, **C** Dot plot showing log2 (L/H ratios, where L: samples, H: internal standard) for histone PTMs with *p-* value < 0.05 associated with (**B**) transcriptional activation (H4K20me1, H3K36me1, H3K4me3, H3K79me2, H3K79me1) or (**C**) repression (H3K27me3, H3K9me1) identified in Sen High patients (orange dots) and Sen Low patients (light orange dots). Data were obtained from *n* = 3 biologically independent AML patient samples per group, each analyzed in two independent experiments performed on separate days, resulting in *n* = 6 measurements per group. Values are shown as mean ± SD. Statistical test performed: two-tailed T-test; * *p* < 0.05; ** *p* < *0.01*. Source data are provided as a Source Data file. **D**, **E** Barplot showing the NES of significant categories from the GSEA over the ENCODE categories (ENCODE_and_ChEA_Consensus_TFs_from_ChIP-X and ENCODE Histone Modifications. **F** Relative quantification of H3K27me3 and EZH2 protein levels obtained by western blot analysis in untreated Sen Low (*n* = 8) and Sen High (*n* = 6) AML samples. Data are shown as values from individual patient samples, with mean ± SD. Statistical test: One-tailed Mann-Whitney. ***p = 0.0007; *p = 0.0143.

We therefore hypothesized that baseline epigenetic dysregulation, and in particular the distribution of histone post-translational modifications (PTMs), might underline the divergent responses of the two groups to AraC treatment. To test this hypothesis, we employed super-SILAC mass spectrometry to perform a quantitative analysis of histone modifications in untreated AML patients stratified by senescence competence. Strikingly, Sen Low patients exhibited a global enrichment in histone modifications associated with transcriptional repression and closed chromatin state. In contrast, Sen High patients showed significantly elevated levels of histone marks linked to open chromatin and increased transcriptional activity (Fig. 5A and Supplementary Fig. S5C). More specifically, Sen High samples had significantly higher basal levels of H3K4me3, H4K20me1, and H3K36me1, histone modifications generally associated with active gene expression (Fig. 5B). Furthermore, acetylated peptides on histone H3 (H3K9K14-2ac) and H4 (H4-3ac and H4-4ac) also showed a modest increase in Sen High patients, consistent with a more open chromatin state. Conversely, Sen Low patients showed elevated levels of the repressive histone mark H3K27me3 and of the precursor of heterochromatin formation H3K9me1 (Fig. 5C). Instead, H3K79me1 and H3K79me2 were higher in Sen Low patients compared to Sen High. Although these marks are generally linked to transcriptional activation, their distribution in this context may reflect non-canonical or cell type-specific regulatory functions.

To extend these observations, we interrogated our bulk RNA-seq dataset for epigenetically regulated gene sets. GSEA analysis from the interaction model revealed that in Sen High *vs* Sen Low samples upon AraC treatment, SUZ12 and EZH2 target gene sets were enriched, together with multiple H3K27me3-associated gene sets (Fig. 5D, E). As SUZ12 and EZH2 are core Polycomb Repressive Complex 2 (PRC2) components and H3K27me3 represents the canonical PRC2-dependent repressive mark, these findings suggest transcriptional de-repression of PRC2-regulated genes in Sen High samples. In line with this, untreated Sen Low samples displayed higher basal EZH2 protein levels and increased H3K27me3 compared to Sen High samples (Fig. 5F), together with a trend for increased expression of several PRC2 core genes (Supplementary Fig. S5D). Overall, these data suggest that Sen Low samples are characterized by a stronger PRC2-associated repressive state at baseline.

**Fig. 6:**
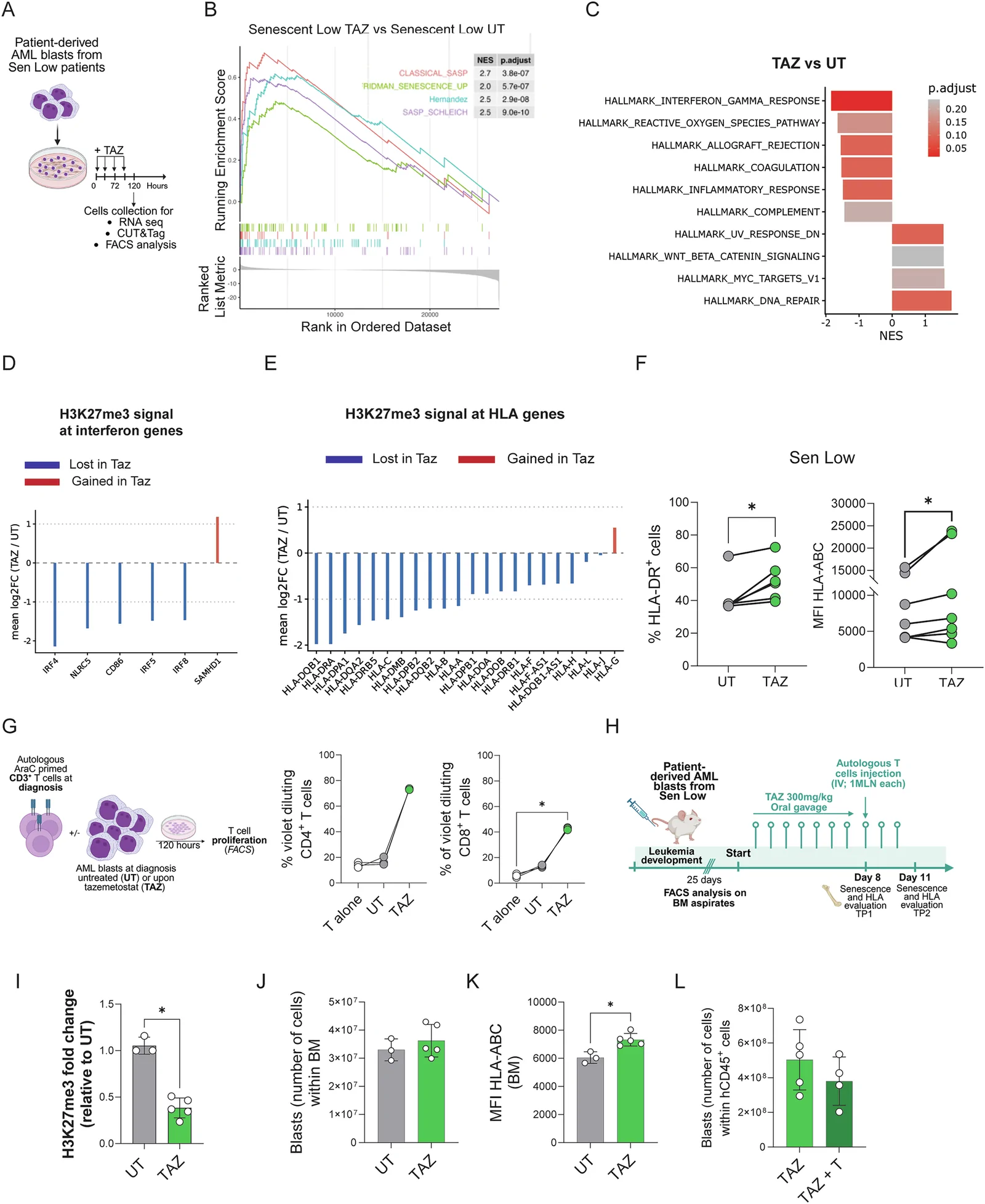
EZH2 inhibition enhances immunogenicity in Senescence Low samples. **A** Experimental design of ex vivo Tazemetostat (TAZ; 10 μM) treatment in Sen Low primary AML samples. **B** Enrichment plot showing GSEA for senescence signatures positively enriched in Sen Low patients upon TAZ treatment compared to untreated (*n* = 3 for each condition). **C** Divergent barplot reporting the NES score from GSEA of TAZ-treated vs untreated AML blasts (*n* = 1 biological sample) from CUT&TAG libraries targeting H3K27me3 modification. **D**, **E** Barplot showing the H3K27me3 CUT&TAG log2FC signal from the TAZ experiment, showing selected (**D**) interferon genes and (**E**) HLA genes. **F** Left, HLA-DR positive cells as detected by FACS analysis in untreated (UT, grey) or TAZ-treated (TAZ, green) blasts from Sen Low patients (*n* = 6 biological replicates). Right, HLA-ABC MFI in UT (grey) or TAZ (green) Sen Low samples (*n* = 7 biological replicates). Statistical test: One-tailed Wilcoxon matched pairs signed rank test. *p < 0.05. **G** Left, MLR experimental design; right, proliferating CD4^*+*^ and CD8^*+*^ autologous T cells measured as violet diluting cells upon co-culture with UT (grey) or TAZ (green) Sen Low AML samples (*n* = 3 biological replicates). Statistical analysis: exact Friedman test for CD4⁺ T cells [*Q*^2^ = 4.667, Kendall’s *W* = 0.778] and CD8⁺ T cells [*Q*^2^ = 6.000, Kendall’s *W* = 1.000], followed by Dunn’s multiple-comparisons test for CD8⁺ T cells; *p = 0.0278. **H** Sen Low PDX experimental design. **I**, Quantification of H3K27me3 signal (normalized to H3 signal) in TAZ-treated mice (*n* = 5) relative to untreated samples (*n* = 3). Statistical test: One-tailed Mann-Whitney test. *p = 0.0179. **J** Number of blasts in UT (grey, *n* = 3) or TAZ (green, *n* = 5) mice one week after treatment started. **K** HLA-ABC MFI on hCD45^+^ cells in UT (grey, *n* = 3) and TAZ (green, *n* = 5) mice. Statistical test: One-tailed Mann-Whitney test. *p = 0.0179. **L** Number of blasts at the endpoint in TAZ (green, *n* = 5) and TAZ + T cell (dark green, *n* = 4) mice. Data shown as Mean ± SD. Source data are provided as a Source Data file.

Based on these observations, we asked whether pharmacological inhibition of EZH2, the catalytic subunit of PRC2, could rescue the Sen Low samples’ immunogenicity. We therefore treated ex vivo primary Sen Low AML samples with the clinically approved EZH2 inhibitor Tazemetostat (TAZ)^47^ (Fig. 6A). RNA-seq analysis revealed induction of senescence and inflammation-related transcriptional programs following EZH2 inhibition^29,33,34,48^ (Fig. 6B). However, TAZ treatment did not increase SA-β-GAL activity in Sen Low samples (Supplementary Fig. S6A).

To directly assess whether EZH2 inhibition relieves PRC2-mediated repression at loci relevant to this phenotype, we performed low-input H3K27me3 Cut&Tag on a Sen Low sample, either untreated or treated with TAZ. We first performed an unbiased pathway enrichment analysis on loci showing reduced H3K27me3 upon TAZ treatment. Notably, the IFNγ response emerged as the top-ranked and most significantly enriched signature in this analysis (Fig. 6C). Based on this result, we focused on immune-related regions and found that TAZ treatment was associated with reduced H3K27me3 signal at interferon-related genes (Fig. 6D and Supplementary Fig. S6B), as well as across multiple HLA loci (Fig. 6E), providing chromatin-level evidence that PRC2 inhibition modulates immune-relevant loci in Sen Low AML blasts. Consistent with these data, flow cytometric analysis showed significant upregulation of both HLA class II and class I expression across treated Sen Low samples upon TAZ exposure (Fig. 6F). By contrast, when tested in Sen High samples, EZH2 inhibition did not induce additional HLA class II upregulation and only modestly affected HLA class I expression (Supplementary Fig. S6C).

Functionally, the co-culture of TAZ-treated AML blasts from Sen Low samples resulted in enhanced proliferation of both CD4⁺ and CD8⁺ T cells (Fig. 6G). Collectively, these data identify baseline PRC2-associated repression as a distinguishing feature of Sen Low AML and indicate that EZH2 inhibition can partially reprogram this state toward a more immune-visible phenotype.

To evaluate the in vivo impact of EZH2 inhibition, we transplanted NSG mice with patient-derived AML blasts from a Sen Low sample. After confirming leukemia engraftment by BM aspirates, mice were treated with TAZ (300 mg/kg, oral gavage, daily for 7 days) and organs were collected at endpoint for downstream analyses (Fig. 6H). We first verified in vivo successful target inhibition by western blot analysis, which showed a clear reduction in global H3K27me3 levels in leukemic cells from TAZ-treated animals compared to controls (Fig. 6I, Supplementary Fig. S6D). At the endpoint, TAZ alone did not markedly reduce bulk leukemia burden (Fig. 6J) but induced a significant increase in HLA class I expression on the human graft (Fig. 6K), consistent with immune-priming rewiring in vivo. Finally, to test whether the TAZ-induced immunogenic rewiring could translate into enhanced leukemia control by T cells, we administered patient-matched autologous T cells, previously expanded ex vivo and used also in the previous Sen Low PDX experiment (Fig. 6H). At the endpoint, we observed a trend toward reduced leukemia burden in the TAZ plus autologous T-cell condition compared with TAZ alone (Fig. 6L).

These data suggest that EZH2 inhibition may increase the susceptibility of Sen Low AML to immune-mediated control.

## Discussion

In this study, we investigated the interactions between senescent cells and the adaptive immune system in AML. By modeling chemotherapy response ex vivo in primary AML samples bearing different genetic aberrations, we identified two distinct patient subsets, based on their post-treatment senescence response. The activation of a senescent program was accompanied by a robust p53-DDR activation, heightened interferon signaling, and upregulation of HLA class I and II. These features were absent in the Sen Low samples. The correlation between senescence establishment and HLA molecules upregulation was also evident in an independent cohort of ex vivo treated AML samples and in a dataset of *NPM1*^-mut^ and del7 patients treated with chemotherapy in vivo, suggesting that this phenomenon is a common response triggered early upon chemotherapy across distinct AML subtypes.

By combining different functional immunological assays, we show that Sen High AML primary samples undergoing TIS elicited an effective CD8^+^ T cell activation, both ex vivo and in PDX models, likely due to HLA class I upregulation and increased display of AML/senescence-linked antigens, in line with evidence that senescence enhances tumor immunogenicity and antigen presentation^25^. This led to enhanced leukemia eradication, in line with recent reports showing that senescence triggers CD8^+^ T cell anti-tumor immunity^23–26^, supporting the idea that immune recognition of senescent cells by CD8^+^ T cells is conserved in hematological cancers. HLA class II molecules play a central role in the control of adaptive immune responses through the activation of CD4^+^ T cells^24,49^. Intriguingly, our data show that HLA class II upregulation in senescent AML blasts is associated with increased CD4 + T cell activation, as evidenced by their enhanced proliferation. This points to a potential role of CD4^+^ T cells in the anti-leukemic response, an aspect largely investigated for CD8^+^ T cells in solid tumors and lymphomas, but likely of relevance in anti-leukemia immune control. The involvement of CD4^+^ T cells in AML control, difficult to assess at the time of diagnosis or upon chemotherapy, is robustly supported by the pivotal role of this cell subset in the graft-versus-leukemia effect of allo-HSCT^50,51^. Similarly, cytotoxic CD4^+^ T cells were shown to eliminate human cytomegalovirus glycoprotein B (HCMV-gB) expressing senescent fibroblasts in the skin^52^. Previous studies have characterized immune-related transcriptional programs in AML at diagnosis, showing that high expression of interferon-stimulated genes (ISGs) and HLA class II molecules is associated with poor prognosis and resistance to therapy^53,54^. However, our findings reveal a fundamentally distinct nature of immune activation in response to chemotherapy. Unlike this chronic, interferon-driven state observed in AML, which is typically linked to immune exhaustion and evasion, the phenomenon we describe arises specifically upon TIS and is associated with productive autologous T cell responses and leukemia clearance. Supporting this concept, recent work showed that downregulation of HLA class II antigen presentation occurs in Isocitrate dehydrogenase 1/2 (*IDH*) mutant AML following IDH inhibitor monotherapy^39^, highlighting an immune evasion mechanism consistent with our previous findings in post-transplantation relapse^55^.

We show that the immunogenic potential of Senescence High AML samples may be dictated by distinct epigenetic changes responsible for the upregulation of HLA molecules observed upon senescence induction. Indeed, quantitative nuclear proteomics showed higher levels of the repressive histone modification H3K27me3 in Sen Low compared with Sen High samples. This was further supported by western blot analysis, which confirmed increased EZH2 and H3K27me3 levels, and by bulk RNA-seq data showing higher EZH2/PRC2-related transcriptional features in Sen Low blasts. Notably, Sen Low patients did not harbor mutations in the EZH2 gene, indicating that their repressive epigenetic landscape may arise from non-mutational mechanisms of PRC2 dysregulation. Consistent with this interpretation, our H3K27me3 CUT&Tag data provide chromatin-level evidence that immune-related loci, including interferon-associated genes and multiple HLA regions, are under PRC2-associated repressive control in Sen Low AML blasts and can be epigenetically remodeled by TAZ treatment. Together with the observed increase in HLA expression and enhanced T-cell proliferation, these findings suggest that PRC2-mediated repression may limit AML immunogenicity in this Sen Low subgroup.

From a translational perspective, our data support PRC2 inhibition with TAZ as an epigenetic immune-priming strategy that enhances AML immunogenicity and may increase responsiveness to T-cell-mediated therapies. This concept is in line with several studies, including our own, showing that PRC2 represses immune-related programs such as interferon signaling and HLA class I and II expression^55–58^, particularly in the context of post-allogeneic HSCT AML relapse. Moreover, EZH2 inhibition has been shown to remodel the pro-inflammatory SASP and, when combined with senescence-inducing therapies, can promote immune-mediated tumor control^59^, suggesting a correlation between senescence and epigenetic regulation of immunogenicity^60–62^. The immunomodulatory potential of targeting PRC2 is further supported by recent evidence showing that EZH2 inhibition sensitizes lymphoma cells to T cell immunotherapies^63^.

In recent years, ICB therapies have emerged as a revolutionary intervention for treating a variety of solid tumors by unleashing the cytotoxic potential of T cells against cancer cells^64^. Nevertheless, ICB has shown limited success in the context of AML^65,66^, where it has been mostly explored in late-stage, relapsed or refractory disease, often as monotherapy and generally in unselected patient populations^67^. Notably, slightly improved outcomes have been observed in first-line settings when combined with azacitidine in frail patients^68^. Here, we show that TIS synergizes with ICB to promote robust T cell activation in Sen High AML, whereas Sen Low samples remain unresponsive. These data suggest that the ability to activate the senescence program could serve as a biomarker to identify patients most likely to benefit from ICB-based regimens. Importantly, a similar synergy between TIS and ICB has been described in breast and other solid tumors^15^. Given that allo-HSCT represents the most widely clinically used form of adoptive immunotherapy to consolidate chemo-responsive AML patients^69^, it will be important to determine whether TIS-associated immunogenicity contributes to the lasting allo-mediated disease control or can potentiate emerging strategies like genetically engineered T cells. Along these lines, TIS establishment has emerged as a strong and reproducible predictor of chemotherapy response in previously untreated AML (Schönlein M. et al., Nature Communications, under invited revision). These observations highlight that incorporating TIS competence into routine diagnostic workflows could enhance patient stratification, identify likely non-responders early, and support more personalized therapeutic decisions in AML.

While our data highlight the short-term immunogenic benefits of TIS, previous studies, including ours^70^, have shown that the persistence of senescent cells can promote chronic inflammation, eventually contributing to leukemia stemness and chemoresistance^13,36^. Attempts to mitigate these long-term detrimental aspects of senescence in cancer have been the focus of intense scientific investigation^71^. SASP inhibitors may attenuate senescence-associated chronic inflammation that contributes to tumor growth and immune evasion, although their effects may be transient. Conversely, senolytic compounds or senolytic CAR-T cells can eliminate senescent cells in preclinical models^4,72,73^ and may potentially delay or prevent relapse if systemic organ and tissue toxicity is kept under control^74^. In conclusion, our findings uncovered a molecular link between TIS and leukemia immune recognition by the adaptive immune system, offering a strong rationale for senescence-based patient stratification and combination therapies aimed at promoting durable, immune-mediated responses in AML treatment.

## Methods

All research involving human samples and animals was conducted in compliance with all relevant ethical regulations. Studies involving human samples were approved by the San Raffaele Ethics Committee, IRCCS Ospedale San Raffaele, Milan, Italy, whereas animal studies were approved by the Animal Care and Use Committee of IRCCS Ospedale San Raffaele and authorized by the Italian Ministry of Health. Protocol-specific details are provided in the relevant Methods sections.

### Collection and purification of primary AML samples and treatment

Primary AML patient samples were collected and cryopreserved at the San Raffaele Leukemia Biobank after written informed consent was obtained, in accordance with the Declaration of Helsinki. The informed-consent documentation (obtained from all participants) specified that participant data could be disseminated through scientific publications only in strictly anonymized. Participants received no financial compensation.

The Leukemia Biobank protocol was approved by the San Raffaele Ethics Committee on 12 July 2010, with the latest amendment approved on 14 June 2012, and samples were analyzed according to the SEN-LEUK protocol, approved by the San Raffaele Ethics Committee on 10 December 2017, with the latest amendment approved on 14 March 2024.

Participant sex was obtained from clinical records. Both female and male participants were included based on sample availability, and sex was not used as an inclusion or exclusion criterion. Sex-stratified analyses were not performed because the individual experimental cohorts were small and the study was not designed or powered to assess sex-specific effects. Participant age and sex are reported in Supplementary Data 1.

After thawing, cells were cultured on a layer of irradiated (20 Gy) human fibroblasts (BJ-hTERT) in Blasts Culture Media (BCM) (components listed below). After 2 days of culturing, AML blasts were purified by a CD34- or CD33- based magnetic-activated cell separation system (for CD34: cat#130-046-702, for CD33: cat#130-045-501, Miltenyi Biotec) or by FACS sorting using the following antibodies: Brilliant Violet 510™ hCD45 (cat#368526, BioLegend), CD33-FITC (cat#IM1135U, Beckman Colter), CD34-PE (cat#130-113-179, Miltenyi Biotech). Following purification, AML cells were seeded for subsequent analysis.

### Blasts colture medium (BCM) preparation

BCM media was prepared with 250 ml DMEM (#cat D5671, Sigma-Aldrich; supplemented with 10% FBS, 1% Penicillin/Streptomycin mixture (P/S), and 2% L-Glutamine), 250 ml RPMI 1640 (cat#10-040-CV, Corning; supplemented with 10% FBS, 1% P/S, 2% L-Glutamine), 0,5 mM 2-Mercaptoethanol (cat#31350010, Thermo Scientific). Then, BCM was supplemented fresh with 20% 5637 cells-conditioned BCM and with the following cytokines:

**Table:** 

	Stock concentration (ng/µl)	Concentration in Cytokine BCM (ng/ml)	Catalog Number, brand
SCF	20	100	300-07-250ug, PeproTech
IL-3	10	10	200-03, PeproTech
IL-6	10	20	200-06-100ug, PeproTech
TPO	10	10	300-18, PeproTech
FLT-3-Ligand	10	10	300-19, PeproTech

### AML primary samples treatments

For the dose-response curve, AML blasts were plated in a 24 well plate onto irradiated BJ-hTERT stromal cells and escalating doses of AraC (cat#F000046284, Pfizer) were tested: C1: 1 nM; C2: 40 nM; C3: 400 nM; C4: 1 µM; C5: 4 µM. The drug was administered at 0 and 72 hours from AML blast thawing. At 72 hours, the cell medium was refreshed, the stromal layer replaced before adding the second dose and downstream analyses were performed at 120 h. Tazemetostat (cat#S7128-10mg, Selleck Chemicals) was used at 10 µM and administered at times 0, 24, 48,72 and 96 h.

### Cell lines and senescence-inducing treatments

BJ hTERT human fibroblasts (ATCC, cat#CRL-3627) were maintained in high-glucose Dulbecco’s modified Eagle’s medium (DMEM; cat#D5796, Sigma-Aldrich) supplemented with 10% FBS, 1% P/S and 1% sodium pyruvate (cat#11360-039, Gibco, 100 mM stock solution) at 37 °C and 5% CO₂. THP-1 cells (ATCC, cat#TIB-202) were maintained in RPMI 1640 (cat#10-040-CV, Corning) supplemented with 10% FBS, 1% P/S, and 2% L-Glutamine at 5% CO_2_ and 37 °C.

THP-1 and BJ hTERT cells were checked against the ICLAC Register of Misidentified Cell Lines, version 14, and neither cell line was listed as misidentified. The cell lines were not independently authenticated in our laboratory; their identities were based on the documentation provided by ATCC. 5637 cells were provided by collaborators and were used solely to generate conditioned medium. Information on the original source and catalog number was not available.

For the dose-response curve, cells were seeded 1 × 10^5^/ml in a 12-well plate, 5 technical replicates for each condition and treated with escalating doses of AraC (cat# F000046284, Pfizer; C1: 2 nM; C2: 20 nM; C3: 200 nM; C4: 2 µM; C5: 5 µM; C6: 10 µM) every other day for 7 days.

Subsequent readouts were performed with the selected dose of AraC (200 nM) after seeding cells in 6-well plates at 1,5 × 10^5^ cells/mL. Cells were counted with trypan blue solution using the TC20 counter every other day. Stained cells were acquired at the CANTO II analyzer, and data were analyzed using FlowJo version 10.10.0 (Tree Star).

For apoptosis, the staining was performed using Annexin V binding buffer 1x (cat#556454, BD), adding 2 μL of Annexin V (cat#640920, BioLegend) and 1 μL of 7AAD (Stock concentration: 50 µg/ml). The gating strategy was performed using FMO controls for Annexin V and DAPI with a sample containing a mix of control and treated cells.

Stained cells were analyzed at the CANTO flow cytometer, and data were analyzed using the FlowJo program. Senescence and HLA class I and II levels evaluation were carried out as described below.

### Senescence evaluation

Patients-derived AML cells and BM cells from in vivo experiments were incubated with Chloroquine (cat#C6628, Sigma-Aldrich,150 µM final concentration) for 1 h at 37 °C with 5% CO_2_. Then, 5-Dodecanoylaminofluorescein Di-β-D-Galactopyranoside (C_12_FDG, cat#D2893, ThermoFisher, final concentration 16,66 nM) was added for 30 min at 37 °C. Alternatively, for in vivo experiments, SPiDER-βGal (cat#SG02-10, Dojindo, final concentration 0.57 ng/uL) was used as senescence substrate and added for 15 min at 37 °C. After washing in PBS, stained cells were analyzed by imaging-based flow cytometry technology (ImageStreamX) or by standard flow cytometry at the CANTO II analyzer.

THP-1 cells were incubated in McIlvaine buffer (pH 6.0) with C_12_FDG at 16,66 nM working concentration) for 30 min at 37 °C. For McIlvaine buffer preparation, the protocol provided by Dojindo was used.

To measure senescence with ImageStreamX analysis, cells were first incubated with Edu (2 µM final concentration) for 48 h and stained for fluorescent SA-β-GAL, then with Pacific Blue AnnexinV (cat#640918, BioLegend) using Annexin V binding buffer 1x (cat#556454, BD). Cells were fixed, permeabilized and stained for EdU (Click-iT™ EdU Alexa Fluor™ 647 Flow Cytometry Assay Kit, cat#C10635, ThermoFisher). Only events with a gradient value higher than 60 based on brightfield images were considered cells on focus. Single-cell events were selected by plotting the area against the aspect ratio. Alive cells (negative for AnnexinV) within the single-cell gate were selected for the EdU signal and the senescence marker fluorescent SA-β-GAL.

### Western blot analysis

Cells were lysed in ice-cold RIPA buffer supplemented with Protease and Phosphatase Inhibitor Mini Tablets (#catA32961, Thermo Scientific) and sonicated with Bioruptor sonicator (Diagenode). Protein concentration was determined by Pierce™ BCA Assay Kit (cat#23227, Thermo Fisher Scientific) according to the manufacturer’s instructions. Proteins were resolved on pre-cast 4–15% Mini-PROTEAN gels (cat#4568084, Bio-rad) by SDS-PAGE, then transferred to a nitrocellulose membrane (cat#1620115, Bio-rad), followed by blocking with 5% BSA or 5% milk in TBS-Tween 0.1% (TBST) for 1 hour at room temperature. Membranes were incubated overnight at 4 °C with the specific primary antibody diluted in 5% BSA/TBST (cat#A7906-100G, Sigma-Aldrich) or 5% MILK/TBST (cat# 9999S, CST) according to antibodies datasheet. HRP-conjugated primary antibodies used as loading controls, including H3 and β-Actin, were incubated for 1 h at room temperature. Membranes were washed three times with TBST for 10 min RT on a shaker, then incubation with HRP-linked secondary antibody was carried out for 1 h at RT (according to primary antibody host species: Goat anti-Rabbit IgG (H + L) Secondary Antibody, HRP, cat#65-612-0, ThermoFisher). Membranes were developed using an HRP substrate (Clarity™ Western ECL Substrate, cat# 1705061, Bio-rad) and visualized at the iBright Imaging System (Thermo Fisher). Protein size was determined by matching bands from a colored protein ladder (cat#26619, Thermo Scientific). Protein bands were quantified using ImageJ software. Differences in protein levels were normalized against the indicated loading control.

A list of the primary antibodies used is reported in the table below:

**Table:** 

Antibody	Species	Dilution	Reference
p21	Rabbit	1:500	cat#2947 s, CST
γH2AX	Rabbit	1:500	cat#ab81299, Abcam
H3K27me3	Rabbit	1:500	cat#9733 s, CTS
EZH2	Rabbit	1:1000	cat#4905 s, CST
β-actin-HRP		1:25000	#catA3854-200UL, Sigma-Aldrich
H3-HRP		1:5000	Cat#ab21054, Abcam

### Sample preparation for immunopeptidomics

Patient-derived AML blasts from *n* = 3 biologically independent Sen High and *n* = 3 biologically independent Sen Low patient samples were cultured as described above and analyzed under untreated and AraC-treated conditions. Cells were washed twice with ice-cold PBS by centrifugation at 335 × *g* for 5 min at 4 °C. After the first wash, cells were transferred to low-protein-binding microcentrifuge tubes. Following complete removal of the supernatant, cell pellets were weighed, snap-frozen in liquid nitrogen, and stored at − 80 °C until further processing.

### Purification and concentration of HLA class I peptides

HLA class I-bound peptides were immunoaffinity purified from AML samples using biotin-conjugated anti-human HLA-A, HLA-B, and HLA-C antibodies (cat#311434, clone W6/32, BioLegend). For sample preparation, the snap-frozen cell pellet was resuspended in the lysis buffer [150 mM NaCl, 50 mM TRIS-HCl, pH 7.4, protease inhibitors (cat#A32955 Thermo Scientific Pierce), 1% Igepal (cat#I8896, Sigma-Aldrich)]. Lysates were centrifuged for 10 min at 500 × *g*, and then the supernatant was centrifuged for 30 min at 25 000 × *g*. Next, HLA-I complexes were immunoaffinity purified from the cleared lysate with anti-human HLA-A, HLA-B, and HLA-C biotin–streptavidin bound to the micropillars of the biotinylated thiol–ene CHIP (PeptiChip^75^). The CHIPs were first washed three times with PBS, and then the HLA molecules were eluted at room temperature by adding acetic acid 7% (cat#A113, Fisher Scientific) in 50% MeOH (cat#10402824, Fisher Scientific). Eluted HLA peptides and the subunits of the HLA complexes were desalted using SepPac-C18 cartridges (Waters)^76^. Briefly, the cartridge was prewashed with 80% acetonitrile in 0.1% trifluoroacetic acid (TFA) and then with 0.1% TFA. The peptides were purified from the HLA-I complex by elution with 30% acetonitrile in 0.1% TFA. Finally, the samples were dried using vacuum centrifugation (Eppendorf).

### LC-MS analysis of HLA-I peptides

Each dry sample was dissolved in 10 μL of LC-MS solvent A (0.1% formic acid). The nanoElute LC system (Bruker) injected and loaded 10 μL of the sample directly onto the analytical column (Aurora C18, 25 cm long, 75 μm ID, 1.6 μm bead size, Ionopticks), constantly kept at 50 °C by a heating oven (Column Toaster, Bruker). After washing and loading the sample at a constant pressure of 800 bar, the LC system started a 30 min gradient from 0% to 32% solvent B (acetonitrile, 0.1% formic acid), followed by an increase to 95% B in 5 min, and finally a wash of 10 min at 95% B, all at a flow rate of 300 nL/min. Online LC-MS was performed using a Tims TOF SCP mass spectrometer (Bruker) operated with the CaptiveSpray source, capillary voltage 1500 V, dry gas flow of 3 L/min, dry gas temperature at 180 °C. Mass range was 100–1700 m/z, and mobility range was 0.6–1.70 V.s/cm2. MS/MS was used with three PASEF (parallel accumulation – serial fragmentation) scans (with 300 ms ramp time) per cycle with a target intensity of 20 000 and intensity threshold of 500, considering charge states 0–5. Active exclusion was used with release after 0.4 min, reconsidering precursor if the current intensity is greater than fourfold the previous intensity, and a mass width of 0.015 m/z and a 1/k0 width of 0.015 V.s/cm2. Isolation width was defined as 2.00 m/z for mass 700 m/z and 3.00 m/z for mass 800 m/z. Advanced collision energy settings consisted of nine points of collision energy dependency on ion mobility: 0.70 V.s/cm2 − 27.50 eV; 0.85 V.s/cm2 − 32.00 eV; 0.90 V.s/cm2 − 36.00 eV; 0.95 V.s/cm2 − 38.00 eV; 1.25 V.s/cm2 − 44.00 eV; 1.30 V.s/cm2 − 46.00 eV; 1.35 V.s/cm2 − 47.00 eV; 1.50 V.s/cm2 − 47.00 eV; 1.65 V.s/cm2 − 50.00 eV. Precursor ions were selected using 1 MS repetition and a cycle overlap of 1 with the default intensities/repetitions schedule.

Each of the six biologically independent AML patient samples was analyzed under untreated and AraC-treated conditions, resulting in 12 samples. Each sample was acquired once by LC–MS/MS, with no technical replicate acquisitions.

### Immunopeptidomics database search and peptide identification

MS/MS spectra were analyzed using PEAKS Studio 11.0 (build 20230414; Bioinformatics Solutions Inc.). A database-independent de novo search was first performed using the PEAKS DeepNovo algorithm, followed by a peptide database search using a target–decoy strategy. Spectra were searched against the UniProt human reference proteome, including isoforms (103,789 entries; downloaded on 26 April 2023). No enzyme specificity was imposed, and the digestion mode was set to unspecific. Precursor and fragment ion mass tolerances were set to 20 ppm and 0.02 Da, respectively. No fixed modifications were specified. Methionine oxidation was included as a variable modification, allowing a maximum of three oxidations per peptide. Peptide identifications were filtered using a peptide-level false discovery rate of 5%. Binding affinity to the corresponding patient-specific HLA alleles was predicted for all eluted peptides identified in the AML samples using NetMHC 4.0. Strong binders were defined as peptides with a %rank < 0.5, weak binders as peptides with a %rank ≥ 0.5 and < 2, and non-binders as peptides with a %rank ≥ 2. Strong and weak binders were retained for downstream analyses.

### T cell purification

Whole peripheral blood samples from either healthy donors or AML patients were processed to obtain mononuclear cells (PBMCs) through density gradient centrifugation using Lympholyte™ (cat#DVCL5020-10, Lonza). CD3^+^ T lymphocytes were isolated via magnetic separation with a Pan T cell Isolation Kit (cat#130-096-535, Miltenyi Biotech) according to the manufacturer’s instructions. The negative fraction was collected. After the selection, viable samples consisted of >90% CD3^+^ T cells. T cells composition (CD4^+^ and CD8^+^) was measured via FACS.

### Mixed Lymphocyte Reaction (MLR) and downstream analyses

Purified T cells, autologous or coming from healthy donors, were stimulated with the THP-1 cell line (pre-treated for 48 hours with 200 U/mL Recombinant Human IFN-γ [cat#300-02, PeproTech]) or with AraC-treated primary blasts from either Sen Low or Sen High samples at an effector: target ratio of 1:1. Cells were cultured in IMDM supplemented with 2% L-glutamine, 1% P/S, 10% Human Serum (cat#ECS0219D, Euroclone), and IL-2 (cat# 201-LB-100/CF, R&D Systems, 50 ng/mL final concentration). IL-2 was replaced every 3–4 days. After stimulation, T cells were tested against targets of choice, co-culturing them at a 1:1 ratio. To block HLA-DR, cells were pre-treated with 10 μg/mL HLA-II blocking antibody (Bio X Cell, BE0306) or 10 μg/ml isotype IgG antibody in the absence of serum overnight at 37 °C. For experiments with ICBs, blasts were treated with 10 μg/mL Ipilimumab (anti-CTLA-4, cat#A2001, Selleck Chemicals), Nivolumab (anti-PD-1, cat#A2002, Selleck Chemicals) or Durvalumab (anti-PD-L1, cat# A2013, Selleck Chemicals) before starting the co-culture. T-cells were then collected at different time points for proliferation, degranulation and activation markers expression. For T cell proliferation, before co-culture T cells were resuspended 1 × 10^6^ cells/mL in 1X PBS containing CellTrace Violet Cell (cat#C34557, ThermoFisher) for 20 minutes at 37 °C, then washed with supplemented IMDM. Dilution of Cell Trace Violet was quantified by FACS analyses 5 days after co-culture using primed T cells cultured alone as a negative control. T-cell degranulation was evaluated by adding an anti-CD107a antibody at the beginning of co-culture (CD107a-FITC clone H4A3, cat#555800, BD) and Golgi Stop (cat#554724, BD) after 3 h. At 6 h after the co-culture start, cells were stained with CD8-PECy7 or APC-Cy7 (depending on the presence of CD33 Ab) and CD4-PE antibodies, then the signal was acquired at FACS Canto II.

### T cell ex vivo expansion protocol

2 × 10^^6^ PBMCs from AML primary samples BM vials were cultured in G-Rex24 Well Plate (cat#80192 M, Wilson Wolf) in X-Vivo 15 medium (cat#BEBP02-054Q, Lonza) supplemented with 5% human serum, 1% P/S, 100 IU/mL IL-7 (cat# BEBP02-054Q, Miltenyi Biotec), 200 IU/mL IL-15 (cat#130095764, Miltenyi Biotec), and 10 ng/mL IL-2 (cat#130097743, Miltenyi Biotec) with the addition of T Cell TransAct™ (cat#130-111-160, Miltenyi Biotec) (40uL per million of CD3^+^ cells measured by FACS analysis). 24 h post TransAct™ addition, new X-Vivo media was added, reaching a final volume of 5 mL. Cells were kept in culture for three weeks, then counted and used; the medium was changed at least once a week.

### Genetic engineering by CRISPR-Cas9

THP-1 cells were nucleofected with 2.5–1.25 µM of Ribonucleoproteins (RNPs) and electroporation enhancer (cat# 1075916, Integrated DNA Technologies) according to the manufacturer’s instructions using P3 Primary Cell 4D-Nucleofector X Kit and program EA-100 (Lonza). RNPs were assembled by incubating for 10 min at RT at 1:1.5 M ratio S.p. Cas9 protein (cat# 9214-5MG, Aldevron) with a synthetic sgRNA for B2M (Hs.CAS9.B2M.1.AB; sequence: 5- mAmAmG rUrCrA rArCrU rUrCrA rArUrG r, IDT). Cells experiencing sole electroporation or edited with an RNP complex (indicated as CTRL in the Figures) were employed as controls. Editing efficiency was measured by assessing the expression of HLA class I by FACS analysis.

Subject to availability, frozen stocks of the newly generated B2M-edited THP-1 cell populations are available from the corresponding author upon reasonable request and under an appropriate material transfer agreement.

### Sample preparation for histone PTM mass spectrometry

Patient-derived AML blasts from *n* = 3 biologically independent Sen High and *n* = 3 biologically independent Sen Low samples were cultured as described above. For each analysis, 1 × 10⁶ cells were collected. Each patient sample was analyzed in two independent experiments performed on separate days, resulting in *n* = 6 measurements per group. Cell pellets were washed twice with ice-cold PBS using a refrigerated centrifuge, snap-frozen in liquid nitrogen, and stored at − 80 °C until further processing.

### Histone PTMs mass spectrometry analysis

Histones were enriched from 1 × 10^6^ cells^77^. Approximately 4 µg of histone octamer were mixed with an equal amount of heavy-isotope labeled histones, which were used as an internal standard (super-SILAC mix)^78^ and separated on a 17% SDS-PAGE gel. Histone bands were excised, chemically acylated with propionic anhydride, and in-gel digested with trypsin, followed by peptide N-terminal derivatization with phenyl isocyanate^79^. Peptide mixtures were separated by reversed-phase chromatography on an EASY-Spray column (Thermo Fisher Scientific), 25 cm long (inner diameter 75 µm, PepMap C18, 2 µm particles), which was connected online to a Q Exactive Plus instrument (Thermo Fisher Scientific) through an EASY-Spray™ Ion Source (Thermo Fisher Scientific), as described^79^. The acquired raw data were analyzed using EpiProfile 2.0^80^, selecting the SILAC option, followed by manual validation. For each histone-modified peptide, the percentage relative abundance (%RA) in the sample (light channel, L) and in the internal standard (heavy channel, H) was estimated as described previously^79^. L/H ratios of %RA were then calculated. The %RA for each peptide was determined by summing all peptide forms containing a given PTM. The mass spectrometry data have been deposited to the ProteomeXchange Consortium^81^ via the PRIDE partner repository with the dataset identifier PXD065846.

### LC–MS/MS acquisition for histone PTM analysis

Solvent A consisted of 0.1% formic acid (FA) in ddH₂O, whereas solvent B consisted of 80% acetonitrile (ACN) and 0.1% FA. Peptides, resuspended in aqueous 1% trifluoroacetic acid (TFA), were loaded at a flow rate of 500 nL min⁻¹ and separated using a 50 min linear gradient from 10% to 45% solvent B. The Q Exactive Plus mass spectrometer was operated in data-dependent acquisition (DDA) mode, automatically switching between full-scan MS and MS/MS acquisition. Full-scan MS spectra were acquired over an *m/z* range of 300–1350 in the Orbitrap detector at a resolution of [confirm value] at *m/z* 200. The [confirm whether 10 or 12] most intense precursor ions with charge states ranging from 2 to 4 were sequentially selected for higher-energy collisional dissociation (HCD) using a normalized collision energy of 28%. The automatic gain control targets were set to 3 × 10⁶ for MS1 and 1 × 10⁵ for MS/MS, with maximum injection times of 20 ms and 80 ms, respectively. Dynamic exclusion was set to 10 s. The spray voltage was 1.8 kV, with no sheath or auxiliary gas flow. Each sample preparation was analyzed once by LC–MS/MS, without technical replicate acquisitions.

### CUT&Tag assay

CUT&Tag experiments were performed following the protocol previously described by Okur et al.^82^. with minor adaptations for low-input primary AML samples. Briefly, untreated or Tazemetostat-treated primary AML blasts were collected, and the nuclei were extracted by incubating cells for 10 minutes on ice in 100 µL/sample of cold Nuclei Extraction buffer (NE buffer: 20 mM HEPES-KOH pH 7.9, 10 mM KCl, 0.1% Triton X-100, 20% Glycerol, 0.5 mM Spermidine, 1X Protease Inhibitor cocktail). Nuclei were then bound to activated ConA beads (cat#21-1401, EpiCypher). Nuclei were then incubated with an anti-H3K27me3 primary antibody (cat#13-0055, EpiCypher), followed by secondary antibody incubation (cat#13-1047, EpiCypher) and pAG-Tn5 transposase-mediated tagmentation (cat#15-1017, EpiCypher), according to the published protocol. Libraries were amplified, purified, and sequenced.

### In vivo patient-derived xenografts experiments

Female NSG mice (*Mus musculus*; NOD.Cg-*Prkdc*^scid^ *Il2rg*^tm1Wjl^/SzJ; NOD genetic background), aged 8–10 weeks, were purchased from Charles River Laboratories (Calco, Italy) and maintained under specific-pathogen-free conditions, with a 12 h light/12 h dark cycle, at 22 ± 2 °C and 55 ± 5% relative humidity.

All animal procedures were approved by the Animal Care and Use Committee of IRCCS Ospedale San Raffaele and authorized by the Italian Ministry of Health in accordance with Italian legislation (IACUC protocol no. 1386). NSG mice were sub-lethally irradiated (150 rad) 4–6 h prior to transplantation. Primary AML blasts were suspended in 150 μl PBS and transplanted by tail vein injection into 8–10-week-old NSG recipients.

Disease engraftment was monitored by periodic peripheral blood sampling and/or bone marrow (BM) aspirates. Mice were euthanized at predefined experimental time points, corresponding to one week after AraC or tazemetostat treatment (TP1) or after one or two T-cell infusions (TP2), depending on the experimental arm. Throughout the study, animals were closely monitored for body weight and clinical signs of distress in accordance with the approved IACUC protocol and were to be euthanized immediately if predefined humane endpoint criteria were met. No animals reached the humane endpoint criteria before the scheduled experimental endpoint.

BM cells were harvested at 16–24 weeks post transplantation by flushing femurs and tibiae under sterile conditions, filtered through a 40 μm cell strainer and resuspended in ice-cold PBS  +  2% FBS. For the chemotherapy experiments, NSG mice were treated with AraC (cat#F000046284, Pfizer) at a dose of 100 mg/kg by subcutaneous injection on days 1 to 5 of the treatment regimen. The drug was prepared in a saline solution. Control animals received an equal dose of saline solution. Mice were allocated to treatment or control groups with equal AML burden based on a pre-treatment BM aspirate. Leukemia burden was measured as the total number of cells retrieved from the bone marrow multiplied by the percentage of human CD45⁺ cells determined by flow cytometry. Leukemia-free mice were defined as mice with less than 1% of human CD45^+^ cells.

For the Tazemetostat in vivo experiment, EPZ-6438/TAZ (#cat. S7128, Selleckchem) was resuspended in 10% DMSO, 40% PEG300, 5% Tween-80, and 45% saline solution, and administered by oral gavage at 300 mg/kg once daily for 7 consecutive days. Control mice received vehicle alone following the same schedule.

### T cells and ICB in vivo infusion

Expanded CD3^+^ T cells were injected 1 × 10^6^ cells per mouse once a week for 2 weeks, starting 2 days after the stop of the chemotherapy regimen. T cells were resuspended in PBS and transplanted by tail vein injection. Nivolumab was administered by tail vein injection at a dose of 200 μg/mouse on the same day of T cell injection. In the Tazemetostat in vivo setting, only one infusion of expanded CD3^+^ T cells (1 × 10^6^ cells per mouse) was performed the day after the last dose of TAZ administration.

### Flow cytometry panels

For immunophenotypic analysis, a maximum of 0.5 × 10^5^ cells per tube were stained in 100 μl of 1X PBS, 2% FBS, plus the mix of antibodies. Staining was performed at RT for 15 min, followed by washing with 2 ml of 1X PBS, 2% FBS before the analysis. Samples were acquired using the FACS Canto II analyzer (BD Biosciences).

Here, is the complete list of antibodies used for HLA class I and II evaluation (all BioLegend): HLA-ABC Pacific Blue (Clone W6/32, cat#311418), HLA-DR FITC (Clone L243, cat#307604) or HLA-DR APC-Cy 7 (cat#307618).

Here is the complete list of antibodies used for experiments of T cell activation: CD4 FITC (cat#563550, BioLegend), CD8 APCH7 (cat#641400, BD), CD69 APC (cat#310910, BioLegend), CD25 PE (cat#130-113-286, Miltenyi Biotec). The signal was acquired using the FACSymphony^TM^ flow cytometer (BD Bioscience).

Here, is the complete list of antibodies used for experiments of co-culture between T cells and AML cell lines: CD33 (cat# 333952, BD Biosciences), CD8 APC-Cy7 (cat# 344714, Biolegend), CD4 FITC (cat#980802, BioLegend) or CD4 PE (cat#300508, BioLegend) when combined to T-cells degranulation assay, CD69 APC (cat#310910, BioLegend), CD25 PE (cat#130-113-286, Miltenyi Biotec), ZOMBIE Aqua (cat#423102, BioLegend). For the exhaustion markers panel, we used the following antibodies: TIM3 APC (cat#345011, BioLegend), LAG-3 Brilliant Violet 605 (cat#369324, BioLegend), PD-1 PE (#cat329706, BioLegend). For the T cells subsets panel in Supplementary Fig. S4B we used the following antibodies: CD3 APC (cat#130-113-125, Miltenyi Biotec), CD95 PeCy7 (#cat 305622, BioLegend), CD62L PerCPCy5.5 (cat# 304824, BioLegend), CD45RA VioBlu (cat# 130-113-360, Miltenyi Biotec). For the T cells subsets panel in Supplementary Fig. S4K we used the following antibodies: CD3 PcpCy5.5 (cat#300328, BioLegend), CD8 APC-H7 (cat#641400, BD), CD4 BV605 (cat#562658, BD), CD45RA PB (cat#304123, BioLegend), CD45 BUV395 (cat#563792, BD), CD62L BUV737 (cat#568329, BD), Fixable Viability Dye Viakrome 808 (cat#C36628, Beckman Colter). Naïve: CD45RA^+^CD62L^+^CD95; stem cell memory: CD45RA^+^CD62L^+^CD95^+^; central memory: CD45RA^-^CD62L^+^CD95^-^; effector memory: CD45RA^-^CD62L^-^CD95^-^; terminal effector: EFF; CD45RA^+^CD62L^-^CD95^-^:

For immunophenotypic analyses of cells retrieved from PDX, we used antihuman FcR Blocking (cat#130-059-901, Miltenyi Biotec) and antimouse FcR Blocking (cat#553142, BD) and then cells were stained with the following antibodies: mouse CD45 FITC (cat#103108, BioLegend) and human CD45 (cat#304016, BioLegend).

Data were processed using FlowJo version 10.10.0 (Tree Star).

### Total RNA-Seq library preparation and sequencing

Total RNA was isolated with miRNeasy Mini Kit (cat#217004, QIAGEN), according to the manufacturer’s instructions. RNA was quantified with The Qubit 2.0 Fluorometer (ThermoFisher), and quality was assessed by the Agilent 4200 TapeStation (Agilent Technologies). To perform RNA sequencing on Sen High and Sen Low patients treated with AraC (or untreated), only samples with RNA integrity number (RIN) > 7 were used, and 10–30 ng of total RNA was used for library preparation with Illumina Stranded mRNA Prep Ligation and sequenced on a NextSeq 500 (Illumina). To perform RNA-Seq on Sen Low samples treated with TAZ (or untreated), library preparation was performed with the NEBnext ultra II RNA directional library preparation kit, with the respective rRNA depletion module.

### Bioinformatics analysis

High-quality reads were obtained after reads quality inspection and adapter trimming with Trim Galore (https://www.bioinformatics.babraham.ac.uk/projects/trim_galore/). Reads were aligned to the human genome (GENCODE version hg38 primary assembly) with STAR release 2.7.6a. Gene expression quantification was obtained with featureCounts v2.0.1. The differential expression (DE) analysis was performed with the DESeq2 package (v1.50.2), filtering out genes with zero count for the comparison Sen High untreated versus Sen Low untreated samples (adjusted *p*-value 0.05). To test the transcriptomics profile differences between the senescence phenotypes upon treatment, we applied an interaction analysis after filtering out lowly expressed genes up to ten reads count. In the design formula, we took into account the paired setup of the experiment ( ~ phenotype + phenotype:patient.n + phenotype:condition), focusing on the contrast in the interaction term (*i.e*., phenotypeSen_high.conditionAraC vs phenotypeSen_low.conditionAraC). Genes with adjusted *p*-values < 0.1 (Benjamini-Hochberg correction method) were considered differentially expressed. Gene Set Enrichment Analysis (GSEA) was performed using the clusterProfiler package (v4.18.4), ranking genes according to the Log_2_FC values of the comparison. In particular, we tested the hallmark gene set version 7.2 from MSigDB. Furthermore, to assess the expression of genes linked to dendritic cell phenotypes upon treatment (Sen High AraC vs Sen Low AraC), we performed GSEA using the Villani et al. dataset^37^. Senescence classical signatures^28–35^ were tested in the interaction model and in the comparison between Sen Low samples upon Tazemetostat treatment versus Sen Low samples untreated. The analysis for the detection of HLA (class I and II) was performed by aligning high-quality reads to the human genome (GENCODE version GRCh38.p13, including patches and haplotypes) with a multi-mapping approach, considering the following parameters for the alignment with STAR ‘--winAnchorMultimapNmax 100 --outFilterMultimapNmax 100’, and for the expression quantification with featureCounts ‘-M --fraction’. The interaction analyis was performed following the same design as described above. For the downstream analysis of HLA genes, we selected HLA class I and class II genes that were upregulated upon treatment in both groups, using a nominal p-value < 0.1 as selection criteria.

Regarding the interaction analysis, in order to recover the real direction of change between the phenotypes at the treatment, for each patient we calculated gene-wise the log_2_ fold change of the treated over the untreated sample as such:

1 $${\log }_{2}F{C}_{{pz},g}={\mbox{rlog}}\left({\mbox{Ara}}{{\mbox{C}}}_{{\mbox{pz}},{\mbox{g}}}\right)-{\mbox{rlog}}\left({{\mbox{UT}}}_{{\mbox{pz}},{\mbox{g}}}\right)$$

The values were averaged gene-wise per phenotypic group:

2 $${\overline{{\log }_{2}FC}}_{{\mbox{phen}},g}=\frac{1}{N}{\sum }_{{pz}=1}^{N}{\log }_{2}F{C}_{{pz},g}$$

where *g* denotes the genes, *pz* indexes the patients and *N* is the number of patients in each phenotypic group (*i.e*., *phen*) ∈{*Sen High*, *Sen Low*}. Next, for each significant gene from the interaction analysis, we recalculated an interaction-log_2_FC between phenotypes:

3 $${x}_{g}={\bar{y}}_{g}-{\bar{z}}_{g}$$

(*i.e*., y = log_2_ Sen High_AraC/UT._ and z = log_2_ Sen Low_AraC/UT_). With a custom function (*find_directions*), which considers individually the sign of the log_2_FC within each phenotype, we recovered the direction of gene deregulation with respect to the first term of the contrast. By calculating all the possible combinations of signs, we grouped the interaction-log_2_FC in at most six modules and assigned each significant gene exclusively to one of them:

4 $$\begin{array}{c}{x}_{g}={\overline{y}}_{g}-{\overline{z}}_{g}=\\ \left\{ \begin{array}{cc}\overline{y} > 0,\,\overline{z} > 0,\,\overline{y} > \overline{z} & up-regulated\,in\,y\,(homodirectional) \\ x > 0\, \left\{\overline{y}\right. < 0,\overline{z} < 0,\, \overline{y} > \overline{z} & down-regulated \, in \, z\,(homodirectional) \\ \overline{y} > 0,\,\overline{z} < 0\, & discordant,\,up\,in\,y \\ \overline{y} < 0,\,\overline{z} < 0,\,\overline{y} > \overline{z} & down-regulated\,in\,y\,(homodirectional)\\ x < 0 \, \left\{\overline{y}\right. > 0,\,\overline{z} > 0,\,\overline{y} < \overline{z} & up-regulated\,in\,z\,(homodirectional)\\ \overline{y} < 0,\,\overline{z} > 0 & discordant,\,down\,in\,y\end{array}\right.\end{array}$$

where x is a given gene interaction-log2FC obtained by the log difference between the average log2$F{C}_{{phen}}$, calculated as before for each phenotypes. We distinguish between homodirectional cases where both contrasts have genes up-regulated, but (1) with significantly higher extent in the first phenotype of the contrast, (2) with higher extent in the second phenotype, and (3) we found divergent log_2_FC signs, with genes upregulated in one phenotype at treatment respect to the contrasted phenotype, and vice versa. The same categorization applies to down-regulate genes.

### scRNA-seq analysis

The analysis of the published on the scRNA-seq dataset GSE146590, was conducted following the method section of the paper^13^. Differential expression analysis between the AraC and untreated cells within clusters was performed with the Wilcoxon Rank Sum test provided by the Seurat package FindMarkers function (v3.2.2). HLA genes with log_2_FC > 0 and Bonferroni-adjusted *p*-value < 0.1 were considered significantly modulated. The expression of the significant genes was plotted per cell cluster with the function Dotplot. Pre-processed data from collaborators^36^ were used to check genes expression programs with the function AddModuleScore in the Seurat R package. Cells with module score ≥ to the third quantile value of the total distribution were showed as a contour chart. The distribution of the module scores was represented using ridge plots with the RidgePlot function provided in the Seurat R package. Deconvolution of bulk RNA-seq was performed with MuSiC (v1.0.0) method^70^ using as a reference the NPM1^mut^ single cell dataset.

Reference mapping analysis was conducted, with Seurat (v5.2.0), for the query scRNA-seq dataset GSE146590. As a reference, we tested two healthy datasets: a dataset of 8 healthy bone marrow samples from the geo series GSE116256^37^, and the human BoneMarrowMap^38^, which integrates 45 healthy donors. Furthermore, we tested as reference two diseased cohorts: the NPM1-mutant dataset^36^ (GSE185993) and the IDH-mutant dataset^39^ (GSE230559).

### Peptide analysis

The function query_uniprot (queryup v1.0.5 package) allowed to map the Uniprot ID associated to each peptides (length 8–12) to the corresponding gene name. Peptides matching possible contaminants were filtered out. Replicate samples were merged by donor. The function compareCluster, from the clusterprofile package, was adopted for the over-representation analysis of the union of genes associated with peptides per group. The databases KEGG, Reactome and Biological Process from GO terms, were respectively tested with enrichKegg, enrichPathway (ReactomePA v1.34.0) and enrichGO function (parameters pvalueCutoff = 0.05, pAdjustMethod = “BH”).

### CUT&TAG analysis

After adapter trimming with TrimGalore (v0.6.10, --quality 20, --length 25), alignment was performed with bowtie2 (v2.5.4, --local --very-sensitive --no-mixed --no-discordant --phred33 -I 10 -X 700), only properly paired reads, with no supplementay or secondary alignment, and mapping quality of at least 15 were retained for the subsequent steps. PCR duplicates were filtered out with picard MarkDuplicates tool (v3.1.1). ChrY, mithocondrial mapping reads and blacklisted regions (hg38 v2) were also filtered out. Macs2 (v2.2.9.1) with broad parameters was adopted for peak calling. A catalog of regions was calculated after merging the peaks between the untreated and tazemetostat treated sample (bedtools merge v2.31.1). The table of counts was calculated with (bedtools map), taking into account the fold enrichment file generated with macs2 bdgcmp function. As an exploratory analysis, the log2FC was calculated considering Tazemetostat (*n* = 1) vs Untreated (*n* = 1) in order to capture the direction of signal enrichment, and using a threshold of 1 to describe the trend of gain and loss regions. Chipseeker (v1.38.0) annotatePeak function was adopted for annotating all the catalog regions (with parameters sameStrand = FALSE, ignoreOverlap = FALSE, ignoreDownstream = FALSE, overlap = “all”, +/− 2000 bp around TSS and gencode v35). The track plots for rappresentative regions (ggcoverage v1.4.1) was generated over the RPKM normalized bigwig file computed with the bamCoverage function (deepTools v3.5.5).

### Statistical analysis

Data were summarized as median with IQR (or range) or mean ± s.e.m. according to data distribution. For the analysis related to senescence evaluation, HLA expression, MLR, in vivo PDX experiments, inferential procedures were applied only when permitted by the sample size and experimental design. Non-parametric tests were performed when at least 3 biologically independent observations were available, whereas linear mixed-effects models were fitted only for datasets including at least 5 biologically independent observations. Otherwise, descriptive statistics were reported.

Spearman’s correlation coefficient was computed to evaluate the presence of a monotonic relationship between variables. Comparisons between two independent groups were performed using the Mann‑Whitney test, whereas comparisons among more than two groups were performed using the Kruskal‑Wallis test, followed by Dunn’s post hoc test. When required, the Bonferroni correction was applied to adjust for multiple testing. For matched data, the Wilcoxon signed-rank test was used for comparisons between two groups, whereas the Friedman test followed by Dunn’s post hoc test was used for comparisons among more than two groups. In the presence of non-independent observations due to donors or experiments being shared across groups, random-intercept linear mixed-effects models (LME)^83^ were fitted, including a donor- or experiment-specific random effect, nested when necessary. When needed, outcome variables were transformed (logarithmic, square‑root, cubic‑root, or ordered quantile normalization) to meet LME assumptions. Post hoc pairwise comparisons between treatment groups were performed after LME, applying Bonferroni correction for multiplicity. Whenever a directional hypothesis was supported by prior biological evidence or experimental rationale, one-tailed tests were conducted; otherwise, two-tailed tests were used. The significance threshold was set at 0.05 for all analyses. Statistical analyses were performed using GraphPad Prism v.10 (GraphPad) and R software (v.4.4.1; https://cran.r-project.org/). Exact sample sizes and additional details for each analysis are reported in the corresponding figure legends and in Supplementary Table 1.

### Reporting summary

Further information on research design is available in the Nature Portfolio Reporting Summary linked to this article.

## Supplementary information

Supplementary Information

Description of Additional Supplementary Files

Supplementary Data 1

Supplementary Data 2

Supplementary Data 3

Supplementary Data 4

Supplementary Data 5

Reporting Summary

Transparent Peer Review file

## Source data

Source data

## Data Availability

The sequencing datasets generated in this study have been deposited in the NCBI Gene Expression Omnibus under SuperSeries accession GSE324154, which includes RNA-seq data from AraC-treated samples under accession GSE229736 and RNA-seq data from tazemetostat-treated samples under accession GSE324153. The CUT&Tag data have been deposited in the NCBI Gene Expression Omnibus under accession GSE336442. Histone post-translational modification mass spectrometry data have been deposited in the ProteomeXchange Consortium via the PRIDE repository under accession PXD065846. LC-MS immunopeptidomics data have been deposited in the ProteomeXchange Consortium via the PRIDE repository under accession PXD068731. Anonymized participant-level clinical and sample metadata are provided in Supplementary Data 1. Source data are provided in this paper.

## Code availability

The code used to generate the results reported in this study is publicly available at the following GitLab repository: http://www.bioinfotiget.it/gitlab/custom/aml_gilioli_fusco_2026.
