# Supplementary material for: Therapy induced senescence promotes immunogenicity in acute myeloid Leukemia through reduced EZH2 activity
Source: Nat Commun. 2026 Sep 4;17:9151. doi: 10.1038/s41467-026-76853-1 (PMC13545235; doi:10.1038/s41467-026-76853-1)
Supplement: Supplementary file 1 [file 41467_2026_76853_MOESM1_ESM.pdf]

Supplementary Information

Supplementary Figures

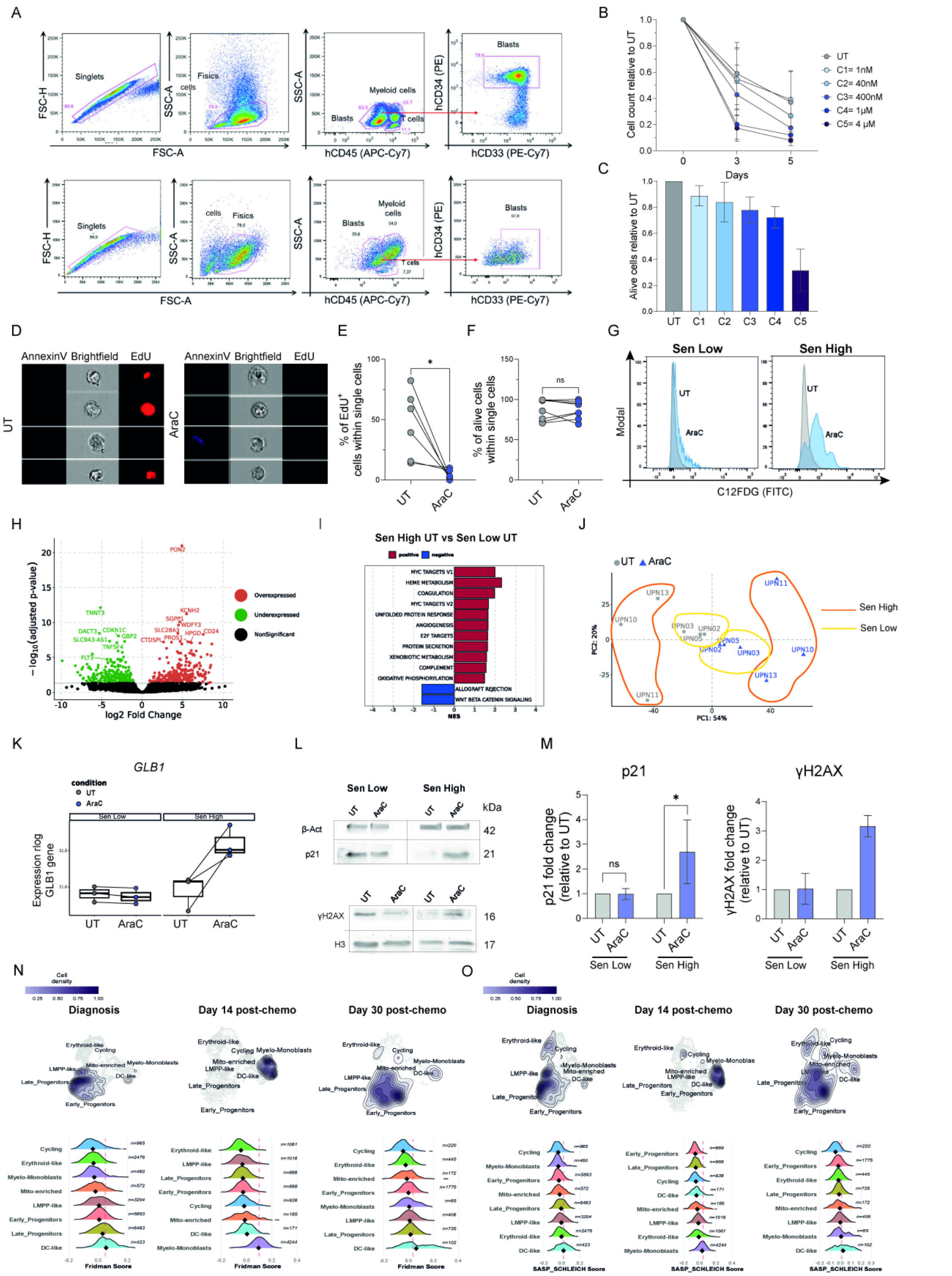

Fig. S1. Senescence evaluation in primary AML samples. A, Representative gating strategy for FACS purification of AML blasts. **B**, AraC dose-response relative to untreated (UT) blasts after

5 days (AraC added on days 0 and 3). Mean ± SEM; *n*= 3 biologically independent AML samples. **C**, Viable cells relative to UT, assessed by flow cytometry at the doses shown in B. Mean ± SEM; *n*=3 biologically independent AML samples. **D,** Representative ImageStreamX images of UT and AraC-treated blasts showing morphology (brightfield), apoptosis (Annexin V) and proliferation (EdU). **E**, **F,** EdU-positive cells (**E**; *n*= 6 biologically independent samples) and Annexin V-negative cells (**F**; *n*= 8 biologically independent samples) in UT and AraC-treated blasts, measured by ImageStreamX. One-tailed Wilcoxon matched-pairs signed-rank test; $^{*}p < 0.05$. **G,** SA-β-Gal distributions in UT (grey) or AraC (light blue) samples from either Sen High (right) or Sen Low patients (left). **H**, Volcano plot of DEGs in untreated Sen High versus Sen Low samples (*n*= 3 biologically independent samples); red, upregulated; green, underexpressed; black, not significant. **I,** GSEA normalized enrichment scores for significant Hallmark gene sets. **J**, PCA of batch-corrected normalized expression in six paired RNA-seq samples (UT, grey or AraC, light blue). **K**, *GLB1* expression shown as regularized log2 counts in UT and AraC-treated Sen Low and Sen High samples (*n*= 3 biologically independent samples). **L**, Representative western blot image of p21 (top) and γH2AX (bottom) protein levels in Sen Low and Sen High samples either UT or AraC treated. β-Actin and total H3 were used as loading control, respectively. **M**, Relative p21 (*n*= 5 independent experiments) and γH2AX (*n*= 3 independent experiments) levels, expressed as fold change over matched UT. Mean ± SEM. One-tailed Wilcoxon matched-pairs signed-rank test; $p < 0.05$. **N**, **O**, Contour (top) and ridge plots (bottom) showing cells expressing senescence signatures and module scores across clusters in the del(7) scRNA-seq dataset. Clusters are ordered by increasing mean score; the red dashed line marks the third quartile of the overall module-score distribution used to select cells with the highest signature expression.
Source data are provided as a Source Data file.

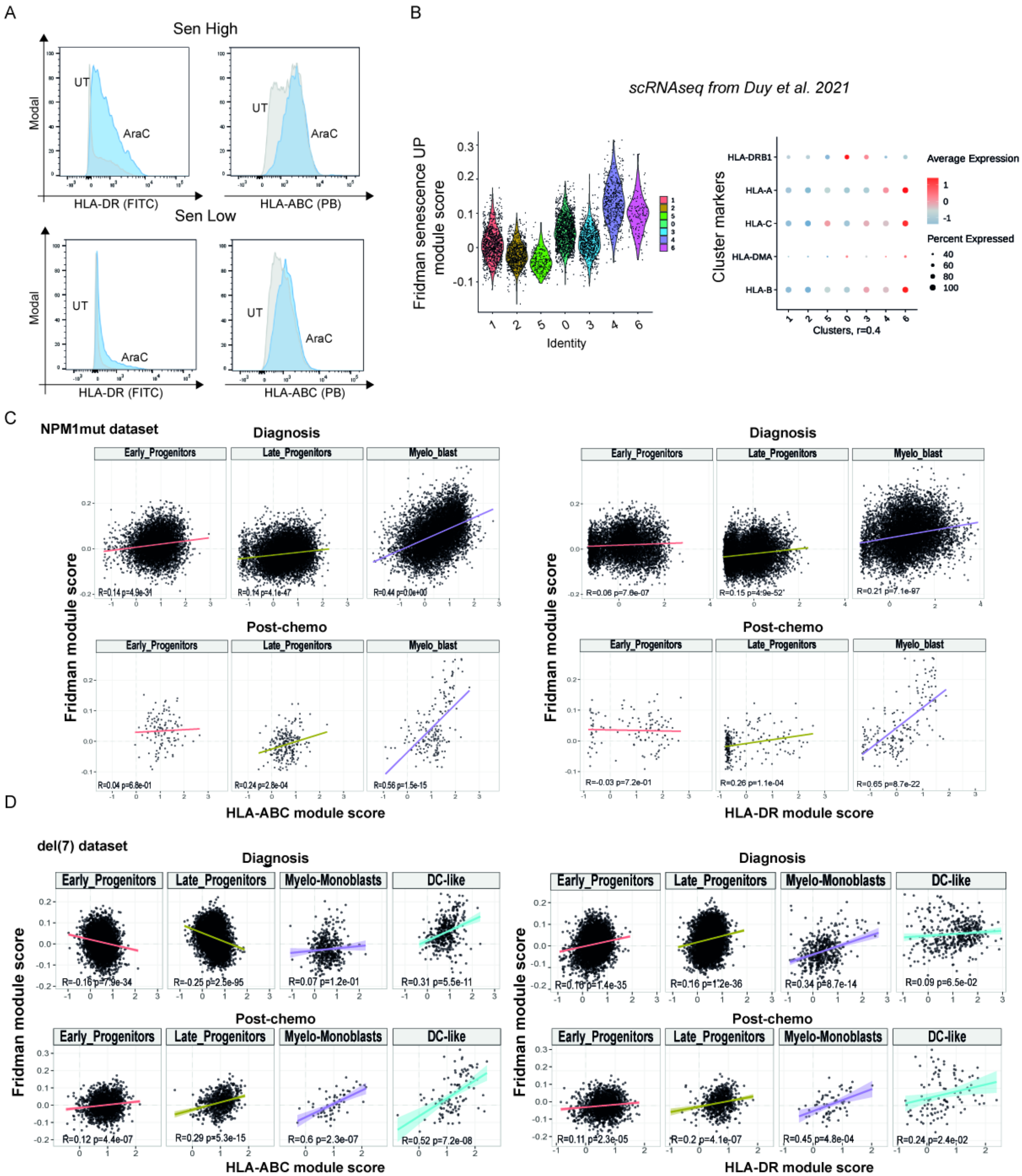
A
Sen High
Modal
UT
AraC
HLA-DR (FITC)
HLA-ABC (PB)
Sen Low
B
scRNAseq from Duy et al. 2021
Fridman senescence UP module score
Identity
Cluster markers
HLA-DRB1
HLA-A
HLA-C
HLA-DMA
HLA-B
Clusters, r=0.4
Average Expression
Percent Expressed
C
NPM1mut dataset
Diagnosis
Post-chemo
Early_Progenitors
Late_Progenitors
Myelo_blast
Fridman module score
R=0.14 p=4.9e-31
R=0.14 p=4.1e-47
R=0.44 p=0.0e+00
R=0.06 p=7.6e-07
R=0.15 p=4.9e-52
R=0.21 p=7.1e-97
R=0.04 p=6.8e-01
R=0.24 p=2.8e-04
R=0.56 p=1.5e-15
R=-0.03 p=7.2e-01
R=0.26 p=1.1e-04
R=0.65 p=8.7e-22
HLA-ABC module score
HLA-DR module score
D
del(7) dataset
Diagnosis
Post-chemo
Early_Progenitors
Late_Progenitors
Myelo-Monoblasts
DC-like
Fridman module score
R=-0.16 p=7.9e-34
R=-0.25 p=2.5e-95
R=0.07 p=1.2e-01
R=0.31 p=5.5e-11
R=0.16 p=1.4e-35
R=0.16 p=1.2e-36
R=0.34 p=8.7e-14
R=0.09 p=6.5e-02
R=0.12 p=4.4e-07
R=0.29 p=5.3e-15
R=0.6 p=2.3e-07
R=0.52 p=7.2e-08
R=0.11 p=2.3e-05
R=0.2 p=4.1e-07
R=0.45 p=4.8e-04
R=0.24 p=2.4e-02
HLA-ABC module score
HLA-DR module score

E

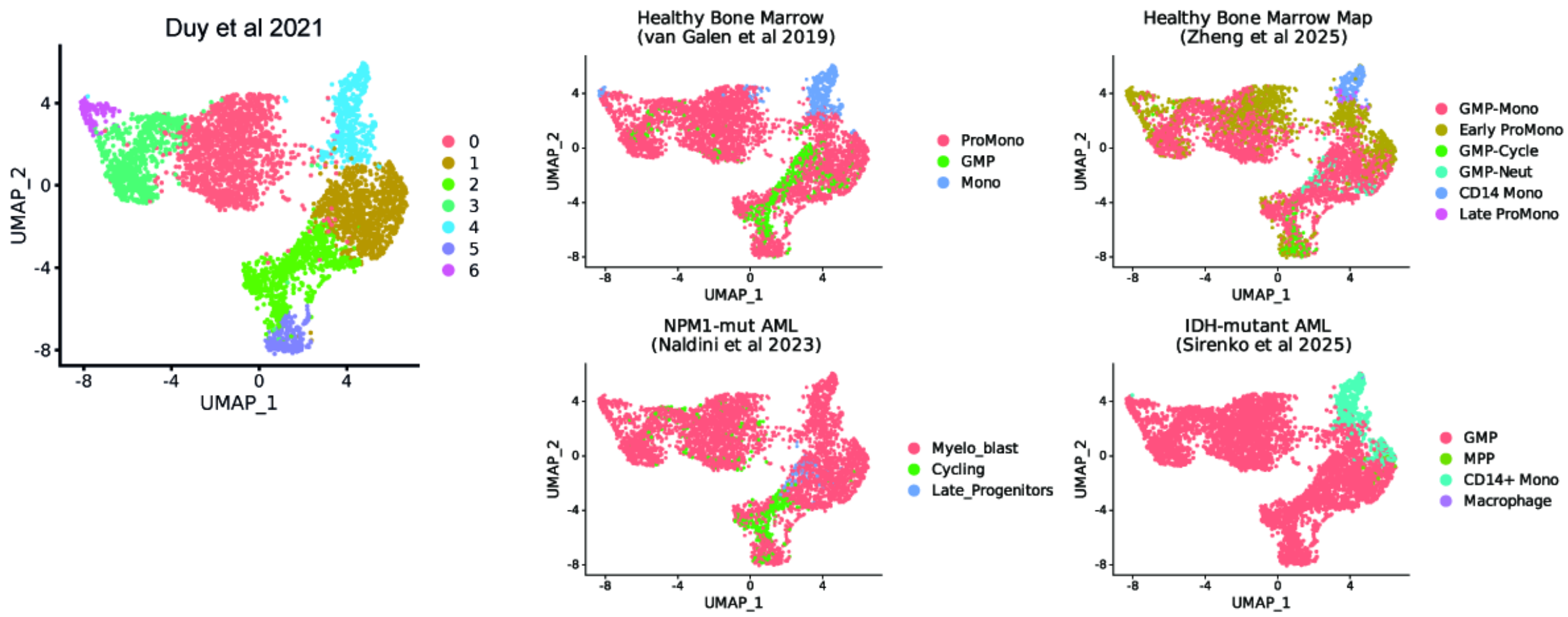

F

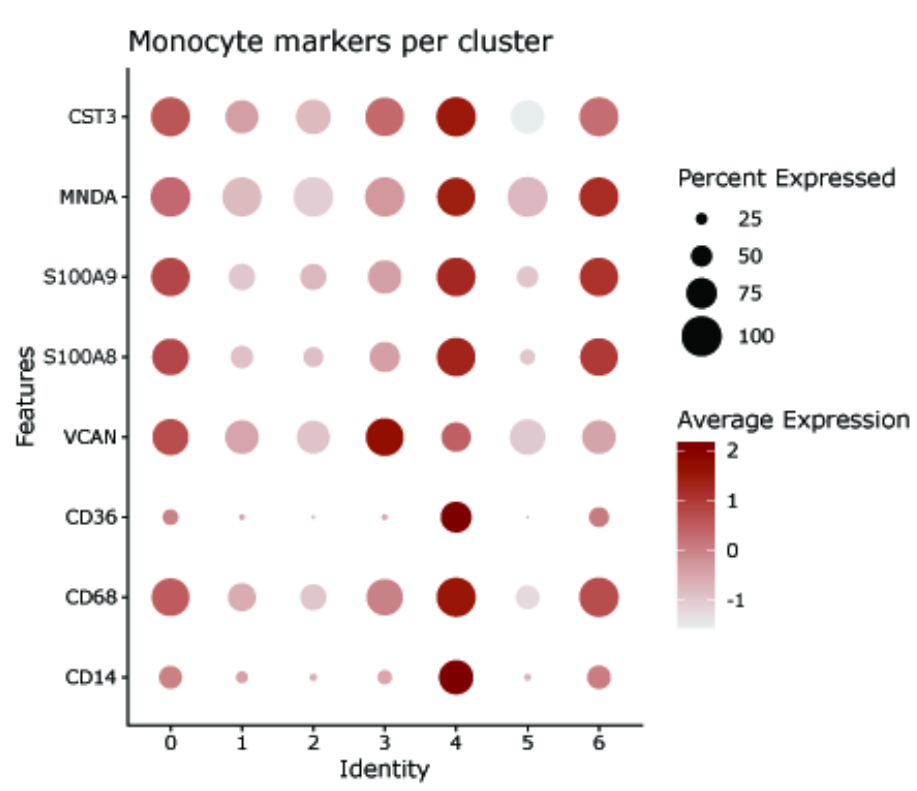

G

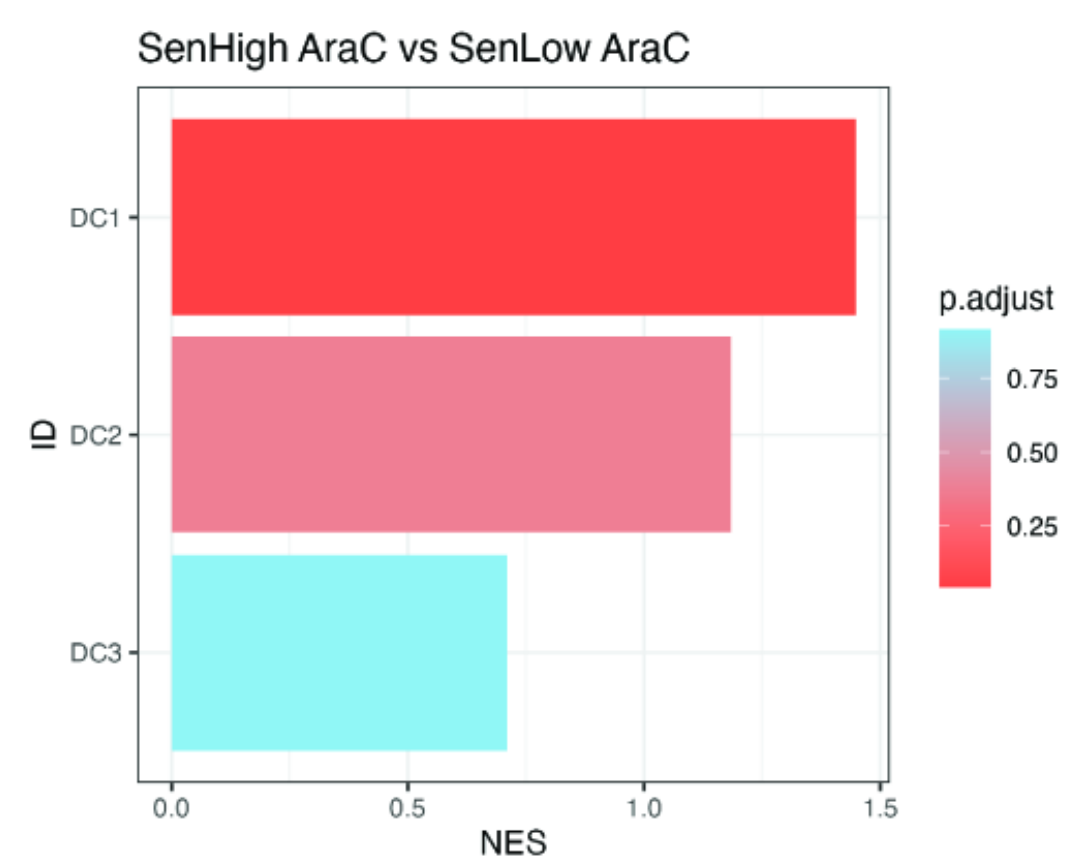

Fig. S2. TIS induces upregulation of HLA-class I and II and is associated with a monocyte-primed myeloid phenotype. A, Distribution of cells along the fluorescent intensity axis corresponding to HLA-DR (on the left) or HLA-ABC (on the right) expression. Two representative images with results from Sen Low (top) and Sen High (bottom) patients either untreated (UT, grey) or AraC treated (AraC, light blue) are shown. **B**, On the left: Violin plot showing the module score for genes belonging to the FRIDMAN_SENESCENCE_UP signature. On the right: Dot plot showing the average expression of HLA genes per cluster, the reported genes were significantly deregulated in the comparison AraC vs UT tested per cluster with the Wilcoxon Rank Sum test. **C, D**, Scatterplot showing the correlation between HLA class I and II and Friedman module score across AML subpopulations in NPM1^{mut} (C) and Del(7) (D) datasets. Linear regression lines are plotted for each cluster; Pearson correlation coefficient values and adjusted p-value are reported for each cluster on the bottom left of each figure. **E**, Reference mapping of AraC-treated clusters from the Duy *et al.* *ex-vivo* AML scRNA-seq dataset using multiple independent scRNA-seq

atlases, including healthy bone marrow and AML references. **F**, Dot plot showing expression of canonical monocytic markers across clusters in the Duy *et al.* dataset. Dot size indicates percentage of expressing cells; color intensity indicates average expression. **G**, Barplot showing Normalized Enrichment Score (NES) from GSEA. Genes were ranked by decreasing $\log_2$ (fold change), calculated from a differential gene expression analysis comparing Sen High vs Sen Low AML patients. The x-axis displays the NES for each gene set, the y-axis lists the gene sets associated with specific cell types. The color bar indicates the adjusted p-value of the enrichment.

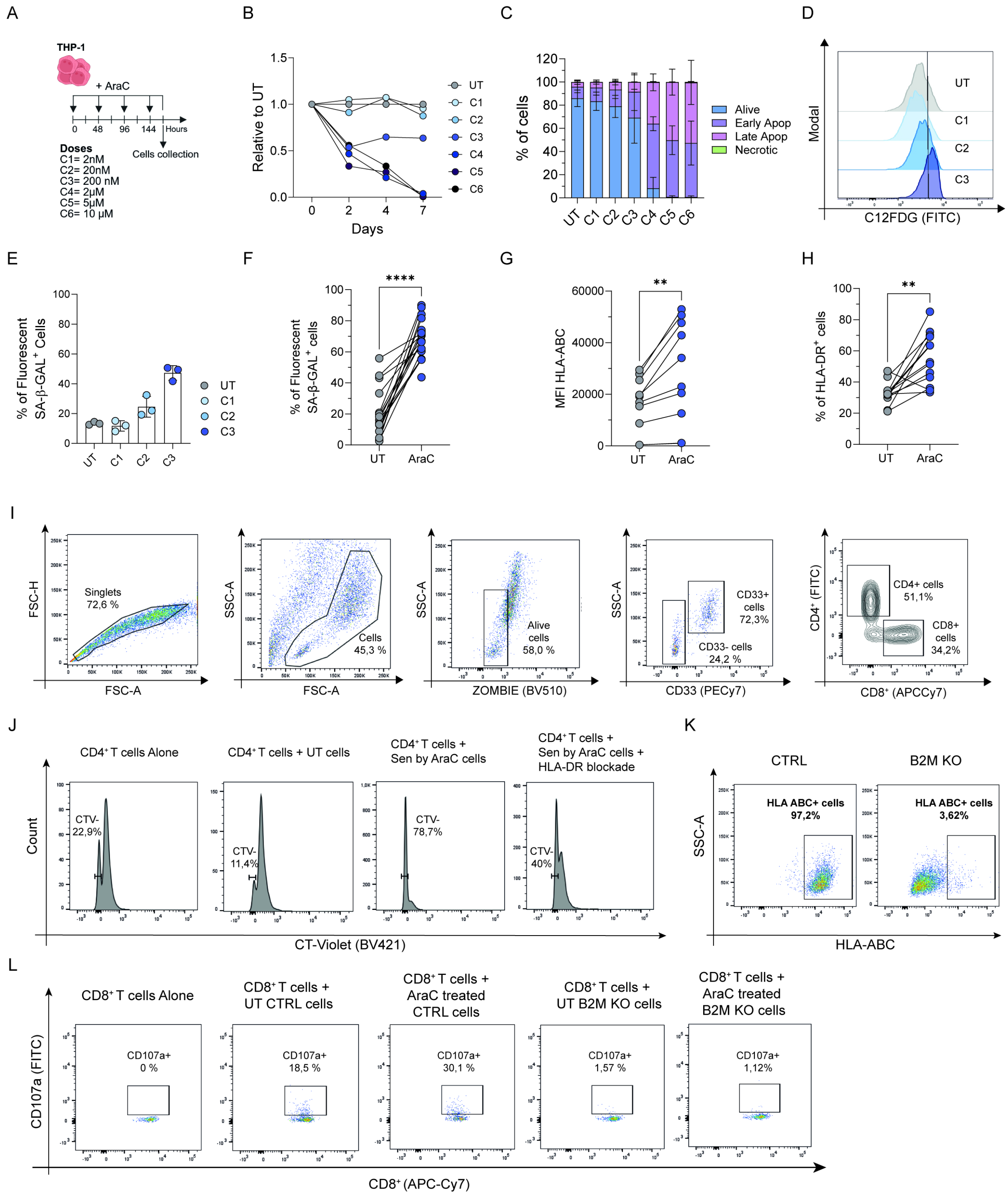

Fig. S3. Effects of TIS-induced T cell activation in co-culture with THP-1 cells. A, Schematic representation of AraC dose-response experiment; arrows indicate individual administrations and tested doses are shown. This figure was created in BioRender. **B**, AraC dose-response relative to UT THP-1 cells after 7 days, with cell counts on days 2, 4, and 7 (*n*= 5 technical replicates). **C**,

Apoptosis UT and AraC-treated THP-1 after 7 days of treatment. Alive: DAPI-/Annexin V-; Early apoptotic cells: DAPI-/Annexin V+; late apoptotic cells: DAPI+/Annexin V+; necrotic cells: DAPI+/Annexin V. Mean ± SD; *n*=8 technical replicates from three independent experiments. **D**, Representative C12FDG flow-cytometry profiles showing SA-β-Gal fluorescence in UT cells and cells treated with AraC C1 (2 nM), C2 (20 nM) or C3 (200 nM). **E**, Percentage of SA-β-Gal-positive cells under the conditions shown in D. Mean ± SD; *n*= 3 technical replicates. **F,** Percentage of SA-β-Gal-positive cells in UT (grey) and AraC-treated (light blue) THP-1 cells (*n*= 17 technical replicates across independent experiments). Two-tailed Wilcoxon matched-pairs signed-rank test; ****p <0.0001. **G** HLA-ABC median fluorescence intensity (MFI) in UT and AraC-treated THP-1 cells (*n*= 10 technical replicates across independent experiments). Two-tailed Wilcoxon matched-pairs signed-rank test; **p <0.01. **H,** Percentage of HLA-DR-positive cells in UT (*n*= 18) and AraC-treated (*n*= 16) THP-1 cells; dots indicate technical replicates across independent experiments. Two-tailed Wilcoxon matched-pairs signed-rank test; ***p <0.001. **H**, Percentage of HLA-DR positive cells from untreated (UT, grey; *n*= 18) and AraC (AraC, light blue; *n*= 16) treated AML cells are shown. Individual dots indicate technical replicates across different experiments. Statistical test: Two-tailed Wilcoxon matched pairs signed rank test; **p < 0.001. **I**, Representative sequential gating strategy for T cells. **J**, Representative gating strategy for T-cell proliferation after 5 days of co-culture: T cells alone or co-cultured with UT cells, AraC-induced senescent cells, or AraC-induced senescent cells with HLA-DR blockade. **K**, Representative HLA-ABC expression in electroporated/RNP-negative control (CTRL) and *B2M*-knockout cells. **L**, Representative gating strategy for $CD8^+$ T-cell degranulation after 7 days of co-culture with UT or AraC-treated CTRL and *B2M*-knockout cells.

Source data are provided as a Source Data file

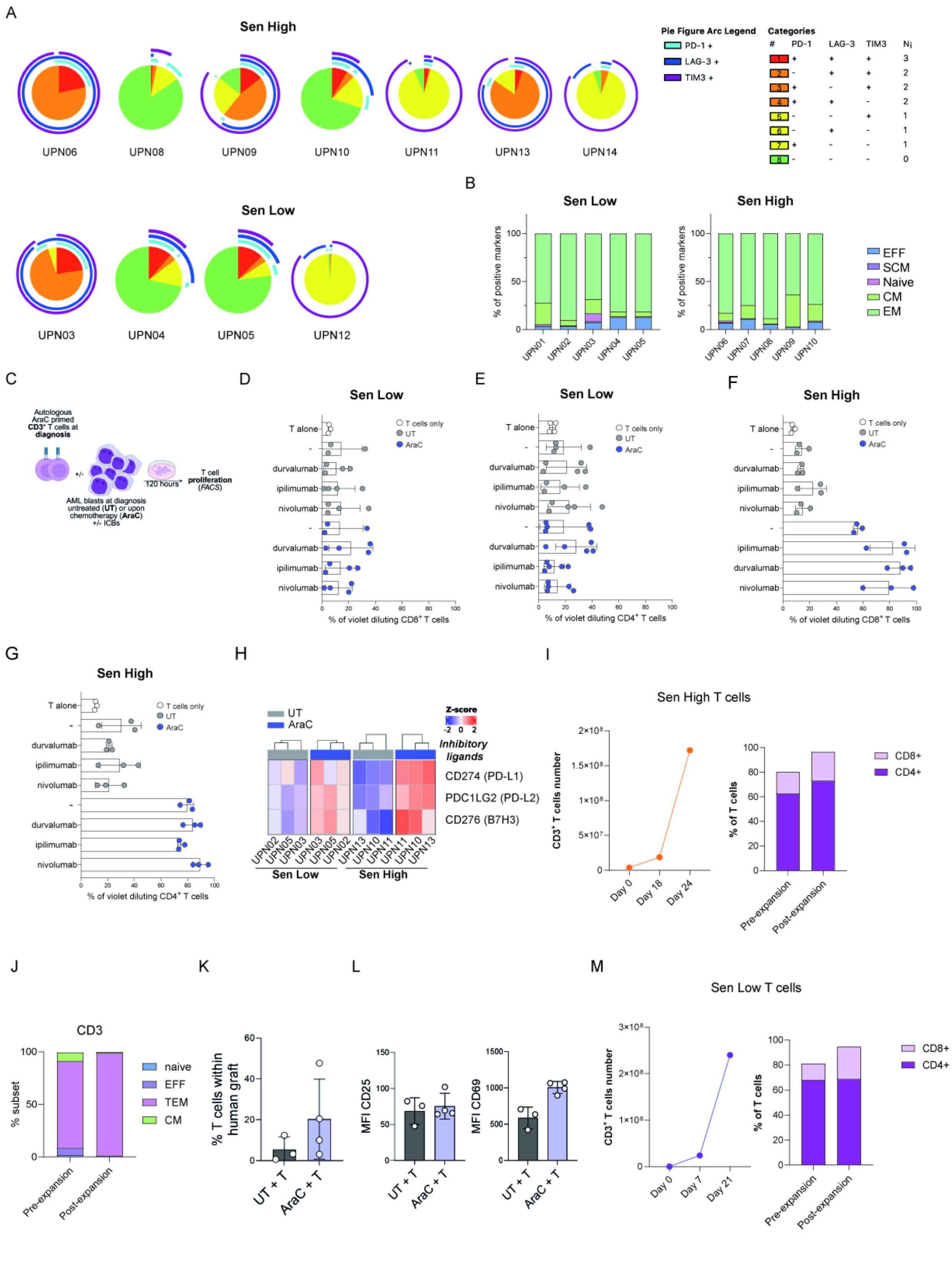

Fig. S4. T cell status and ICB response in Sen High and Sen Low AML. A, PD-1, LAG-3 and

TIM3 expression in T cells from Sen High (*n*= 7) and Sen Low (*n*= 4) samples. Pie charts: cells expressing 3, 2, 1 or 0 markers; arcs: positivity for PD-1 (light blue), LAG-3 (dark blue) and TIM3 (purple). **B**, T cell subset from Sen Low (left, *n*= 5) and Sen High (right, *n*= 5) samples. Stem cell memory: SCM; central memory: CM; effector memory: EM; terminal effector: EFF. **C**, MLR assay design with ICB treatment. This figure was created in BioRender. **D**, **E**, Proliferating $CD8^+$ (**D**) or $CD4^+$ (**E**) T cells, measured as of violet diluting cells upon co-colture with Sen Low blasts, UT (grey) or AraC-treated (light blue), ± ICBs. From top to bottom: *n* = 3, 3, 4, 4, 4, 3, 4, 4, 4 (**D**) and *n* = 4, 4, 5, 4, 5, 5, 5, 5, 5 (**E**). Mean ± SD. **F**, **G**, Proliferating $CD8^+$ (**F**) or $CD4^+$ (**G**) T cells, measured as violet diluting cells upon co-culture with Sen High blasts, UT (grey) or AraC-treated (light blue) ± ICBs. *N* = 3. Mean ± SD. **H**, Heat map of *PD-L1*, *PD-L2* and *B7H3* expression after AraC. **I**, Representative Sen High sample: left, $CD3^+$ T cell counts at days 0, 18 and 24; right, $CD4^+$ and $CD8^+$ percentages before and after expansion. **J**, T cell subset composition within $CD3^+$ T cells in a representative Sen High sample. **K**, T cells within the human $CD45^+$ graft in Sen High PDX mice receiving autologous T cells, ± AraC treatment. *N*= 3 mice per group. Mean ± SD. **L**, CD25 and CD69 MFI on T cells within the human $CD45^+$ graft (UT + T, *n*= 3 mice; AraC + T, *n*= 4 mice). Mean ± SD. **M**, Representative Sen Low sample: left, CD3+ T cell counts at days 0, 18 and 24; right, CD4+ and CD8+ percentages before and after expansion.

Unless otherwise indicated, *n* = biologically independent AML samples/individual mice. Source data are provided as a Source Data file.

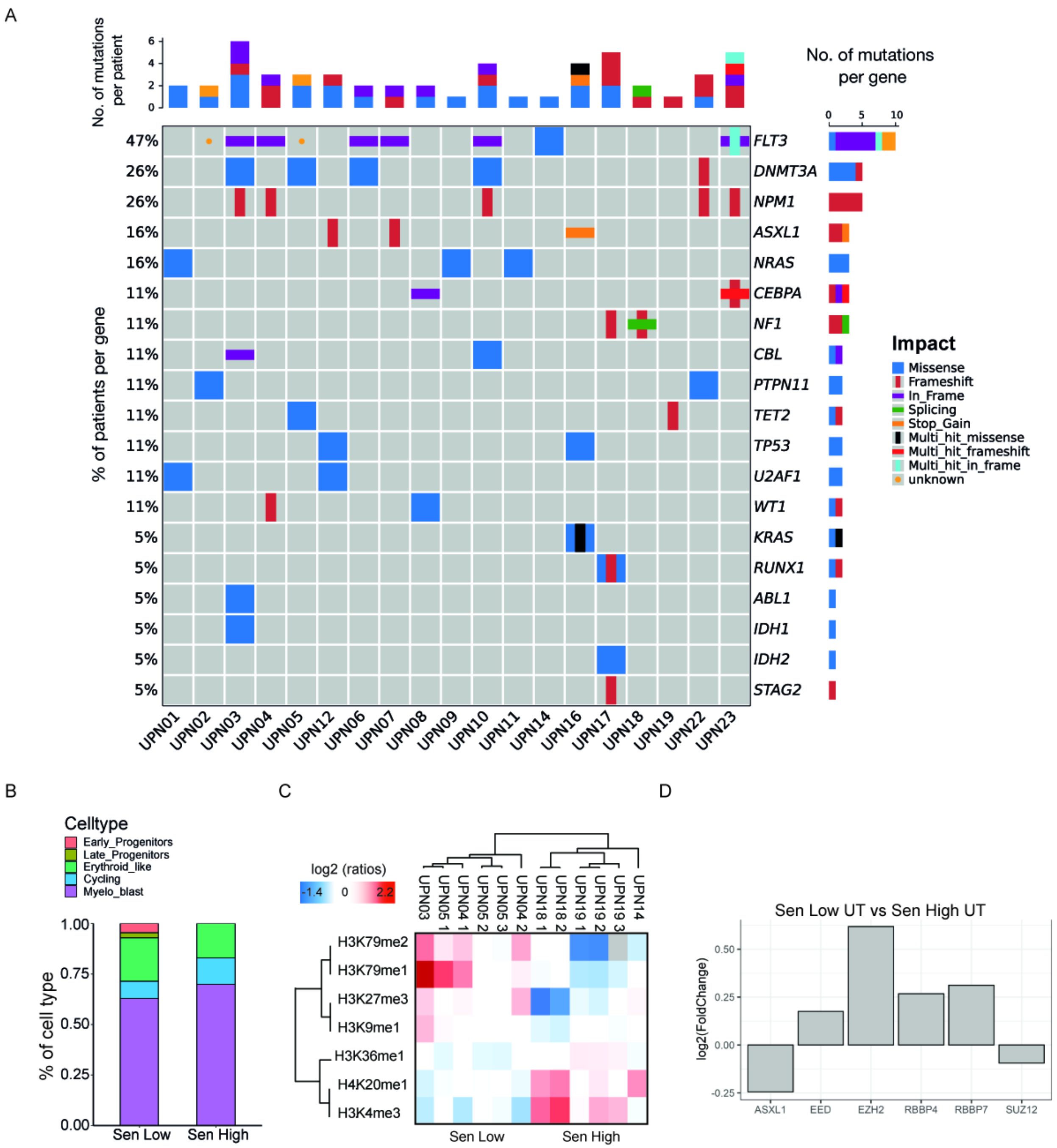

Fig. S5. Genetic, epigenetic and transcriptomic features of Sen Low and Sen High samples. **A,** Oncoplot depicting the genetic alterations in AML patients analyzed by NGS for a panel of leukemia-associated genes (NGS – QIAseq Targeted DNA Custom Panel (CDHS-33828Z-1177). Reference Genome: GRch37/hg19. Software analysis: CLC Genomics Workbench e QCI. Genes analyzed: *ABL1* (exons 4,5,6,7,8,9), *ANKRD26*(all exons), *ASXL1* (exons 9,11,12), *BRAF*(exon

15), *CALR* (exon 9), *CLB* (exons 8,9), *CDKN2A* (all exons), *CEBPA* (all exons), *CSF3R* (all exons), *DDX41* (all exons), *DNMT3A* (all exons), *ETNK1* (all exons), *ETV6* (all exons), *EZH2* (all exons), *FLT3* (13-15,20 exons), *GATA2* (2,3,4,5,6 exons), *IDH1* (exon 4), *IDH2* (exon 4), *HRAS* (exon 2,3), *IKZF1* (exon all), *JAK2* (exon all), *KIT* (exon 2,8-11,13,17,18), *KMT2A* (all exons), *KRAS* (exon 2,3), *MPL* (exon 10), *MYC* (all exons), *NF1* (all exons), *NOTCH1* (exon 26,27,28,34), *NPM1* (exon 10,11,12), *NRAS* (exon 2,3), *PTPN11* (exon 3,7-13), *RB1* (all exons), *RUNX1* (all exons), *SETBP1* (exon 4), *SF3B1* (exon 10-16), *SRSF2* (exon 1), *STAG2* (all exons), *TET2* (all exons), *TP53* (exon 2-11), *U2AF1* (exon 2,6), *WT1* (exon 6,7,8,9), *ZRSR2* (all exons). Highlighted are all oncogenic or potentially oncogenic alterations with variant allele frequency (VAF) higher than 3. Mutational load per patient (top), type of mutation (legend), and mutation frequency (left) are indicated. Sequenced AML samples based on DNA material availability: UPN01, UPN02, UPN03, UPN04, UPN05, UPN12 (Sen Low); UPN06, UPN07, UPN08, UPN09, UPN10, UPN11, UPN14, UPN16, UPN17, UPN18, UPN19, UPN22, UPN23 (Sen High). **B**, Estimated frequencies of cell type composition in our untreated samples using MuSiC deconvolution algorithm. **C**, Heatmap displaying a hierarchical clustering of L/H ratios (L: samples; H: internal standard) for the histone PTMs quantified by MS that showed significant changes between Sen Low and Sen High patient samples. The ratios were normalized to the average value across samples. Hierarchical clustering was performed using Pearson correlation and average linkage. Statistical test performed: T-test. *N*= 6 per group from 3 AML patients per group. **D**, Bar plot showing log2 (fold change) values (y-axis) for genes comprising the PRC2 complex, obtained by comparing gene expression between Sen Low untreated (UT) and Sen High untreated (UT) samples.

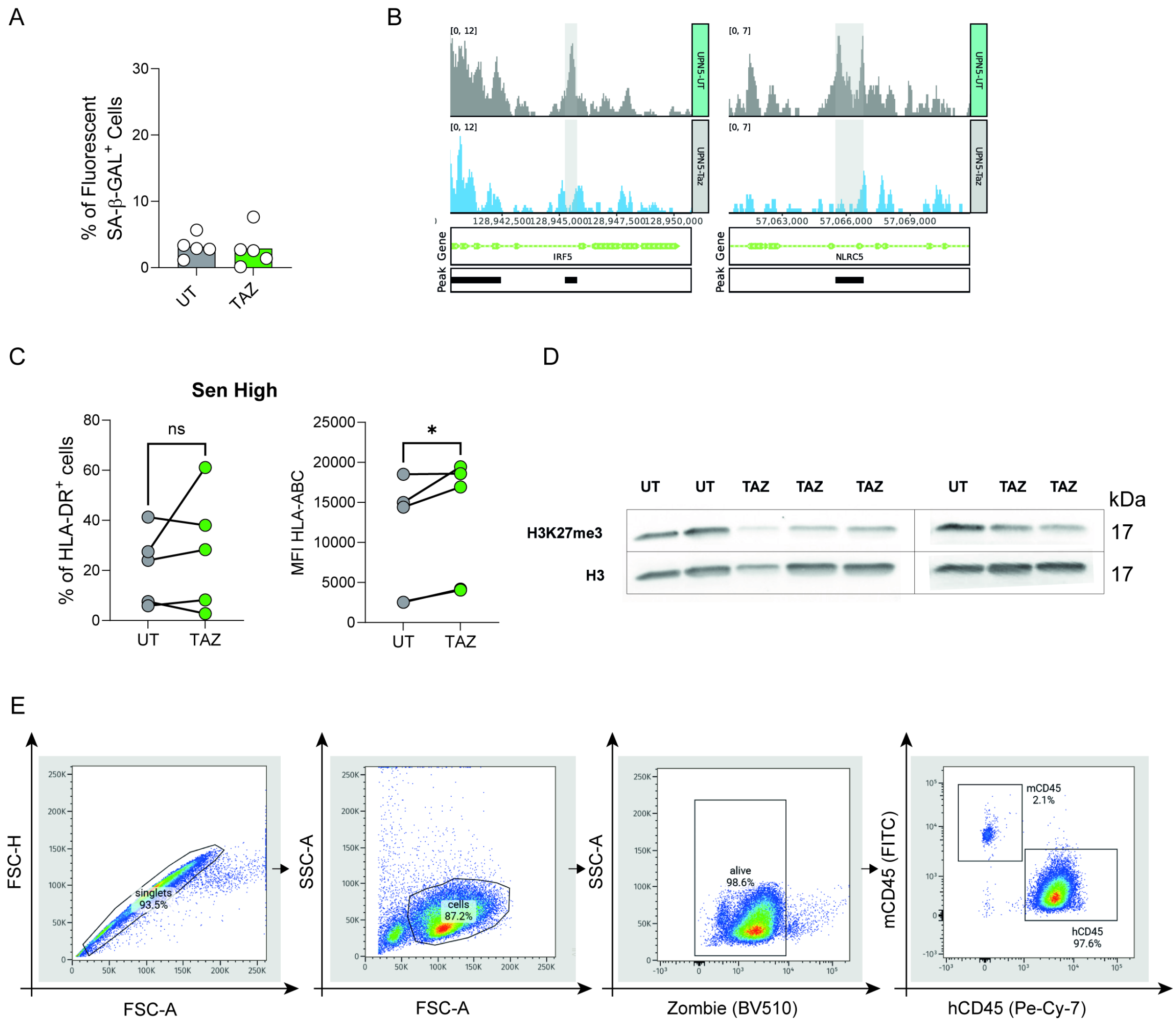

Fig. S6. Tazemetostat treatment effects on senescence and immunogenicity. A, Percentages of fluorescent SA-β-GAL positive cells in untreated (UT, greys dots) or Tazemetostat treated (TAZ, green dots) Sen Low AML samples (*n*= 5 independent experiments). **B**, Representative genome browser tracks of H3K27me3 CUT&Tag signal at the IRF5 and NLRC5 loci in Sen Low AML blasts, either untreated (UT) or treated with TAZ (Taz). Grey shaded areas highlight genomic regions showing reduced H3K27me3 occupancy upon TAZ treatment. Gene annotations and called peak regions are shown below each track. **C**, On the left, percentage of HLA-DR positive cells as detected by FACS analysis in untreated (UT, grey dots) or Tazemetostat treated (TAZ, green dots) Sen High AML samples (*n*= 5 independent experiments). Statistical test: One-tailed Wilcoxon matched pairs signed rank test. On the right, Median Fluorescence Intensity (MFI) of HLA-ABC as detected by FACS analysis in untreated (UT, grey dots) or Tazemetostat treated (TAZ, green dots) Sen High AML samples (*n*= 5 independent experiments). Statistical test:

One-tailed Wilcoxon matched pairs signed rank one-tailed test. ‘*’ $p < 0.05$. **D,** Representative western blot image of H3K27me3 levels measured in BM samples from mice treated with Tazemetostat (TAZ) or left untreated (UT). Total H3 was used as loading control. **E**, Representative gating strategy and flow cytometry plots showing the sequential gating workflow used for the PDX *in vivo* experiments.

Source data are provided as a Source Data file.

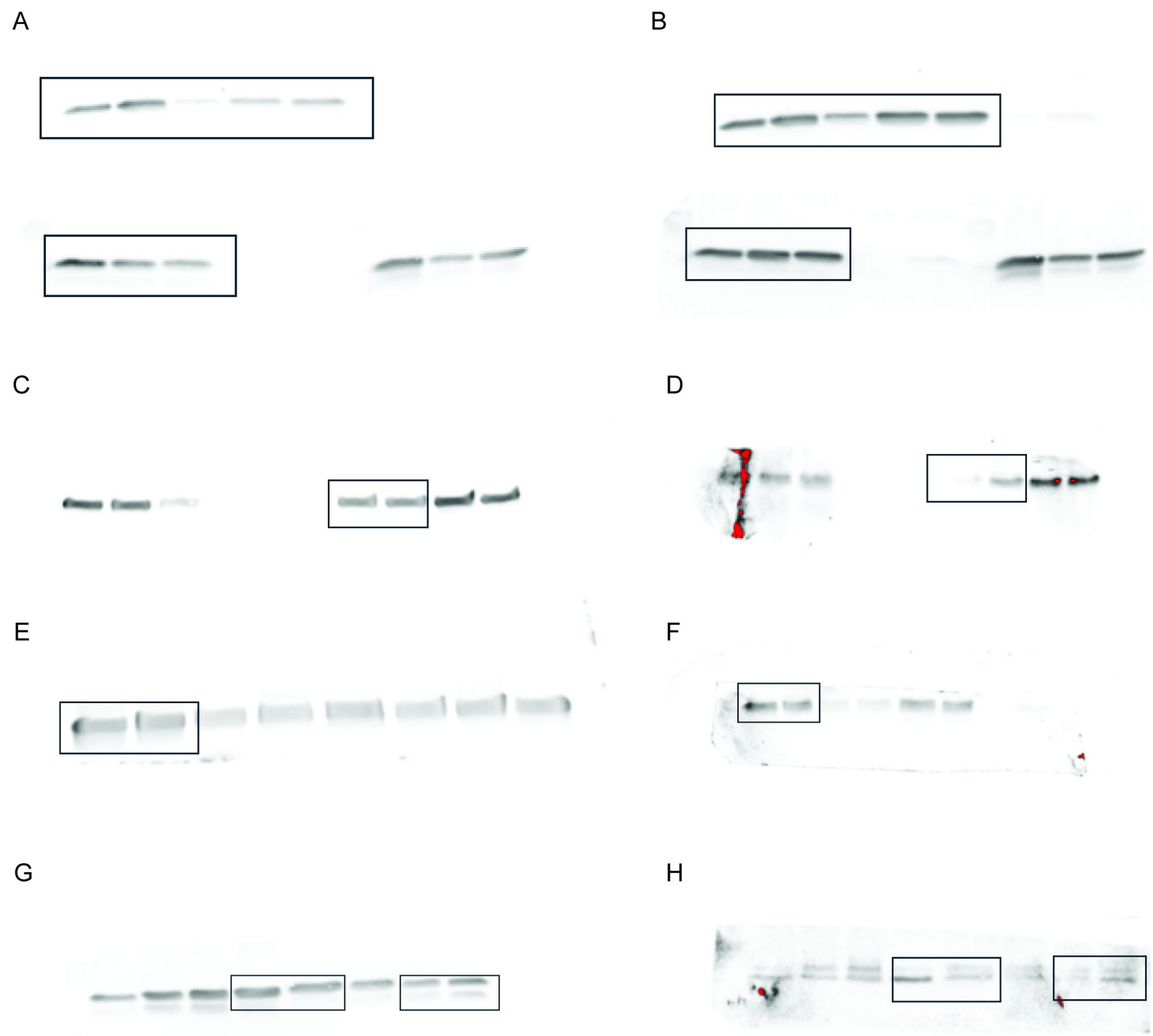

Uncropped western blot images:
A, Western blot image of H3K27me3 bands shown in Supplementary Fig. S6D. Excluded bands refer to *ex vivo* samples from another experiment. **B**, Western blot image of total H3 bands shown in Supplementary Fig. S6D. Excluded bands refer to *ex vivo* samples from another experiment. **C**, Western blot image of actin bands (Sen High sample) shown in Supplementary Fig. S1L. **D,** Western blot image of p21 bands (Sen High sample) shown in Supplementary Fig. S1L. **E**, Western blot image of actin bands (Sen Low sample) shown in Supplementary Fig. S1L. **F**, Western blot image of p21 bands (Sen Low sample) shown in Supplementary Fig. S1L. **G**, Western blot image of total H3 bands (Sen Low on the left, Sen High on the right) shown in Supplementary Fig. S1L. **H**, Western blot image of γH2AX bands (Sen Low on the left, Sen High on the right) shown in Supplementary Fig. S1L.

Representative western blot images with molecular marker. The letter of each panel corresponds to the uncropped image in the panel in figure above.

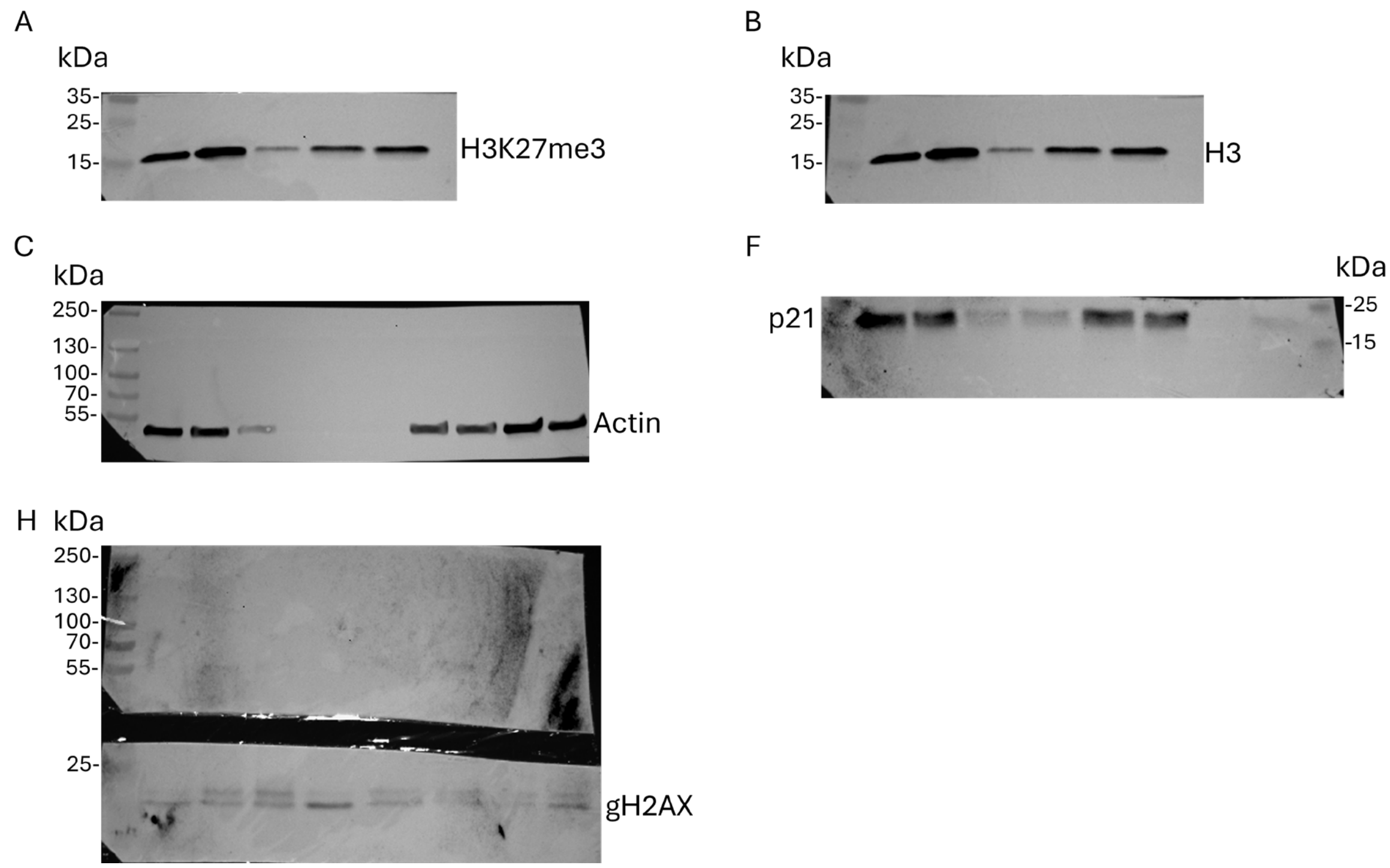

Supplementary Table 1. Detailed linear mixed-effects model output

For Figure 3B:

**LME model Raw outcome**	**Value**	**Std. Error**	**p-value**
**(Intercept)**	78.2474	8.1297	<0.0001
GroupT cells + AraC cells + HLA-DR blockade	-22.2409	6.0758	0.0012
GroupT cells + UT cells	-36.764	6.0136	<0.0001
GroupT cells + UT cells + HLA-DR blockade	-28.638	6.7554	3.0E-04
GroupT cells only	-56.3224	6.5605	<0.0001

**Contrast**	**Estimate**	**SE**	**p.value**
(T cells + AraC cells) - (T cells + AraC cells + HLA-DR blockade)	22.2409	6.0758	0.0118
(T cells + AraC cells) - (T cells + UT cells)	36.764	6.0136	<0.0001
(T cells + AraC cells) - (T cells + UT cells + HLA-DR blockade)	28.638	6.7554	0.0027
(T cells + AraC cells) - T cells alone	56.3224	6.5605	<0.0001
(T cells + AraC cells + HLA-DR blockade) - (T cells + UT cells)	14.5232	6.2114	0.2768
(T cells + AraC cells + HLA-DR blockade) - (T cells + UT cells + HLA-DR blockade)	6.3971	7.0338	1
(T cells + AraC cells + HLA-DR blockade) - T cells alone	34.0815	6.7721	3.0E-04
(T cells + UT cells) - (T cells + UT cells + HLA-DR blockade)	-8.1261	6.6627	1
(T cells + UT cells) - T cells alone	19.5583	6.3579	0.0502
(T cells + UT cells + HLA-DR blockade) - T cells only	27.6844	6.9129	0.0049

p-value adjustment: bonferroni method for 6 tests

For Figure 3D:

LME model Cubic root-transformed outcome	Value	Std. Error	p-value
**(Intercept)**	3.187	0.1819	<0.0001
GroupT cells + AraC cells + HLA-DR blockade	-0.6038	0.1491	5.00E-04
GroupT cells + UT cells	-0.7617	0.1437	<0.0001

Contrast	Estimate	SE	p.value
(T cells + AraC cells) - (T cells + AraC cells + HLA-DR blockade)	0.6038	0.1491	0.0016
(T cells + AraC cells) - (T cells + UT cells)	0.7617	0.1437	1.00E-04
(T cells + AraC cells + HLA-DR blockade) - (T cells + UT cells)	0.1579	0.1491	9.03E-01

p-value adjustment: bonferroni method for 3 tests

For Figure 3F:

LME model Square-root transformed outcome	Value	Std. Error	p-value
**(Intercept)**	2.5187	0.6761	0.001
GroupT cells + AraC B2M KO cells	0.7982	0.6082	0.2013
GroupT cells + AraC cells	2.622	0.5516	1.00E-04
GroupT cells + B2M KO cells	1.0365	0.6082	0.1008
GroupT cells + UT cells	1.5034	0.5516	0.0115

Contrast	Estimate	SE	p.value
T cells only - (T cells + AraC B2M KO cells)	-0.7982	0.6082	1
T cells only - (T cells + AraC cells)	-2.622	0.5516	7.00E-04
T cells only - (T cells + B2M KO cells)	-1.0365	0.6082	1
T cells only - (T cells + UT cells)	-1.5034	0.5516	0.1155
(T cells + AraC B2M KO cells) - (T cells + AraC cells)	-1.8238	0.5586	0.0317

(T cells + AraC B2M KO cells) - (T cells + B2M KO cells)	-0.2383	0.5977	1
(T cells + AraC B2M KO cells) - (T cells + UT cells)	-0.7052	0.5586	1
(T cells + AraC cells) - (T cells + B2M KO cells)	1.5855	0.5586	0.0887
(T cells + AraC cells) - (T cells + UT cells)	1.1187	0.488	0.3058
(T cells + B2M KO cells) - (T cells + UT cells)	-0.4669	0.5586	1

p-value adjustment: bonferroni method for 10 tests

For Figure 3G:

**LME model Ordernorm-transformed outcome**	**Value**	**Std. Error**	**p-value**
**(Intercept)**	-1.0921	0.1904	<0.0001
GroupT cells + AraC cells	1.9913	0.2362	<0.0001
GroupT cells + UT cells	1.1746	0.2441	1.00E-04

**Contrast**	**Estimate**	**SE**	**p.value**
T cells only - (T cells + AraC cells)	-1.9913	0.2362	<0.0001
T cells only - (T cells + UT cells)	-1.1746	0.2441	4.00E-04
(T cells + AraC cells) - (T cells + UT cells)	0.8168	0.2441	0.0108

p-value adjustment: bonferroni method for 3 tests

For Figure 4B:

**LME model Cubicroot-transformed outcome**	**Value**	**Std. Error**	**p-value**
**(Intercept)**	2.0881	0.2474	<0.0001
GroupT cells only + AraC cells	1.3894	0.2333	<0.0001
GroupT cells only + UT cells	0.6116	0.2333	0.0211

**Contrast**	**Estimate**	**SE**	**p.value**

T cells only - (T cells only + AraC cells)	-1.3894	0.2333	1.00E-04
T cells only - (T cells alone + UT cells)	-0.6116	0.2333	0.0634
(T cells only + AraC cells) - (T cells only + UT cells)	0.7778	0.2333	0.0162

p-value adjustment: bonferroni method for 3 tests

For Figure 4C:

LME Log-transformed outcome	Value	Std. Error	p-value
**(Intercept)**	1.7528	0.2017	<0.0001
GroupT cells only + AraC cells	1.6157	0.2427	<0.0001
GroupT cells only + UT cells	0.5695	0.2427	0.0355

Contrast	Estimate	SE	p.value
T cells only - (T cells only + AraC cells)	-1.6157	0.2427	<0.0001
T cells only - (T cells only + UT cells)	-0.5695	0.2427	0.1065
(T cells only + AraC cells) - (T cells only + UT cells)	1.0462	0.2427	0.0025

p-value adjustment: bonferroni method for 3 tests.

For Figure 4G:

LME ordenorm transformed outcome	Value	Std. Error	p-value
**(Intercept)**	0.8007	0.1991	0.001
GroupAraC	-1.5286	0.2751	<0.0001

For Figure 4H:

LME ordenorm transformed outcome	Value	Std. Error	p-value
**(Intercept)**	-0.5035	0.5118	0.3398
GroupAraC	0.6634	0.2373	0.013

For Figure 4I:

LME ordenorm transformed outcome	Value	Std. Error	p-value

**(Intercept)**	-0.2314	0.5995	0.7058
GroupAraC	0.4894	0.1949	0.026

For Figure 4J:

**LME model log transformed outcome**	**Value**	**Std. Error**	**p-value**
**(Intercept)**	15.9175	0.4847	<0.0001
GroupAraC	-2.627	0.5948	1e-04
GroupAraC + T cells	-3.322	0.48	<0.0001
GroupAraC + T cells + Niv	-4.7994	0.6016	<0.0001
GroupUT + T cells	0.0481	0.5114	0.9257

**Contrast**	**Estimate**	**SE**	**p.value**
UT - AraC	2.627	0.5948	0.0013
UT - (AraC + T cells)	3.322	0.48	<0.0001
UT - (AraC + T cells + Niv)	4.7994	0.6016	<0.0001
UT - (UT + T cells)	-0.0481	0.5114	1
AraC - (AraC + T cells)	0.695	0.5627	1
AraC - (AraC + T cells + Niv)	2.1724	0.7141	0.0495
AraC - (UT + T cells)	-2.6751	0.5754	7e-04
(AraC + T cells) - (AraC + T cells + Niv)	1.4774	0.5627	0.1367
(AraC + T cells) - (UT + T cells)	-3.3701	0.4669	<0.0001
(AraC + T cells + Niv) - (UT + T cells)	-4.8475	0.5999	<0.0001

p-value adjustment: bonferroni method for 10 tests
