# Supplementary material for: Therapy induced senescence promotes immunogenicity in acute myeloid Leukemia through reduced EZH2 activity
Source: Nat Commun. 2026 Sep 4;17:9151. doi: 10.1038/s41467-026-76853-1 (PMC13545235; doi:10.1038/s41467-026-76853-1)
Supplement: Supplementary file 2 [file 41467_2026_76853_MOESM2_ESM.pdf]

Description of Additional Supplementary Files

Supplementary Data 1: Comprehensive characterization of AML patients.

Supplementary Data 2: RNA-seq differential expression and GSEA analyses.

The Excel file contains five worksheets: 2.1, differential expression analysis comparing untreated Sen High and Sen Low samples; 2.2, differential expression analysis based on the interaction model; 2.3, GSEA results for Hallmark gene sets from the MSigDB database based on the interaction analysis; 2.4, GSEA results for senescence signatures based on the interaction analysis; 2.5, differential expression analysis using the multimapping strategy for HLA-related analyses.

Supplementary Data 3: Single-cell pseudobulk differential expression analysis comparing AraC-treated and untreated cells from the Duy et al. dataset.

Supplementary Data 4: HLA-I-bound peptides identified in Sen Low and Sen High samples.

Supplementary Data 5: Gene sets used for GSEA and differential expression results for the SenHighAraC versus SenLowAraC comparison.
