# Supplementary material for: Therapy induced senescence promotes immunogenicity in acute myeloid Leukemia through reduced EZH2 activity
Source: Nat Commun. 2026 Sep 4;17:9151. doi: 10.1038/s41467-026-76853-1 (PMC13545235; doi:10.1038/s41467-026-76853-1)
Supplement: Supplementary file 3 [file 41467_2026_76853_MOESM8_ESM.pdf]

nature portfolio

Corresponding author(s): Di Micco Raffaella

Last updated by author(s): 23/07/2026

Reporting Summary

Nature Portfolio wishes to improve the reproducibility of the work that we publish. This form provides structure for consistency and transparency in reporting. For further information on Nature Portfolio policies, see our Editorial Policies and the Editorial Policy Checklist.

Statistics

For all statistical analyses, confirm that the following items are present in the figure legend, table legend, main text, or Methods section.

n/a	Confirmed	
☐	☒	The exact sample size (*n*) for each experimental group/condition, given as a discrete number and unit of measurement
☐	☒	A statement on whether measurements were taken from distinct samples or whether the same sample was measured repeatedly
☐	☒	The statistical test(s) used AND whether they are one- or two-sided<br>*Only common tests should be described solely by name; describe more complex techniques in the Methods section.*
☐	☒	A description of all covariates tested
☐	☒	A description of any assumptions or corrections, such as tests of normality and adjustment for multiple comparisons
☐	☒	A full description of the statistical parameters including central tendency (e.g. means) or other basic estimates (e.g. regression coefficient) AND variation (e.g. standard deviation) or associated estimates of uncertainty (e.g. confidence intervals)
☐	☒	For null hypothesis testing, the test statistic (e.g. *F*, *t*, *r*) with confidence intervals, effect sizes, degrees of freedom and *P* value noted<br>*Give P values as exact values whenever suitable.*
☒	☐	For Bayesian analysis, information on the choice of priors and Markov chain Monte Carlo settings
☒	☐	For hierarchical and complex designs, identification of the appropriate level for tests and full reporting of outcomes
☐	☒	Estimates of effect sizes (e.g. Cohen's *d*, Pearson's *r*), indicating how they were calculated

Our web collection on statistics for biologists contains articles on many of the points above.

Software and code

Policy information about availability of computer code

Data collection	Flow cytometry data were acquired on a FACSCanto II or FACSymphony A5 SORP and cell sorting was performed on a FACSAria Fusion using BD FACSDiva v[X]. Image-based flow cytometry data were acquired on an ImageStreamX instrument using [software, version]. RNA quantity and quality were assessed using a Qubit 2.0 fluorometer and an Agilent 4200 TapeStation, respectively. RNA sequencing was performed on a NovaSeq instrument in a 2 × 150-bp configuration. Immunopeptidomics data were acquired using a nanoElute LC system coupled to a timsTOF SCP mass spectrometer using [software, version]. Histone PTM mass spectrometry data were acquired on a Q Exactive Plus using Xcalibur.
Data analysis	Flow cytometry data were analyzed using FlowJo v10.10.0 (Tree Star/BD Life Sciences). Statistical analyses were performed using GraphPad Prism v10 (GraphPad Software) and R v4.4.1. Bulk RNA-seq data were processed using STAR v2.7.6a, featureCounts v2.0.1, DESeq2 v1.50.2 and clusterProfiler v4.18.4. Hallmark gene sets were obtained from MSigDB v7.2. Single-cell RNA-seq differential expression and module-score analyses were performed using Seurat v3.2.2, whereas reference-mapping analyses were performed using Seurat v5.2.0. Bulk RNA-seq deconvolution was performed using MuSiC v1.0.0. Immunopeptidomics data were analyzed using PEAKS Studio v11.0 (build 20230414), including the PEAKS DeepNovo algorithm, and peptide–HLA binding affinity was predicted using NetMHC v4.0. Histone PTM mass spectrometry data were analyzed using EpiProfile v2.0. Peptide annotation and enrichment analyses were performed using queryup v1.0.5, clusterProfiler v4.18.4 and ReactomePA v1.34.0. CUT&Tag data were processed using Trim Galore v0.6.10, Bowtie2 v2.5.4, Picard MarkDuplicates v3.1.1, MACS2 v2.2.9.1, bedtools v2.31.1, ChIPseeker v1.38.0, ggcoverage v1.4.1 and deepTools v3.5.5. Custom scripts and functions were used for the interaction-direction analysis and are available through the study code repository.

For manuscripts utilizing custom algorithms or software that are central to the research but not yet described in published literature, software must be made available to editors and reviewers. We strongly encourage code deposition in a community repository (e.g. GitHub). See the Nature Portfolio guidelines for submitting code & software for further information.

Data

Policy information about availability of data

All manuscripts must include a data availability statement. This statement should provide the following information, where applicable:

- Accession codes, unique identifiers, or web links for publicly available datasets
- A description of any restrictions on data availability
- For clinical datasets or third party data, please ensure that the statement adheres to our policy

The sequencing datasets generated in this study have been deposited in the NCBI Gene Expression Omnibus under SuperSeries accession GSE324154 (https://www.ncbi.nlm.nih.gov/geo/query/acc.cgi?acc=GSE324154), including RNA-seq data from AraC-treated samples under accession GSE229736 (https://www.ncbi.nlm.nih.gov/geo/query/acc.cgi?acc=GSE229736) and RNA-seq data from tazemetostat-treated samples under accession GSE324153 (https://www.ncbi.nlm.nih.gov/geo/query/acc.cgi?acc=GSE324153). CUT&Tag data have been deposited under accession GSE336442 (https://www.ncbi.nlm.nih.gov/geo/query/acc.cgi?acc=GSE336442). Histone PTM mass spectrometry data have been deposited in the ProteomeXchange Consortium via the PRIDE repository under accession PXD065846 (https://www.ebi.ac.uk/pride/archive/projects/PXD065846), and LC–MS immunopeptidomics data under accession PXD068731 (https://www.ebi.ac.uk/pride/archive/projects/PXD068731). Anonymized participant-level metadata are provided in Supplementary Data 1, and Source Data are provided with the paper.
The code used to generate the results reported in this study is publicly available at http://www.bioinfotiget.it/gitlab/custom/aml_gilioli_fusco_2026

Research involving human participants, their data, or biological material

Policy information about studies with human participants or human data. See also policy information about sex, gender (identity/presentation), and sexual orientation and race, ethnicity and racism.

Reporting on sex and gender	We worked on patient-derived AML primary cells from both female and male individuals. Participant sex was obtained from clinical records. Both female and male participants were included, and sex was not used as an inclusion or exclusion criterion. Sex-stratified analyses were not performed because the individual experimental cohorts were small and the study was not designed or powered to assess sex-specific effects. Gender identity was not collected.
Reporting on race, ethnicity, or other socially relevant groupings	Race and ethnicity data were not collected and were therefore not considered in the study design or analyzed.
Population characteristics	Experiments were performed using primary AML cells derived from bone marrow samples collected at diagnosis from 21 participants aged 30–76 years. Participant-level clinical characteristics are reported in Supplementary Data 1.number of patients 21). Participant-level age, sex and clinical characteristics are reported in Supplementary Data 1.
Recruitment	Primary AML samples were obtained from the San Raffaele Leukemia Biobank from participants who had provided written informed consent. No participants were recruited specifically for this study. Samples were selected based on their availability and suitability for the planned experiments, which may have introduced selection bias related to sample availability. Participants received no financial compensation for sample collection.
Ethics oversight	The Leukemia Biobank protocol and informed-consent documentation were approved by the San Raffaele Ethics Committee, IRCCS Ospedale San Raffaele, Milan, Italy, on 12 July 2010, with the latest amendment approved on 14 June 2012. Patient-derived samples were analyzed under the SEN-LEUK protocol, approved by the same committee on 10 December 2017, with the latest amendment approved on 14 March 2024.

Note that full information on the approval of the study protocol must also be provided in the manuscript.

Field-specific reporting

Please select the one below that is the best fit for your research. If you are not sure, read the appropriate sections before making your selection.

☒ Life sciences ☐ Behavioural & social sciences ☐ Ecological, evolutionary & environmental sciences

For a reference copy of the document with all sections, see nature.com/documents/nr-reporting-summary-flat.pdf

Life sciences study design

All studies must disclose on these points even when the disclosure is negative.

Sample size	Sample sizes were not determined using formal a priori statistical power calculations. For experiments using primary AML specimens, sample sizes were determined by the availability of clinically annotated samples with sufficient viability and cell numbers for the planned analyses, while maximizing the number of biologically independent patient samples and using paired untreated and treated conditions whenever possible. This paired design increased sensitivity to treatment-associated changes while accounting for inter-patient variability. For cell-line experiments, sample sizes were selected based on independent experimental repeats and technical replicates required to assess assay reproducibility, as detailed in the corresponding figure legends. For omics analyses, sample sizes were additionally constrained by the amount and quality of primary material required for each assay. Immunopeptidomics and histone PTM analyses each included three biologically independent Sen High and three Sen Low AML samples; for histone PTM analysis, each patient sample was analyzed in two independent experiments performed on separate days. For in vivo PDX studies, group sizes were determined by the number of successfully engrafted mice

	available and by the requirement to balance pre-treatment leukemia burden across experimental groups, while limiting animal use. Exact sample sizes are reported in the corresponding figure legends and in Supplementary Table 1. Inferential statistical analyses were performed only when permitted by the sample size and experimental design; non-parametric tests were applied when at least three biologically independent observations were available, whereas linear mixed-effects models were fitted only for datasets containing at least five biologically independent observations.
Data exclusions	No data were excluded from the analysis.
Replication	Experiments were performed using biologically independent patient samples, independent cell-culture experiments or individual mice, as specified in the corresponding figure legends. Most in vitro experiments included at least three biological replicates. Exceptions, including experiments limited by the availability of primary AML material and selected PDX studies performed as a single experimental cohort with the indicated number of mice per group, are explicitly reported in the figure legends. Technical replicates were not considered biologically independent observations. Where independent experimental replication was performed, comparable results were obtained.
Randomization	Primary AML samples were not randomly allocated to the Sen High and Sen Low groups, as these groups were defined according to a prespecified biological response to AraC treatment. For in vitro experiments, aliquots from the same patient sample or cell preparation were assigned to the relevant untreated and treatment conditions, using matched or paired designs whenever possible to control for inter-patient variability. Established cell-line cultures were allocated to experimental conditions according to the predefined study design. Participants were not prospectively assigned to experimental groups, as the study used biobanked primary AML samples selected according to sample availability, viability and suitability for the planned analyses. For PDX experiments, mice with confirmed engraftment were allocated to treatment groups while balancing pretreatment AML burden, assessed by bone-marrow aspirate, across groups.
Blinding	Investigators were not blinded to sample identity or treatment allocation during in vitro experiments, flow-cytometry acquisition or in vivo treatment and monitoring. For flow-cytometry analyses, gating strategies and thresholds were predefined using appropriate negative, unstained or untreated controls and were applied uniformly across samples. Quantification was based on objective software-derived measurements, including percentages of positive cells and median fluorescence intensity. Investigators were not blinded during PDX dosing and monitoring because of practical requirements associated with treatment administration. Bioinformatic and statistical analyses were performed using predefined pipelines and analysis criteria that were applied consistently across groups. These measures were used to minimize potential observer bias.

Reporting for specific materials, systems and methods

We require information from authors about some types of materials, experimental systems and methods used in many studies. Here, indicate whether each material, system or method listed is relevant to your study. If you are not sure if a list item applies to your research, read the appropriate section before selecting a response.

Materials & experimental systems

n/a	Involved in the study	
☐	☒	Antibodies
☐	☒	Eukaryotic cell lines
☒	☐	Palaeontology and archaeology
☐	☒	Animals and other organisms
☒	☐	Clinical data
☒	☐	Dual use research of concern
☒	☐	Plants

Methods

n/a	Involved in the study	
☒	☐	ChIP-seq
☐	☒	Flow cytometry
☒	☐	MRI-based neuroimaging

Antibodies

Antibodies used	*HLA-ABC Pacific Blue (Clone W6/32, cat#311418, BioLegend); 1:100 dilution<br>*HLA-DR FITC (Clone L243, cat#307604, BioLegend); 1:100 dilution<br>*HLA-DR APC-Cy 7 (cat#307618, BioLegend); 1:100 dilution<br>*CD4 FITC (cat#563550, BioLegend); 1:100 dilution<br>*CD8 APCH7 (cat#641400, BD); 1:100 dilution<br>*CD69 APC (cat#310910, BioLegend); 1:100 dilution<br>*CD25 PE (cat#130-113-286, Miltenyi Biotec); 1:100 dilution<br>*CD33 (cat# 333952, BD Biosciences); 1:100 dilution<br>*CD8 APC-Cy7 (cat# 344714, Biolegend); 1:100 dilution<br>*CD4 FITC (cat#980802, BioLegend); 1:100 dilution<br>*CD4 PE (cat#300508, BioLegend); 1:100 dilution<br>*CD69 APC (cat#310910, BioLegend); 1:100 dilution<br>*CD25 PE (cat#130-113-286, Miltenyi Biotec); 1:100 dilution<br>*ZOMBIE Aqua (cat#423102, BioLegend); 1:400 dilution<br>*TIM3 APC (cat#345011, BioLegend); 1:100 dilution<br>*LAG-3 Brilliant Violet 605 (cat#369324, BioLegend); 1:100 dilution<br>*PD-1 PE (#cat329706, BioLegend); 1:100 dilution<br>*CD3 APC (cat#130-113-125, Miltenyi Biotec); 1:100 dilution<br>*CD95 PeCy7 (#cat 305622, BioLegend); 1:100 dilution<br>*CD62L PerCPCy5.5 (cat# 304824, BioLegend); 1:100 dilution<br>*CD45RA VioBlu (cat# 130-113-360, Miltenyi Biotec); 1:100 dilution

	*CD3 PcpCy5.5 (cat#300328, BioLegend); 1:100 dilution<br>*CD8 APC-H7 (cat#641400, BD); 1:100 dilution<br>*Annexin V APC (cat#640920, BioLegend); 1:100 dilution<br>*CD4 BV605 (cat#562658, BD); 1:100 dilution<br>*CD45RA PB (cat#304123, BioLegend); 1:100 dilution<br>*CD45 BUV395 (cat#563792, BD); 1:100 dilution<br>*CD62L BUV737 (cat#568329, BD); 1:100 dilution<br>*Fixable Viability Dye Viakrome 808 (cat#C36628, Beckman Coulter)<br>*C12FDG (cat#D2893, ThermoFisher); 1:1000 dilution<br>*SPiDER-βGal (cat#SG02-10, Dojindo); 1:1000 dilution<br>*mCD45 (cat#103108, BioLegend) 1:100 dilution<br>*hCD45 (cat#304016, BioLegend) 1:100 dilution<br>*antihuman FcR Blocking (cat#130-059-901, Miltenyi Biotec) 1:100 dilution<br>*antimouse FcR Blocking (cat#553142, BD) 1:200 dilution
Validation	All commercially sourced primary antibodies were validated by the respective manufacturers for the indicated species and applications, including flow cytometry and immunoblotting, as applicable. Manufacturer validation data, including specificity, tested applications and supporting experimental information, are available in the corresponding product datasheets using the catalogue numbers provided in the Methods and Reporting Summary. Antibodies were used according to the manufacturers' recommendations, and validation information is available in the corresponding product datasheets using the catalogue numbers provided in the Methods and Reporting Summary.

Eukaryotic cell lines

Policy information about cell lines and Sex and Gender in Research

Cell line source(s)	THP-1 cells were obtained from ATCC (cat. no. TIB-202), and BJ hTERT human fibroblasts were obtained from ATCC (cat. no. CRL-3627). The 5637 cell line was provided by collaborators and was used solely to generate conditioned medium for primary AML cultures; information on the original source and catalogue number was not available.
Authentication	THP-1 cell linTHP-1 and BJ hTERT cells were obtained from ATCC. The cell lines were not independently authenticated in our laboratory; their identities were based on the corresponding supplier documentation.
Mycoplasma contamination	All cell lines were routinely tested and resulted negative for the presence of mycoplasma contamination.
Commonly misidentified lines<br>(See ICLAC register)	THP-1 and BJ hTERT cells were checked against the ICLAC Register of Misidentified Cell Lines (version 14, released 15 February 2026); neither cell line was listed as misidentified.

Animals and other research organisms

Policy information about studies involving animals; ARRIVE guidelines recommended for reporting animal research, and Sex and Gender in Research

Laboratory animals	Female NSG mice (Mus musculus; NOD.Cg-Prkdc^scid^ Il2rg^tm1Wjl^/SzJ; NOD genetic background), aged 8–10 weeks, were obtained from Charles River Laboratories (Calco, Italy) and maintained under SPF conditions with a 12-h light/12-h dark cycle, at 22 ± 2 °C and 55 ± 5% relative humidity.
Wild animals	This study did not involve wild animals.
Reporting on sex	All in vivo experiments were performed using female mice. Sex was therefore not included as an experimental variable, and sex-stratified analyses were not performed because the study was not designed to evaluate sex-specific effects.
Field-collected samples	This study did not involve field-collected samples.
Ethics oversight	All animal procedures were approved by the Animal Care and Use Committee of IRCCS Ospedale San Raffaele and authorized by the Italian Ministry of Health in accordance with Italian legislation (IACUC protocol no. 1386).

Note that full information on the approval of the study protocol must also be provided in the manuscript.

Plants

Seed stocks

n.a.

Novel plant genotypes

n.a.

Authentication

n.a.

Flow Cytometry

Plots

Confirm that:

☒ The axis labels state the marker and fluorochrome used (e.g. CD4-FITC).

☒ The axis scales are clearly visible. Include numbers along axes only for bottom left plot of group (a 'group' is an analysis of identical markers).

☒ All plots are contour plots with outliers or pseudocolor plots.

☒ A numerical value for number of cells or percentage (with statistics) is provided.

Methodology

Sample preparation

The sample preparation and biological source of the cells were described in the Methods section of the manuscript.

Instrument

Immunophenotypic analyses were performed on FACS Canto II (BD Pharmingen), FACSymphony A5 SORP (BD Biosciences) or ImageStreamX instrument (Cytek®). Cell sorting was performed on a BD FACSAria Fusion (BD Biosciences).

Software

Flow Cytometry data were collected using the BD FACSDiva Software and analyzed with FlowJo (v10).

Cell population abundance

Leukemic blasts were isolated by FACS sorting based on the expression of CD34 and/or CD33 surface markers. The proportion of $CD34^+$ and/or $CD33^+$ cells within the diagnostic bone-marrow samples varied among patients, reflecting inter-patient heterogeneity in leukemic cell composition.
Cell population purity after sorting was checked by repeting FACS analysis.

Gating strategy

The gating strategies were described in the manuscript (methods section).

☒ Tick this box to confirm that a figure exemplifying the gating strategy is provided in the Supplementary Information.
