# Supplementary material for: Therapy induced senescence promotes immunogenicity in acute myeloid Leukemia through reduced EZH2 activity
Source: Nat Commun. 2026 Sep 4;17:9151. doi: 10.1038/s41467-026-76853-1 (PMC13545235; doi:10.1038/s41467-026-76853-1)
Supplement: Supplementary file 4 [file 41467_2026_76853_MOESM9_ESM.pdf]

nature portfolio

Peer Review File

Therapy Induced Senescence Promotes Immunogenicity in Acute Myeloid Leukemia through Reduced EZH2 Activity.

Corresponding Author: Professor Raffaella Di Micco

This file contains all reviewer reports in order by version, followed by all author rebuttals in order by version.

Version 0:

Reviewer comments:

Reviewer #1

(Remarks to the Author)
Therapy-induced senescence (TIS) has gained attention as a mechanism of therapy resistance in solid tumours and lymphomas. This study take a new angle and investigates TIS in acute myeloid leukemia. Using matched clinical samples collected before and after chemotherapy, the authors could stratify patients as well as previously published data sets into senescence and apoptotic responsive groups. They show that a pre-existing epigenetic signature predisposes certain samples to senescence, which is associated with higher expression of HLA class I and II molecules and broader presentation of disease-associated peptides capable of eliciting T cell responses. Finally, they propose that the clinically approved EZH2 inhibitor tazemetostat may sensitize apoptosis-prone AML cases to undergo senescence and promote immune-mediated clearance.
The manuscript is well written and the study is well performed with little experimental gaps. Addressing the following questions might help to further straighten the study.
Comments:
1. Fig. 5 H, I, J: EZH2 inhibition showed increases of HLA class II expression and T cell activation in Sen Low samples. How does this effect compare to Sen High samples? Also, was HLA class I expression assessed in this context, as it was earlier?
2. EZH2 inhibition is clinically actionable, which strengthens the translational relevance of the study. However, do the authors observe a sustained therapeutic benefit in Sen Low patient-derived samples? Additionally, how does the combinatorial treatment perform in the humanized mouse model when comparing Sen Low versus Sen High tumors?
3. Was tumor recurrence monitored in the humanized mouse model? If not, assessing this could strengthen the therapeutic impact of the findings - particularly to determine whether combinatorial therapy prevents relapse or whether Sen High tumors are inherently less prone to recurrence.
On the more technical note:
1. Consider overlaying the ridge plots for treatments to more clearly show distributional shifts beyond just peak or mean fluorescence.
2. To better highlight intra-sample trends in antigen processing and presentation, consider standardizing the sample order across both panels in Fig. 2A and B.

Reviewer #2

(Remarks to the Author)
Gilioli et al. report that chemotherapy induces therapy-induced senescence (TIS) in a subset of AML samples, leading to enhanced interferon signaling, HLA upregulation, and presentation of leukemia- and senescence-associated peptides. These changes confer antigen-presenting cell-like properties to AML blasts and promote autologous T cell activation both ex vivo and in PDX models. Importantly, TIS restores sensitivity to immune checkpoint blockade and is mechanistically linked to reduced PRC2 activity. Pharmacological inhibition of PRC2 reactivates senescence-related genes and boosts HLA expression in non-senescent AML blasts, enabling T cell responses. The manuscript is well written and structured, but modifications and clarifications are necessary as described below.

1. The classification of patients into "Sen High" and "Sen Low" needs to be more objective. At present, the criterion seems somewhat arbitrary; I recommend defining a quantitative cutoff, such as a specific fold-change of SA-β-Gal activity after AraC, to improve the robustness and reproducibility of the analysis (Fig. 1B).

The conclusion that chemotherapy induces senescence would be strengthened by additional validation using canonical senescence markers, such as p21^CIP1 (via western blot or flow cytometry) and DNA damage foci (γH2AX).
2. Given that scRNA-seq data from public cohorts are already mentioned, I recommend performing a dedicated analysis in ex vivo treated samples to determine whether HLA upregulation and senescence gene expression occur in the same cells or in distinct subpopulations, thereby revealing potential heterogeneity of TIS at the single-cell level.
3. The proposed mechanistic model could be explored more convincingly. It would be important to test whether PRC2 loss is sufficient to activate IFN signaling. Knockdown of EZH2 in "Sen Low" cell lines, followed by assessment of p53 phosphorylation and IFN production, could help establish a causal hierarchy among these events.
4. The immunopeptidomics data are promising but remain somewhat superficial. I suggest investigating whether the peptides uniquely found in treated "Sen High" samples derive from TAAs, senescence-associated antigens, or neoantigens. Functional validation of one or two candidate peptides in T cell activation assays would be a valuable addition.
5. Further exploration of the mechanism underlying PRC2 downregulation in "Sen Low" is warranted. Analysis of EZH2, SUZ12, and EED expression, as well as regulators such as ASXL1, could provide insights into the epigenetic profile observed (Fig. 5).
6. The Tazemetostat experiment (Fig. 5F-J) is convincing but lacks an essential control: treatment of "Sen High" samples. This would clarify whether EZH2 inhibition specifically reverts the "Sen Low" phenotype or broadly enhances immunogenicity across all AML samples.
7. It would be useful to expand the discussion of TIS temporal dynamics, as the study focuses on a 5-day window. Public datasets suggest that senescence may be transient; therefore, discussing how this temporal aspect impacts the proposed therapeutic strategy would be valuable.
8. To reinforce the claim that T cell activation is specific to leukemia/senescence antigens, I recommend including additional assays such as IFN-γ ELISpot or cytotoxicity assays using expanded T cells against autologous senescent versus non-senescent blasts.
9. In the PDX model (Fig. 4F-K), the analysis centers on human blasts. It would be informative to also assess T cell infiltration and activation status in the bone marrow microenvironment, using immunohistochemistry or flow cytometry for memory and exhaustion markers.
10. The discussion should also acknowledge that EZH2 inhibition may have pleiotropic effects. The improved immunogenicity observed in "Sen Low" samples treated with Tazemetostat may not be exclusively mediated by senescence induction but also by other PRC2-regulated pathways.
11. The correlation between "Sen High/Sen Low" status and clinical outcomes is particularly relevant. If feasible, inclusion of Kaplan-Meier analyses or complete remission response data, even in a small cohort, would substantially enhance the translational impact.
12. Please also justify the choice of the 400 nM AraC dose used ex vivo (Fig. 1A) relative to plasma concentrations achieved in patients, and discuss the relevance of the 60 mg/kg dose in the PDX model compared with the standard clinical "7+3" regimen.
13. I recommend including a more comprehensive clinical data table in the main manuscript, covering cytogenetic/molecular risk (ELN), blast counts, and post-induction outcomes, to allow readers a better understanding of the study cohort.
14. Finally, some figure legends and descriptions require clarification. In Fig. 1C, the exact meaning of the "Interaction-Log2FC" axis should be explained; in Fig. 2J, the enrichment analysis of peptides should be described more clearly in the main text; and, in general, the statistical tests used in the LME models should be indicated more explicitly either in the figure legends or results section.

Reviewer #3

(Remarks to the Author)
The manuscript "Therapy-Induced Senescence Promotes Immunogenicity in Acute Myeloid Leukemia Blasts through loss of PRC2-Mediated Gene Regulation" from Gilioli, Fusco and colleagues show that upon treatment a proportion of the AML patients respond with therapy-induced cellular senescence (TIS) that is characterised by senescence-associated secretory phenotype (SASP). Furthermore, they show evidence that this phenotype provides with beneficial for the cancer clearance immunogenicity by HLA-I and HLA-II upregulation and subsequent CD8+ and CD4+ cell response. Finally, in the proportion of patients where TIS is not induced the authors suggest the existence of an underlying epigenetic vulnerability by the accumulation of negative chromatin marks such as H3K27me3 that may further be exploited in novel treatment approaches against AML. The study is thorough and well-build, and the manuscript is well-written with very clear graphical presentation.

I find the work of Gilioli, Fusco and colleagues to be of significance to the AML field where to the best of my understanding no previous reports have been published about the utility of TIS in particularly AML. I find the work also of interest to the wider cancer haematology and related fields.

Previous authors have defined the therapy-inducible SA-β-gal-based senescence responsiveness as either senescence-competent or -incompetent for what Gilioli, Fusco and colleagues define as Sen High and Sen Low patients/response, respectively (Belenki D, et al. Nat Commun. 2025 Mar 31;16(1):3079. doi: 10.1038/s41467-025-57429-x. PMID: 40159497; PMCID: PMC11955568.). As both groups ultimately strive to establish the importance of the same phenomenon, I would suggest it is beneficial to adhere to the same terminology especially since Gilioli, Fusco and colleagues do use the term "senescence competence" when they discuss their data.

The work is well-executed and presents well-substantiated conclusions and claims. My main concerns with the study remain the balance of the double-sided nature of TIS and SASP in cancer and whether the phenomena may also negatively affect the long-term clearance of AML cells. Would it be possible for the authors to deepen their analysis and discussion on the matter?

Furthermore, the authors state that “neither the mutational profile nor the karyotypic status explained the senescence-based stratification of our cohort” but this at present is not entirely convincing because of the small size of the cohort. If possible, the authors should increase their cohort and provide cytogenetics for those patients who are currently lacking.

Is it possible that younger (female?) patients are more likely to have senescence-competent AML? If the authors increase their patent pool, they could include appropriate statistics on the effect of age and gender distribution? The authors should at least discuss the possible implications.

The authors state “Sen Low patients present with inferior long-term outcome to treatment (R. Di Micco and C. Schmitt, personal communications)”. This is potentially very interesting and thus should not be provided without the support of the actual data that can be evaluated. The authors must provide the data or remove any personal communication statements.

It would be of great interest if the authors provide in vivo validation of tumour clearance after co-culture of TAZ-treated AML blasts from Sen Low samples in the humanized NSG mouse model.

In the current experiment with Sen High blasts in the humanized NSG mice (experiment presented in figures 4F - K) it seems that in some groups about 50% of the mice were excluded from the analysis whereas I could not find an explanation to why and as such this undermines the conclusions. Better explanation and evaluation of the possible impact is warranted.

Could the authors provide any evaluation on how persistent are TIS/SASP in patients or in vitro from the data available and discuss any possible negative implications?

The authors should provide proof that the H3K27me3 enrichment over the affected loci presented in figure 5G was depleted after TAZ treatment or at least global depletion of the mark.

Minor comments:
a. SAHF is senescence-associated heterochromatin foci, on line 165 “foci” is missing.
b. On line 286, tazemetostat whereas on line 989 is Tazemetostat, please adhere to one and see reference article.
c. Spell out NES as Normalized Enrichment Score the first time it occurs.

Version 1:

Reviewer comments:

Reviewer #1

(Remarks to the Author)
The authors addressed the comments nicely and successfully.

Reviewer #2

(Remarks to the Author)
The authors have responded comprehensively and constructively to the reviewers' comments, providing substantial new experimental data, additional in vivo validation, expanded mechanistic analyses, and important clarifications throughout the manuscript.

Reviewer #3

(Remarks to the Author)
The authors have carefully addressed all the concerns raised during the review process, thoroughly revised the manuscript, and significantly improved the clarity and quality of their work. I appreciate their detailed responses and all revisions made throughout the manuscript.

I have no further major concerns and believe the manuscript is now suitable for publication.

Open Access This Peer Review File is licensed under a Creative Commons Attribution 4.0 International License, which permits use, sharing, adaptation, distribution and reproduction in any medium or format, as long as you give appropriate credit to the original author(s) and the source, provide a link to the Creative Commons license, and indicate if changes were made.
In cases where reviewers are anonymous, credit should be given to 'Anonymous Referee' and the source.
The images or other third party material in this Peer Review File are included in the article's Creative Commons license, unless indicated otherwise in a credit line to the material. If material is not included in the article's Creative Commons license and your intended use is not permitted by statutory regulation or exceeds the permitted use, you will need to obtain permission directly from the copyright holder.
To view a copy of this license, visit https://creativecommons.org/licenses/by/4.0/

REVIEWER COMMENTS

Reviewer#1

Therapy-induced senescence (TIS) has gained attention as a mechanism of therapy resistance in solid tumours and lymphomas. This study takes a new angle and investigates TIS in acute myeloid leukemia. Using matched clinical samples collected before and after chemotherapy, the authors could stratify patients as well as previously published data sets into senescence and apoptotic responsive groups. They show that a pre-existing epigenetic signature predisposes certain samples to senescence, which is associated with higher expression of HLA class I and II molecules and broader presentation of disease-associated peptides capable of eliciting T cell responses. Finally, they propose that the clinically approved EZH2 inhibitor tazemetostat may sensitize apoptosis-prone AML cases to undergo senescence and promote immune-mediated clearance. The manuscript is well written and the study is well performed with little experimental gaps. Addressing the following questions might help to further straighten the study.

We thank the Reviewer for the careful evaluation of the study and for acknowledging that our work is novel, important, well-written and with little experimental gaps. We hope that they will be satisfied with the revised version of the manuscript that addresses the remaining concerns.

Comments:

1. Fig. 5 H, I, J: EZH2 inhibition showed increases of HLA class II expression and T cell activation in Sen Low samples. How does this effect compare to Sen High samples? Also, was HLA class I expression assessed in this context, as it was earlier?

To address the point raised, we now provide a side-by-side comparison of HLA levels upon EZH2 inhibition via Tazemetostat (TAZ) treatment in additional Sen Low samples (now up to n = 7 paired samples, UT *vs* TAZ) and in Sen High samples (n = 5 paired samples, UT *vs* TAZ). In Sen Low, the inclusion of additional data confirmed a significant upregulation of HLA class II (HLA-DR^{+} cells) and HLA class I (HLA-ABC MFI) upon TAZ (paired Wilcoxon test; **Panel A**, below). In contrast, AraC in Sen Low samples did not induce HLA class I and II upregulation (**Fig. 2C, D** of the main manuscript). In Sen High samples TAZ treatment did not increase HLA class II levels, whereas it induced a significant increase in HLA class I (**Panel B**, below). Of note, AraC treatment in Sen High samples had stronger effects on HLA class I and HLA class II levels compared to TAZ treatment (**Fig. 2C, D** of the main manuscript).

The strongest effects of TAZ treatment in HLA I and HLA II upregulation in Sen Low samples was in line with their higher baseline PRC2/EZH2 activity. Indeed, nuclear proteomics showed increased H3K27me3 expression in Sen Low blasts (**Fig. 5C** of the main manuscript), which was further supported by higher levels of the repressive histone mark by western blot analysis (**Fig. 5F**), together with increased EZH2 expression at both transcript (**Supplementary Fig. 5D**) and protein levels (**Fig. 5F**). This provides a mechanistic rationale for the greater sensitivity of Sen Low samples to EZH2 inhibition. See also our response to Reviewer #2, comment 5.

We have updated the graphs of Sen Low patients in **Fig. 6F**, including the new data on Sen High samples in **Supplementary Fig. 6C** of the manuscript and revised the text accordingly.

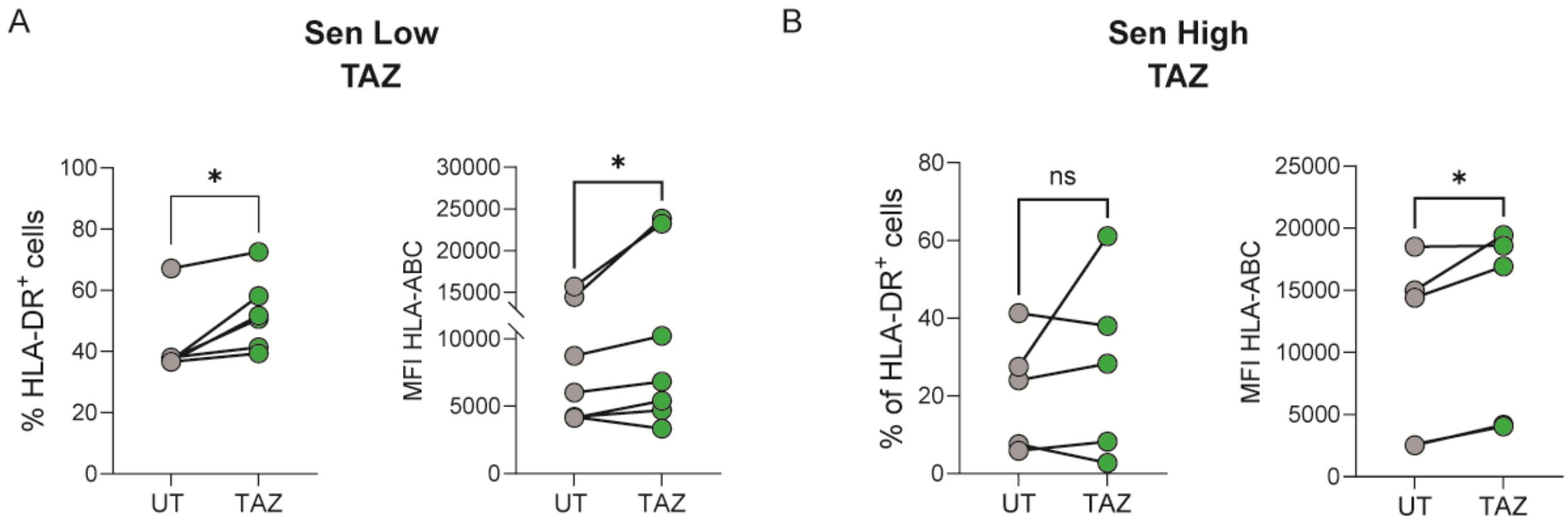

2. EZH2 inhibition is clinically actionable, which strengthens the translational relevance of the study. However, do the authors observe a sustained therapeutic benefit in Sen Low patient-derived samples? Additionally, how does the combinatorial treatment perform in the humanized mouse model when comparing Sen Low versus Sen High tumors?

First, to address the requested Sen Low versus Sen High comparison in the AraC-T cells combinatorial setting, we performed an additional *in vivo* experiment in Sen Low PDX using the same AraC-based regimen and autologous T-cell adoptive transfer strategy applied in the Sen High model (see **Fig. 4F** of the main manuscript; daily administration of AraC for 5 consecutive days followed by a short drug holiday; two i.v. injections of previously *in vitro* expanded autologous T cells).
AraC induced a marked reduction in leukemia burden in both bone marrow and spleen, consistent with its debulking effect (**Panel A**, below). In line with *in vitro* data (**Fig. 1B, Fig. 2C** of the main manuscript), we did not observe increased levels of HLA class I (**Panel B**, below) or accumulation of senescence by SA-β-Gal activity (**Panel C**, below) on residual leukemic cells, suggesting no establishment of a TIS-linked immunogenic state despite effective cytoreduction. Consistently, subsequent administration of *in vitro* expanded autologous T cells (see characterization in new **Supplementary Fig. S4M** of the main manuscript) did not confer an additional measurable reduction in leukemia burden at endpoint when comparing AraC versus AraC + T cell groups (**Panel D**, below) as opposed to the Sen High model, where AraC was associated with stronger senescence/immunogenic features and a more evident functional impact of autologous T-cell administration (**Fig. 4J, K** of the main manuscript). These data support the notion that Sen Low and Sen High AML do not differ in their susceptibility to chemotherapy-mediated debulking, but rather in their capacity to mount an early TIS-linked immunogenic response that can be leveraged by autologous T cells administration.

We have included the data obtained in bone marrow in the revised manuscript (new **Figure 4L-O**).

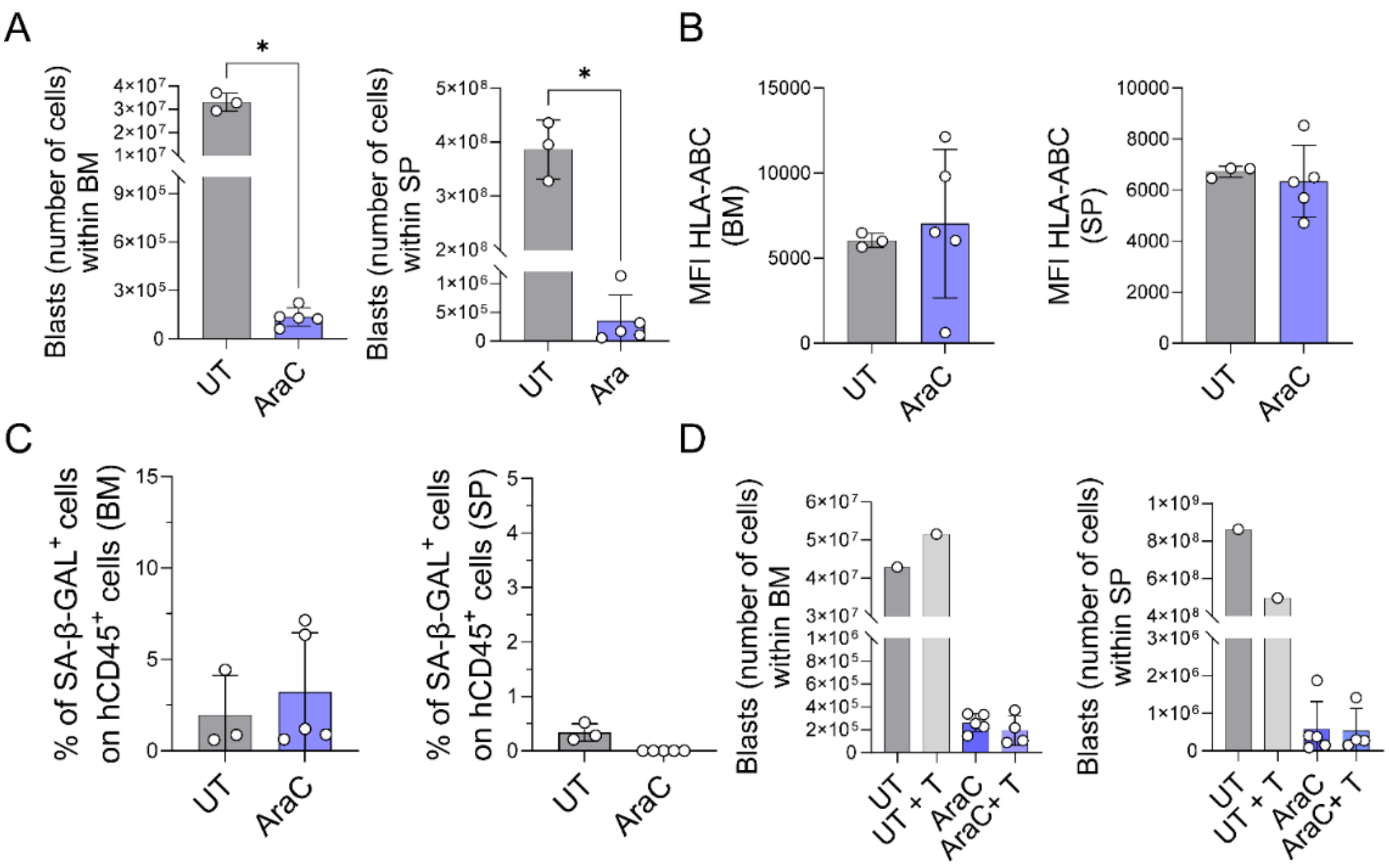

Next, to evaluate the impact of EZH2 inhibition *in vivo*, we transplanted NSG mice with patient-derived AML blasts from a Sen Low sample and, after confirming leukemia engraftment by bone marrow aspirates, we administered the EZH2 inhibitor TAZ (300 mg/kg, oral gavage, daily for 7 days, see **Panel A** below). At day 8 post-treatment, we observed a clear reduction of global H3K27me3 levels in leukemic cells from TAZ-treated animals compared to controls (**Panels B, C**, below). TAZ treatment did not reduce leukemia burden (**Panel D**, below), but it induced a significant increase in HLA class I expression (**Panel E**, below), consistent with its immune-priming effects.

In a group of TAZ-treated animals we subsequently administered patient-matched autologous T cells, previously expanded *in vitro*. However, in this experiment, due to limited availability of the autologous T-cell product, we performed only one i.v. infusion rather than two as in previously described in vivo experiments. At endpoint, we observed a trend toward reduced leukemia burden in the TAZ + autologous T-cell condition compared with TAZ alone (**Panel F**, below). Importantly, this trend emerges despite a reduced T-cell dosing intensity, suggesting that EZH2 inhibition can sensitize Sen Low AML to immune-mediated control; a full two-T cell infusion schedule would be expected to further strengthen this effect. We have now included these data in **Fig. 6H-L** of the main manuscript and added a brief section in the Discussion explicitly acknowledging the limitations of this in vivo setting and outlining it as a key consideration for future longitudinal studies.

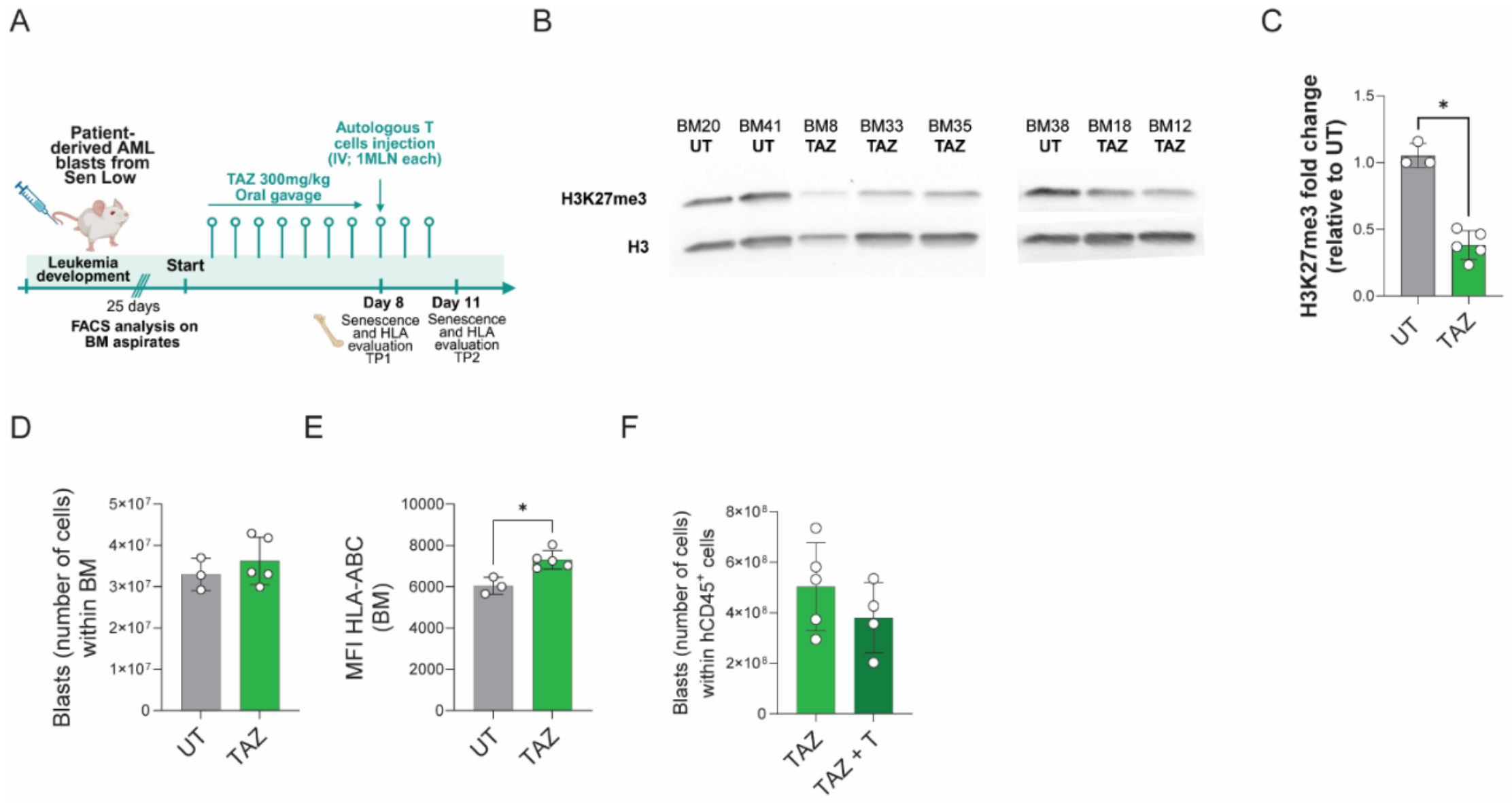

With respect to the Reviewer’s question on a “sustained” therapeutic benefit, we would like to clarify that the present study is focused on early treatment response and immediate immunogenic/immune-priming changes following therapy. Accordingly, our *in vivo* experiments were intentionally endpoint-based and were not designed to evaluate long-term disease control, recurrence, or survival. Nonetheless, in the revised Discussion we outline long-term consequences of TIS establishment as an important direction for future work.

3. Was tumor recurrence monitored in the humanized mouse model? If not, assessing this could strengthen the therapeutic impact of the findings - particularly to determine whether combinatorial therapy prevents relapse or whether Sen High tumors are inherently less prone to recurrence.

We thank the Reviewer for this suggestion. The study was designed to investigate early treatment responses and failure, in particular the role of TIS establishment and the associated immunogenic/antigen-presentation rewiring, using predefined endpoint-based readouts rather than long-term longitudinal follow-up. Thus, we did not monitor tumor recurrence/relapse in the PDX model. However, to address the translational relevance of our findings, we would like to provide complementary clinical evidence supporting the significance of TIS competence in clinical settings. In our patient cohort (Milan Cohort), Sen High cases, defined by an AraC-induced increase in SA-β-gal positivity measured *ex vivo*, correspond to patients clinically defined as “responders” (residual AML less than 5% at day 14 or 30 post in vivo chemotherapy), whereas Sen Low cases, defined by the absence of such *ex vivo* SA-β-gal induction, correspond to clinically-defined “non-responders” (residual AML more than 5% at day 14 or 30 post in vivo chemotherapy) (**Panel A**, below). These data are presented in a companion manuscript, that we co-authored, by C. Schmitt’s team currently under invited revision at *Nature Communications* with potential for back-to-back publication.

In the same clinical work, stratification based on an *ex vivo* SA-β-Gal readout (TIS-High vs TIS-Low) in two independent cohort of patient samples (Berlin cohort, n=39, Munich cohort, n=37) also correlated with improved outcomes including disease-free survival and overall survival in TIS-high *vs* TIS-Low patients (**Panel B,C,** below).

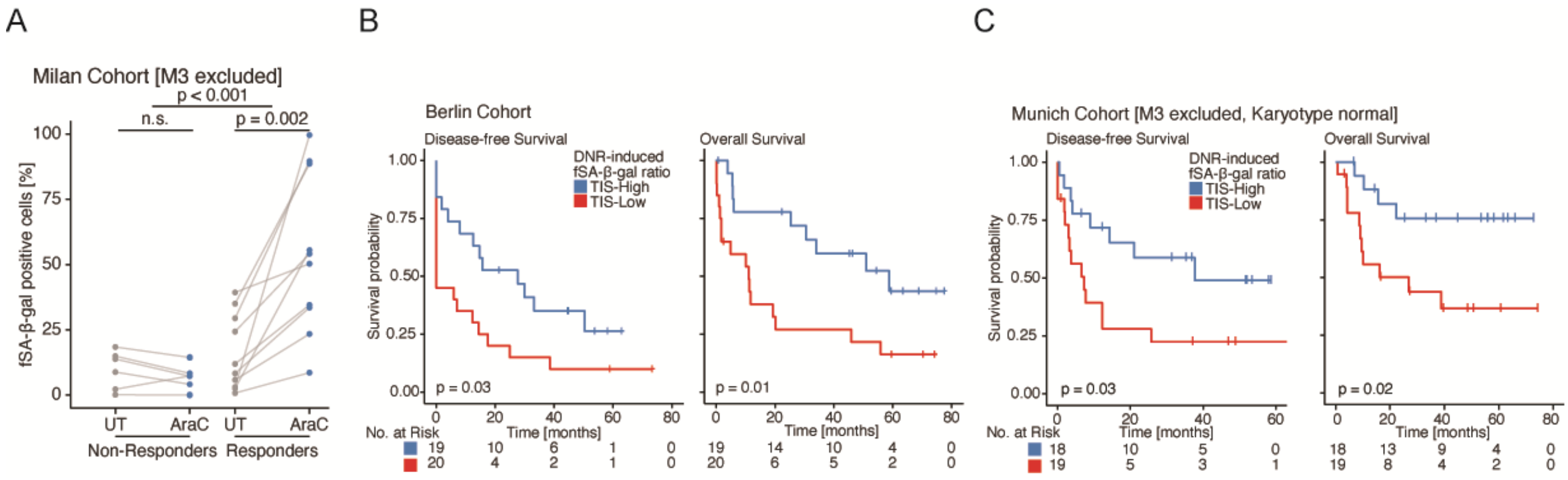

Finally, given the strong immunogenic component emerging from our mechanistic study, we also performed a novel analysis to investigate whether TIS competence associates with better outcome after allogeneic-hematopoietic stem cell transplantation (allo-HSCT), which in AML represents the most established form of immune-mediated disease control.

We combined clinical data from patients across three cohorts (i.e. Milan, Berlin, and Munich) who underwent allo-HSCT. Patients who relapsed before transplant were excluded. To ensure consistency across cohorts, the analysis was restricted to cases with normal karyotype, as one contributing cohort (Munich) included only patients with normal karyotype. The final study population comprised 25 patients.

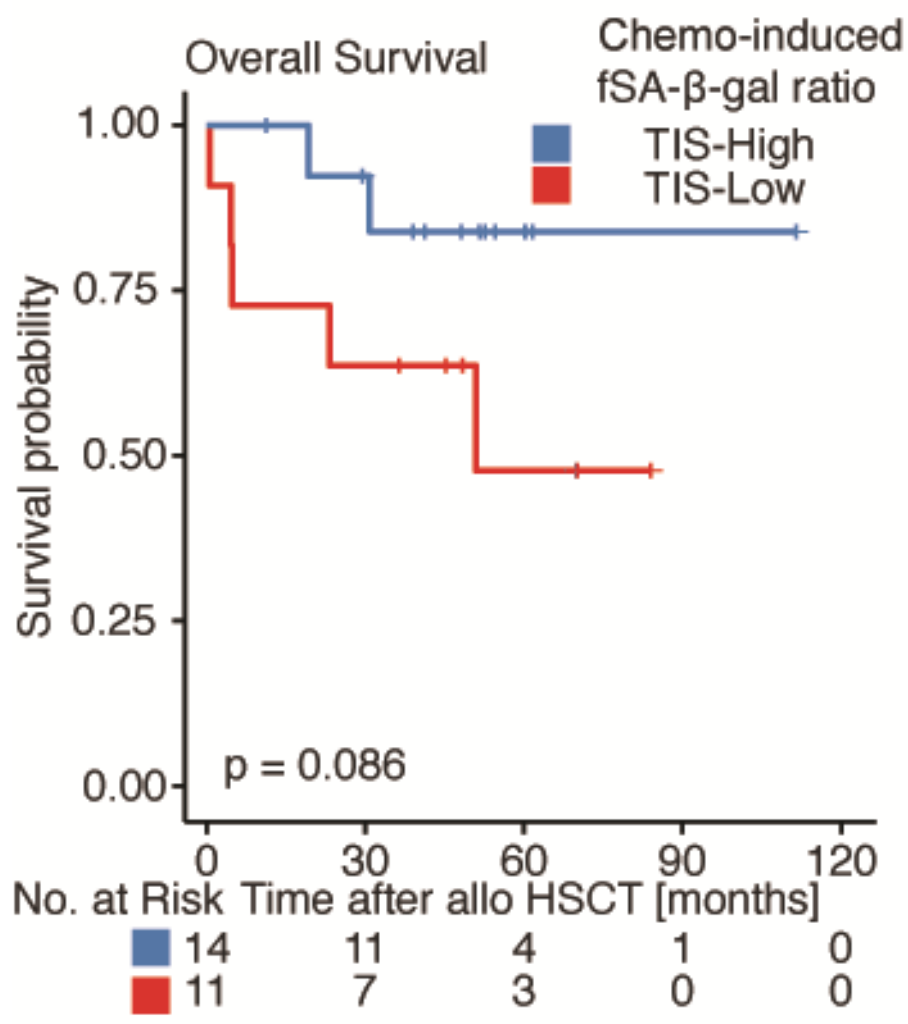

As shown in the figure on the right, TIS-high patients had improved overall survival after allo-HSCT compared with TIS-low patients, with a clear separation of the Kaplan–Meier curves over time and a p value approaching statistical significance (log-rank p = 0.086). We provide these data for Reviewer and Editor only, given the limited sample size after clinical filtering. However, this trend is consistent with the concept that TIS competence associates with a more immunologically controllable disease state and may positively influence outcome in the context of immune-mediated therapies such as allo-HSCT.

On the more technical note:

1. Consider overlaying the ridge plots for treatments to more clearly show distributional shifts beyond just peak or mean fluorescence.

We have revised **Supplementary Fig. 1G** by overlaying the ridge plots for the two treatment conditions (UT *vs* AraC) in both Sen Low and Sen High samples (see below). This representation more clearly highlights the treatment-associated distributional shift of the senescence marker and we have updated the figure legend accordingly.

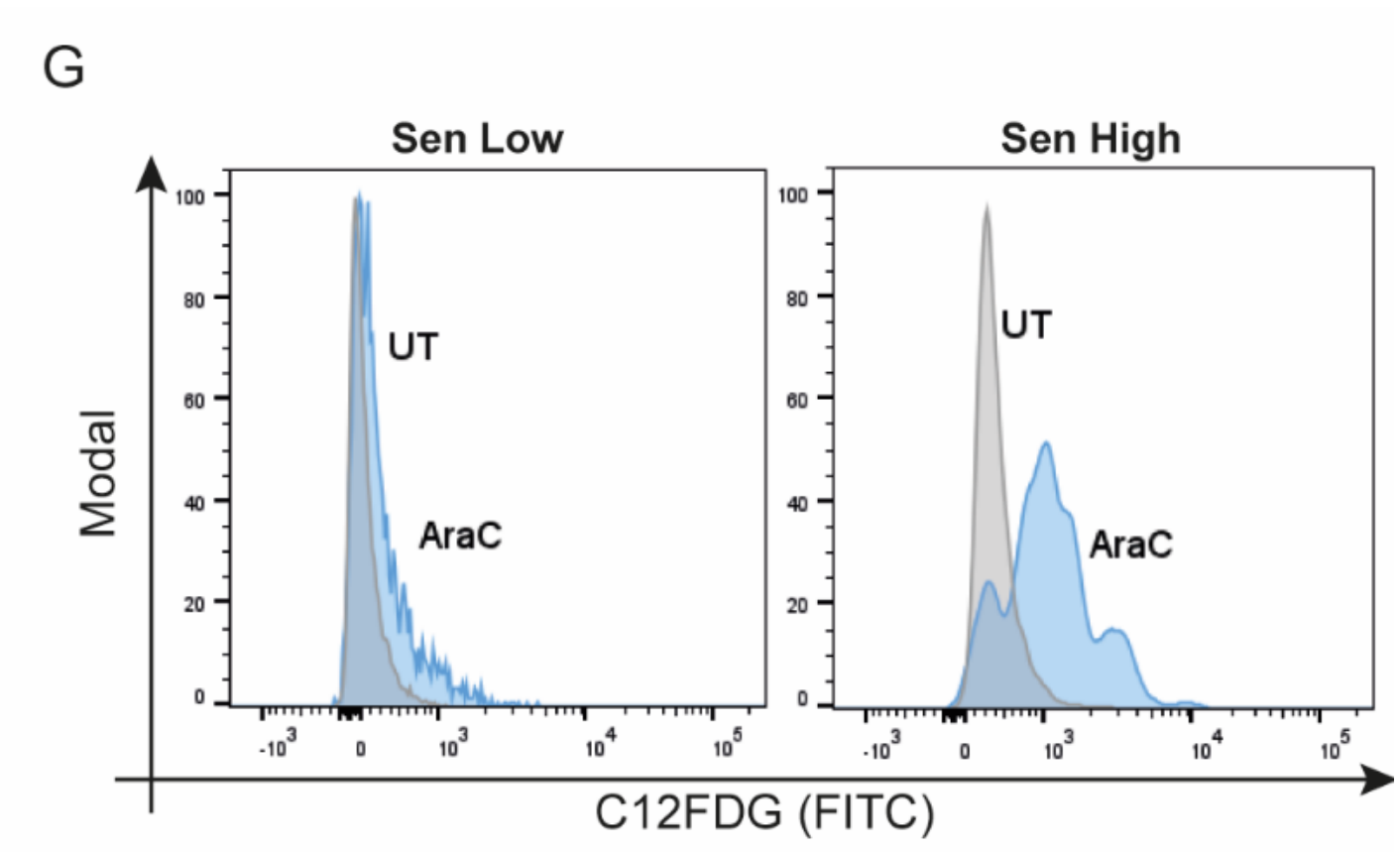

2. To better highlight intra-sample trends in antigen processing and presentation, consider standardizing the sample order across both panels in Fig. 2A and B.

In the original version, the sample order differed because each heatmap was displayed according to hierarchical clustering. We have now standardized the sample order across **Fig. 2A, B** (see **Panel A, B** below) to better highlight intra-sample trends and revised the figure and the figure legend accordingly.

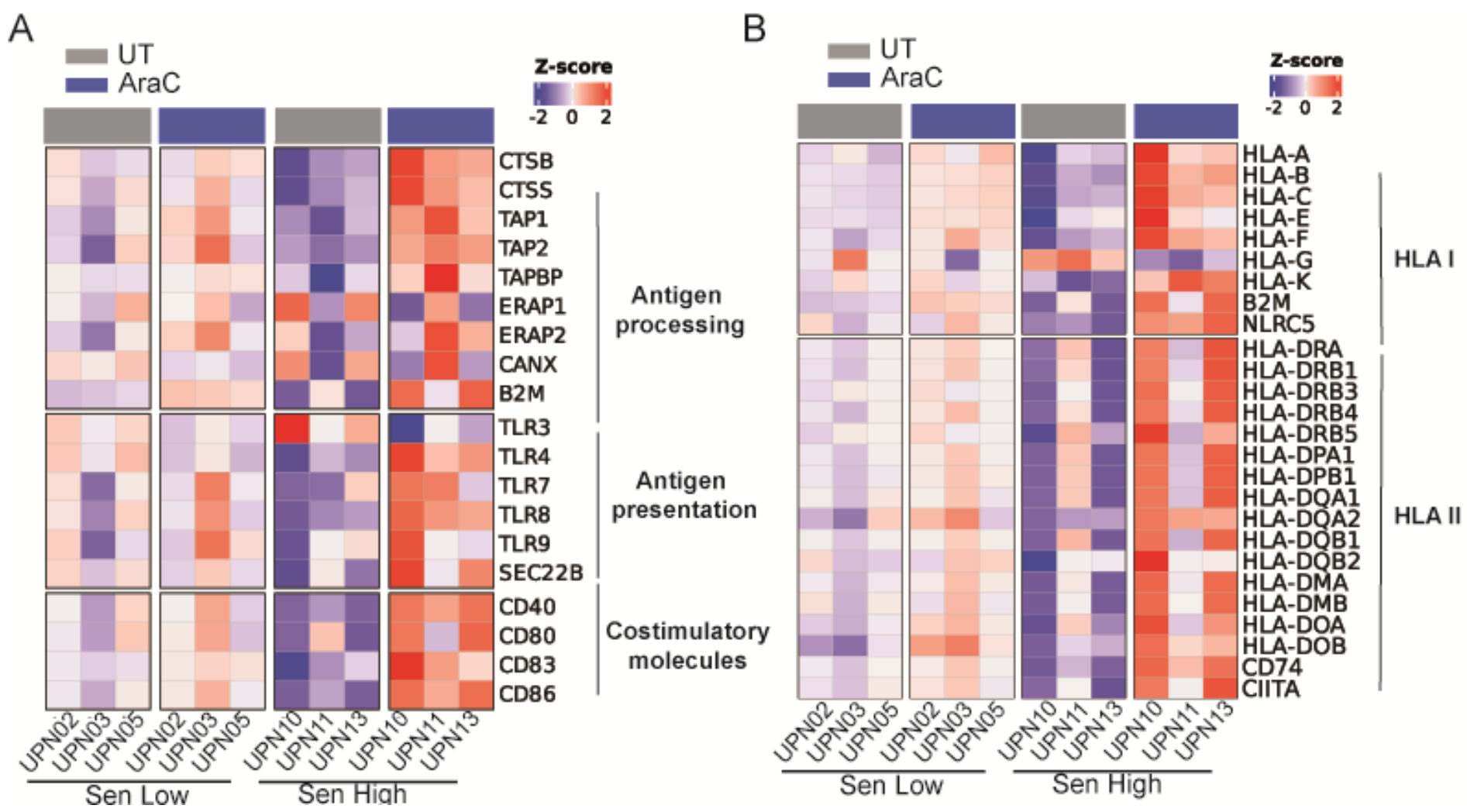

Reviewer #2 (Remarks to the Author):

Gilioli et al. report that chemotherapy induces therapy-induced senescence (TIS) in a subset of AML samples, leading to enhanced interferon signaling, HLA upregulation, and presentation of leukemia- and senescence-associated peptides. These changes confer antigen-presenting cell-like properties to AML blasts and promote autologous T cell activation both *ex vivo* and in PDX models. Importantly, TIS restores sensitivity to immune checkpoint blockade and is mechanistically linked to reduced PRC2 activity. Pharmacological inhibition of PRC2 reactivates senescence-related genes and boosts HLA expression in non-senescent AML blasts, enabling T cell responses. The manuscript is well written and structured, but modifications and clarifications are necessary as described below.

We thank the Reviewer for the careful evaluation and constructive feedback. We appreciate the positive assessment of the manuscript's clarity and structure. We have addressed the requested concerns both experimentally and with changes in the main text discussion.

1. The classification of patients into "Sen High" and "Sen Low" needs to be more objective. At present, the criterion seems somewhat arbitrary; I recommend defining a quantitative

cutoff, such as a specific fold-change of SA-β-Gal activity after AraC, to improve the robustness and reproducibility of the analysis (Fig. 1B).

We have now revised the Methods and Results section to state more clearly the classification rule used, based on fluorescent SA-β-Gal measurements before and after AraC treatment *ex vivo*. Specifically, in our study samples were defined as Senescence High (Sen High) when they met both criteria: (i) post-AraC SA-β-Gal positivity >10%, and (ii) a positive fold-change relative to the untreated condition. Samples that did not meet these criteria were defined as Senescence Low (Sen Low). This definition captures bona fide AraC-inducible senescence responses while excluding samples with low post-treatment signal or no senescence increase compared to baseline.

Importantly, this classification is further supported by independent molecular readouts. Sen High samples consistently display higher expression of senescence-associated genes, including GLB1 (encoding SA-β-Gal, see **Supplementary Fig. S1K**), CDKN1A/p21 and CDKN2A/p16 (**Fig. 1E**, **Supplementary Fig. S1L, M**), together with enrichment of senescence-related transcriptional programs (i.e. p53 pathway, inflammatory response, see **Fig. 1C**) by GSEA in our bulk RNA-seq analyses. Thus, the SA-β-Gal–based stratification is corroborated by orthogonal transcriptomic evidence and identifies a biologically coherent senescence-competent AML subgroup.

The conclusion that chemotherapy induces senescence would be strengthened by additional validation using canonical senescence markers, such as p21^CIP1 (via western blot or flow cytometry) and DNA damage foci (γH2AX).

We agree that the inclusion of additional senescence markers would strengthen our conclusions. While SA-β-Gal is used as our primary senescence readout, we leveraged our RNA-seq (**Fig. 1C-F, Supplementary Fig. 1K** of the main manuscript) as an additional validation layer of the senescence response. Specifically, Sen High samples showed enrichment of transcriptional programs associated with senescence, including p53 pathway, DDR-related signatures, SASP/inflammatory programs, and curated senescence-associated gene sets, supporting the robustness of the stratification beyond a single cellular SA-β-Gal assay. In addition, we now explicitly report the gene expression levels of *CDKN2A* (p16) and *CDKN1A* (p21), showing that were both upregulated across Sen High samples upon AraC treatment compared to Sen Low, in line with a senescence-associated cell-cycle arrest (**Panel A,** new **Fig. 1E** of the main manuscript).

We also assessed p21 protein levels by western blot in available samples from both Sen Low and Sen High groups and observed a statistically significant increase in p21 levels in Sen High samples following AraC treatment (**Panel B** below, n=5 for each group). We also evaluated the levels of γH2AX, a marker and signal amplifier of the DDR cascade, and found higher γH2AX protein levels in Sen High samples treated with AraC compared to Sen Low, consistent with a stronger DDR signaling (**Panel C** below, n=3 for each group), despite comparable induction of cell cycle arrest in vitro (**Supplementary Fig. S1D, E**) and tumor clearance *in vivo* (**Fig. 4L**) upon AraC treatment.

We have now incorporated these data into the revised manuscript (**Fig. 1E**, **Supplementary Fig. 1L, M** of the main manuscript) and revised the text accordingly.

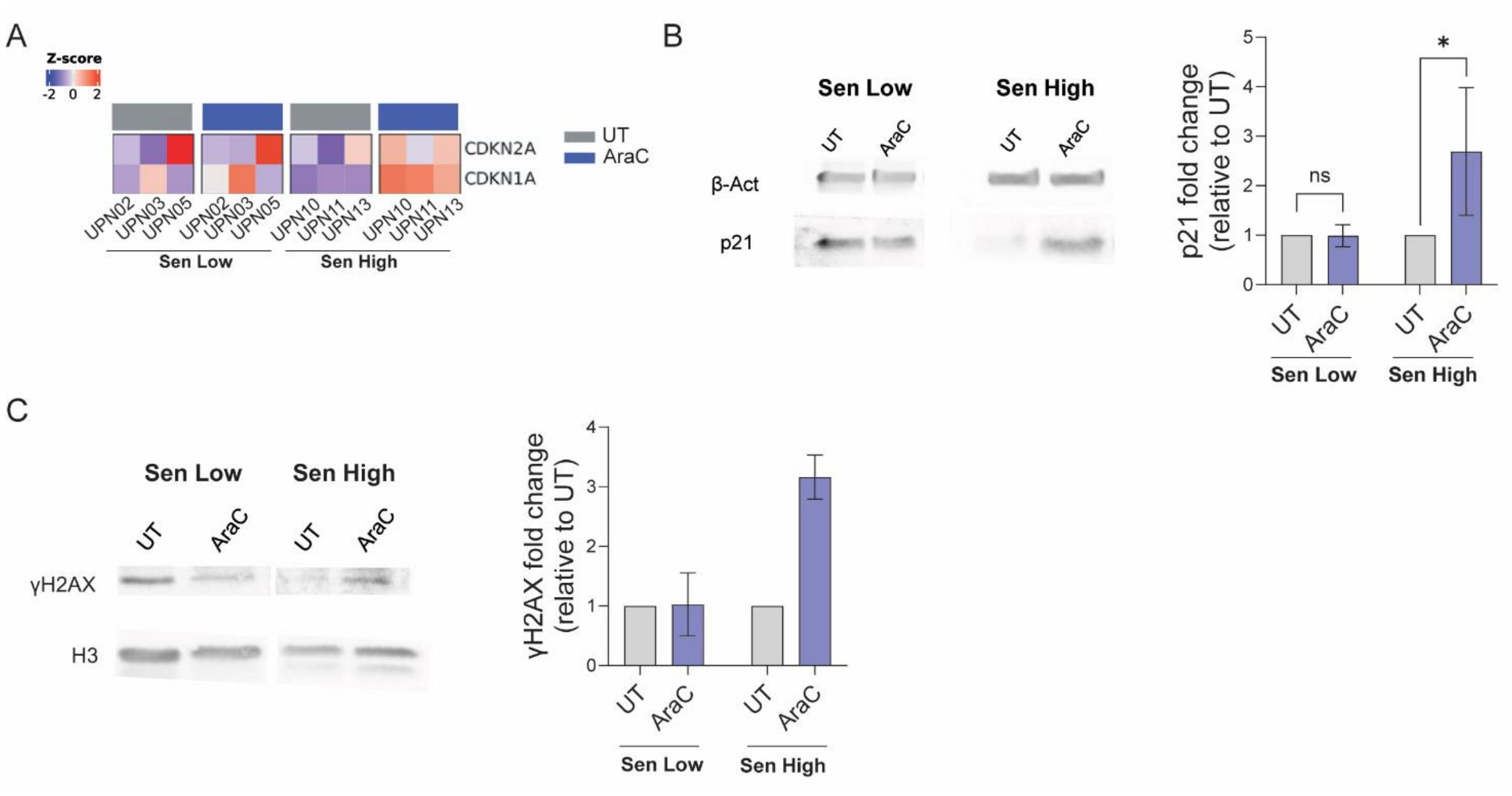

2. Given that scRNA-seq data from public cohorts are already mentioned, I recommend performing a dedicated analysis in *ex vivo* treated samples to determine whether HLA upregulation and senescence gene expression occur in the same cells or in distinct subpopulations, thereby revealing potential heterogeneity of TIS at the single-cell level.

We addressed this point by leveraging the publicly available *ex vivo* AraC-treated scRNA-seq dataset from Duy C. *et al.* (Cancer Discovery, 2021) which provides the experimental setting requested.

First, the question of co-occurrence versus segregation was supported by the analysis we already included in the manuscript: indeed, in the Duy *et al.* dataset, the clusters with the strongest enrichment for a senescence module score (0, 4 and 6) also show increased expression of HLA class I and class II, indicating that senescence-like programs and HLA upregulation are enriched within the same single-cell clusters rather than being uniformly distributed across all leukemic cells (see **Supplementary Fig. 2B** of the main manuscript).

To further strengthen the mechanistic interpretation and characterize the identity of these senescence/HLA-high AML subpopulations, we performed a dedicated reference-mapping analysis of the Duy *et al.* dataset using multiple independent references. Specifically, we mapped Duy *et al.* clusters (**Panel A**) onto (i) two well-annotated healthy bone marrow atlases and (ii) independent AML scRNA-seq references, including NPM1- and IDH-mutant AML cohorts. Across references, the senescence-enriched clusters consistently mapped to a myeloid/monocytic trajectory (**Panel B**). Importantly, prediction scores from the mapping were used to assess annotation confidence and to minimize bias from any single reference (**Panel C**).

Consistent with this, when we examined canonical monocytic markers across all Duy *et al*. clusters, clusters 0, 4 and 6, which showed the strongest enrichment of senescence programs and HLA, also displayed the highest expression of CD14, CD36, CD68, S100A8, S100A9 and MNDA markers (**Panel D**), supporting a monocyte-primed transcriptional state of chemotherapy-induced senescent AML cells.

Overall, these complementary analyses indicate that AraC-induced senescence states in AML preferentially arise within a CD14 monocyte-primed myeloid subset and are accompanied by HLA class I/II upregulation, reinforcing our model that early chemotherapy responses can couple senescence programs with increased immune visibility.

We have now included these data in the revised version of the manuscript (**Supplementary Fig. 2E, F**).

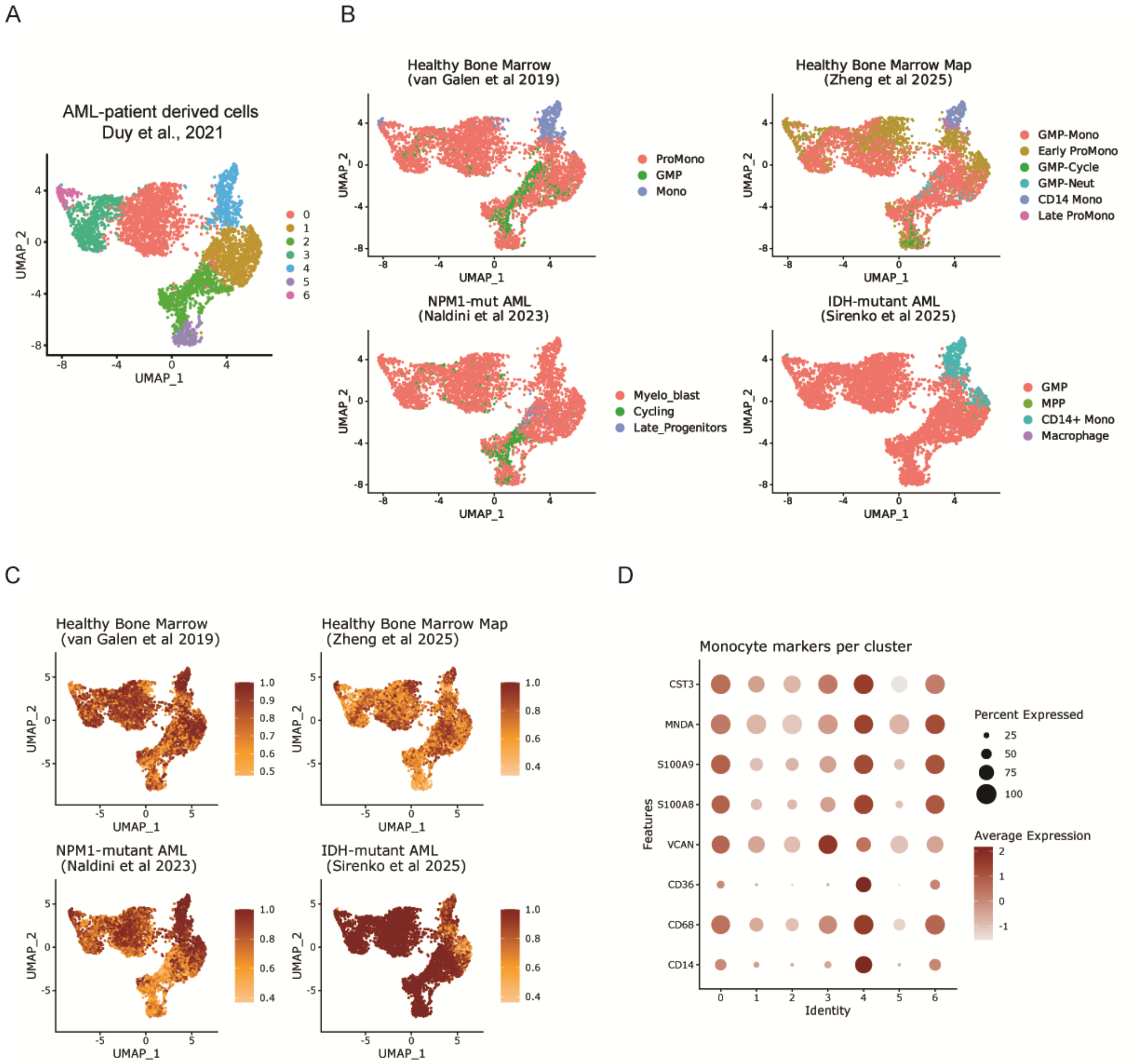

3. The proposed mechanistic model could be explored more convincingly. It would be important to test whether PRC2 loss is sufficient to activate IFN signaling. Knockdown of EZH2 in "Sen Low" cell lines, followed by assessment of p53 phosphorylation and IFN production, could help establish a causal hierarchy among these events.

We agree with the Reviewer that genetic perturbation experiments would further strengthen causal inference. However, we would like to highlight that we i) currently lack a bona fide "Sen Low" AML cell line model that recapitulates the AML patient-derived phenotype, and ii) primary Sen Low blasts are not readily amenable to efficient transduction/genetic manipulation required for knockdown or knockout experiments. For this reason, and to also maintain translational relevance, we used

chemical inhibition of EZH2 via Tazemetostat (TAZ) treatment to interrogate the consequences of reduced EZH2 activity directly in patient-derived AML blasts. We first examined, at the chromatin level, whether EZH2 inhibition via TAZ remodels immune loci by affecting H3K27me3 levels. Using H3K27me3 Cut&Tag (see Materials and Methods section), we observed a widespread loss of H3K27me3 following TAZ treatment, notably at multiple interferon-related genes (**Panel A**) and across a broad set of HLA loci (**Panel B**), indicating epigenetic de-repression of IFN- and HLA-associated regions upon EZH2 inhibition (see also **response to Reviewer#3** for details). We then asked whether this chromatin remodeling was associated with changes at transcript level. Bulk RNA-seq analysis showed positive enrichment of the IFN gene signatures upon TAZ treatment in Sen Low blast (**Panel C**; NES = 0.9607). Although this analysis did not reach statistical significance, its directionality is consistent with the chromatin-level de-repression observed at interferon-related loci and with the broader immune phenotype induced by TAZ, including increased HLA expression (**Fig. 6F** of the main manuscript) and T cell proliferation (**Fig. 6G** of the main manuscript).
In parallel, Cut&Tag analysis revealed broad H3K27me3 loss across senescence-associated genes following TAZ treatment (**Panel D**). Consistently, GSEA demonstrated strong activation of the p53 pathway (**Panel E**; HALLMARK_P53_PATHWAY NES = 1.488, adjusted p = 0.0001326), together with enrichment of senescence- and SASP-related transcriptional programs (**Figure 6B** of the revised manuscript). However, SA-β-Gal positivity did not show a clear increase upon TAZ treatment, either *ex vivo* in Sen Low blasts (**Panel F**) or in the Sen Low PDX setting (**Panel G**).

These findings support a model in which EZH2 inhibition via TAZ primarily remodels the inflammatory and p53 transcriptional programs of the senescence phenotype in the absence of SA-β-Gal accumulation, a phenotype consistent with previous findings of EZH2 inhibition in pancreatic cancer models (Chibaya L. *et al.,* Nature Cancer, 2022).

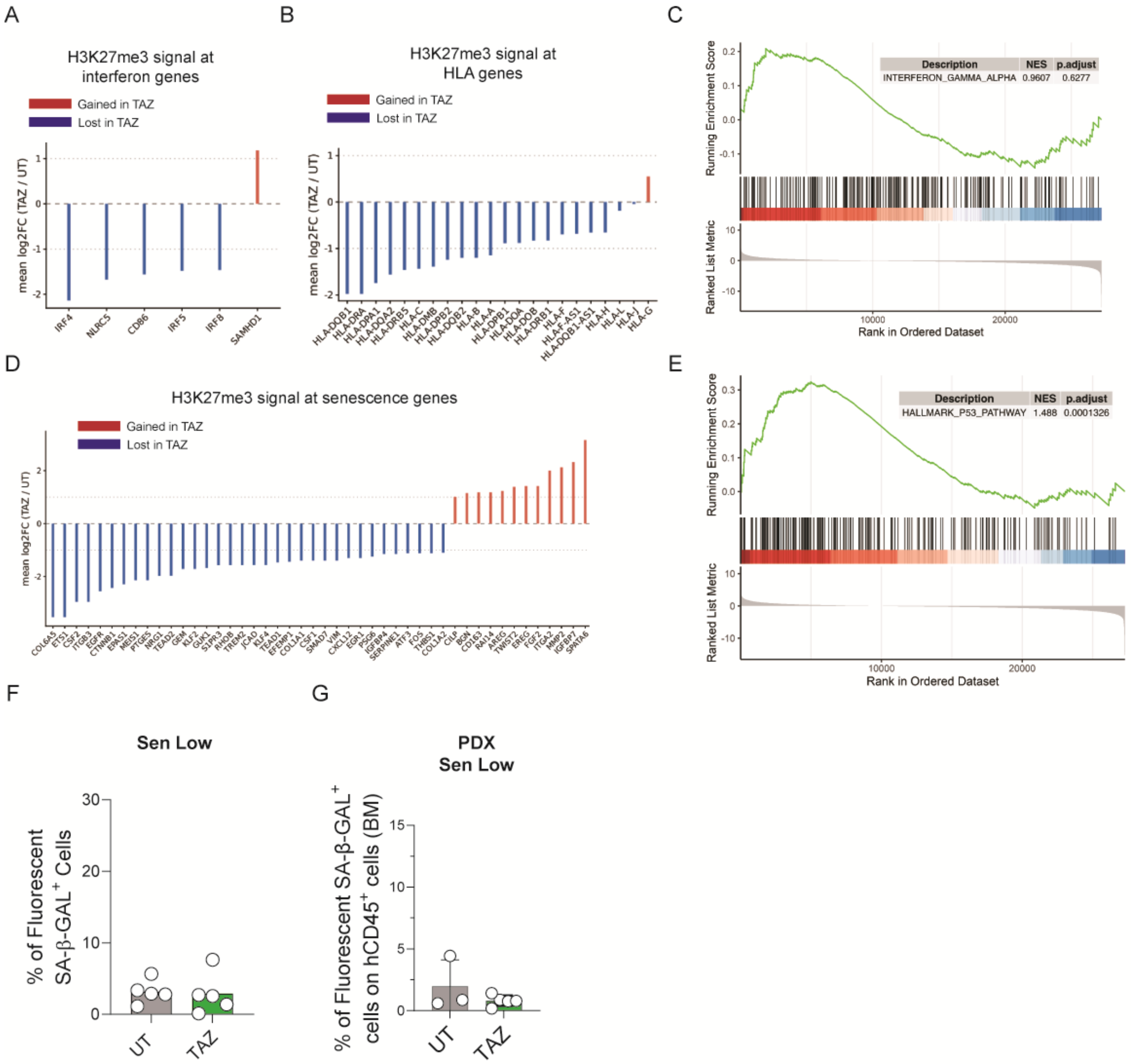

4. The immunopeptidomics data are promising but remain somewhat superficial. I suggest investigating whether the peptides uniquely found in treated "Sen High" samples derive from TAAs, senescence-associated antigens, or neoantigens. Functional validation of one or two candidate peptides in T cell activation assays would be a valuable addition.

We thank the Reviewers for raising this point and for recognizing the potential of our immunopeptidomics dataset. To address their concerns, we expanded our analysis in two complementary directions: (i) annotation of peptide origin and (ii) functional screening in primary AML.

(i) Annotation of peptide origin

To directly address the origin of the ligands presented after AraC treatment, we expanded the annotation of our eluted peptide lists to define tumor-associated antigens (TAAs) and senescence-associated antigens.

We first interrogated peptides mapping to known TAAs across all samples (Sen Low and Sen High) and compared untreated (UT) versus AraC-treated conditions (figure on the right). This analysis showed a clear group-level pattern: Sen Low samples displayed very few TAA-associated peptides and no evident treatment-dependent increase, whereas in Sen High we observed a treatment-associated increase in TAAs in 2 out of 3 samples. We have included this data in new **Fig. 2J**.

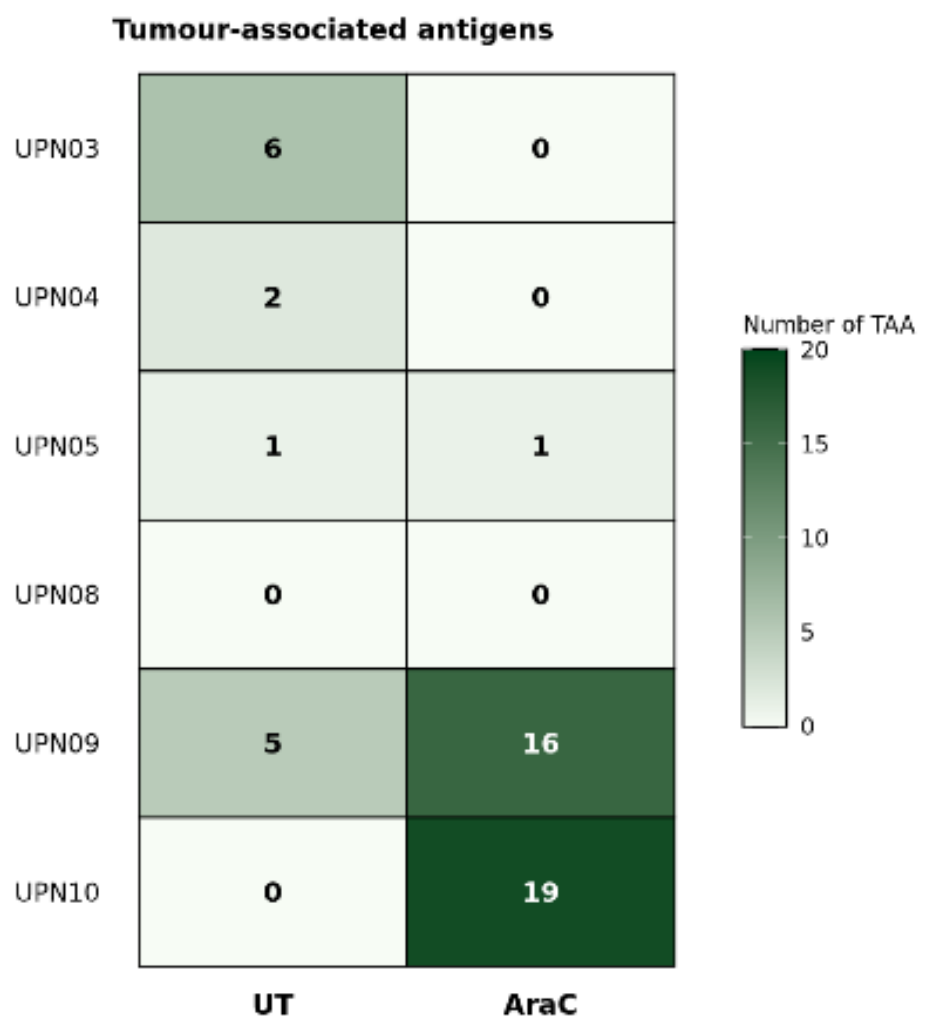

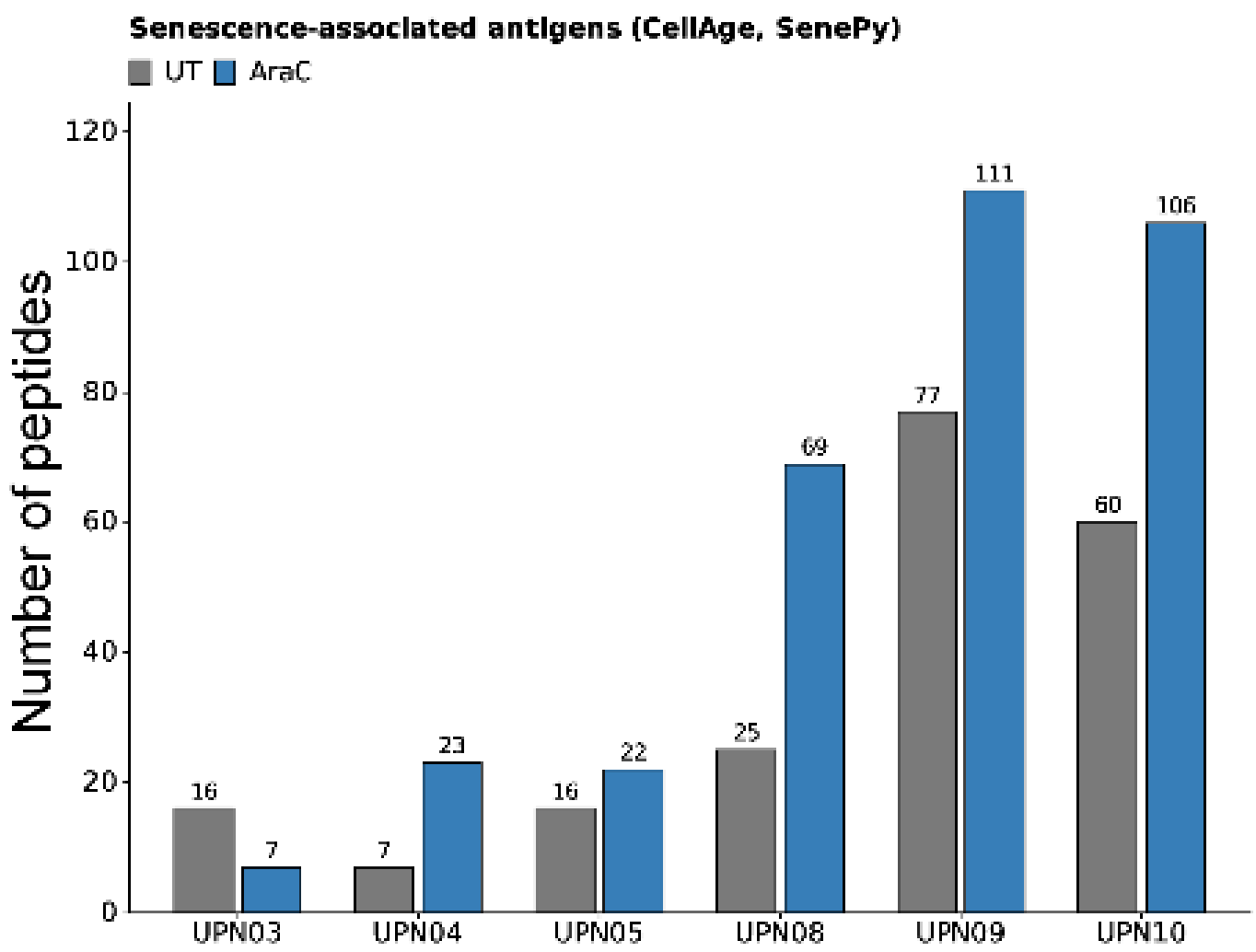

We next focused on senescence-associated antigens by matching peptide source genes to curated senescence signature lists, such as CellAge (Avelar, R. *et al.*, Genome Biol, 2020) and Senepy (Sanborn, M.A. *et al.*, Nature Comm, 2025). As shown in the figure on the left**,** Sen High samples already displayed higher baseline levels of senescence-associated peptides compared with Sen Low samples and showed a further increase upon AraC treatment, reaching substantially higher levels than those observed in Sen Low samples. This supports the conclusion that chemotherapy preferentially enhances the presentation of senescence-associated ligands in Sen High samples. We have included this data in new **Fig. 2K**.

We agree that assessing neoantigens is an important question. However, AML generally has a lower mutational burden compared to solid tumors (Döhner H. *et al.*, Blood., 2024), and definitive neoantigen identification requires matched tumor–normal sequencing (e.g., by whole genome sequencing). Since matched WGS is not available for our cohort, we do not make neoantigen claims from the current dataset and instead focus on treatment-associated ligands supported by senescence/TAA annotation.

(ii) Functional screening in primary AML.

To perform functional validation of our immunopeptidomic analysis, we adopted a more stringent, Sen High-centered peptide selection strategy. This choice was motivated by the fact that Sen High samples exhibit the most consistent AraC-associated immunogenic rewiring, and by the need to prioritize peptides with the highest likelihood of being reproducibly presented and experimentally testable. Starting from the initial number of eluted peptides from Sen High samples, we focused on those identified in at least 2 out of 3 Sen High samples. We selected peptides among these candidates, based on a combination of (i) gene expression, (ii) predicted HLA-I binding, and (iii) literature relevance to senescence and AML biology, yielding a final shortlist of 68 peptides. We then

commissioned de novo synthesis of the complete 68-peptide panel and arranged the candidates into a predefined pooling matrix (8 pools; 16 peptides per pool, see table below).

MATRIX B	pool #	1		2		3		4	
		1	9	18	26	35	43	52	60
	5	2	10	19	27	36	44	53	61
									62
		3	11	20	28	37	45	54	63
	6	4	12	21	29	38	46	55	64
							47		
		5	13	22	30	39	48	56	65
	7	6	14	23	31	40	49	57	66
					32				
		7	15	24	33	41	50	58	67
	8	8	16	25	34	42	51	59	68
			17						

For functional validation, we implemented a stepwise IFN-γ ELISpot screening strategy with stringent positive controls. First, we established a general assay setup using peripheral blood mononuclear cells (PBMCs) from two healthy donors (healthy controls, HC), to evaluate whether the selected candidate peptide pools could induce detectable T-cell reactivity in a non-leukemic donor context.
In this setting, $CD3^+$ T cells were isolated from healthy-donor PBMCs and used as effector cells, while the matched $CD3^-$ fraction was irradiated and used as an autologous antigen-presenting cell compartment for peptide presentation. Assay performance was confirmed by robust responses to phytohemagglutinin (PHA), used as an inducer of polyclonal T-cell activation, and by detectable responses to the CEFT viral epitope pool, comprising CMV-, EBV- and influenza-derived peptides (**Panel A**). In contrast, none of our candidate peptide pools elicited IFN-γ responses above DMSO control in either healthy control (**Panel B**).

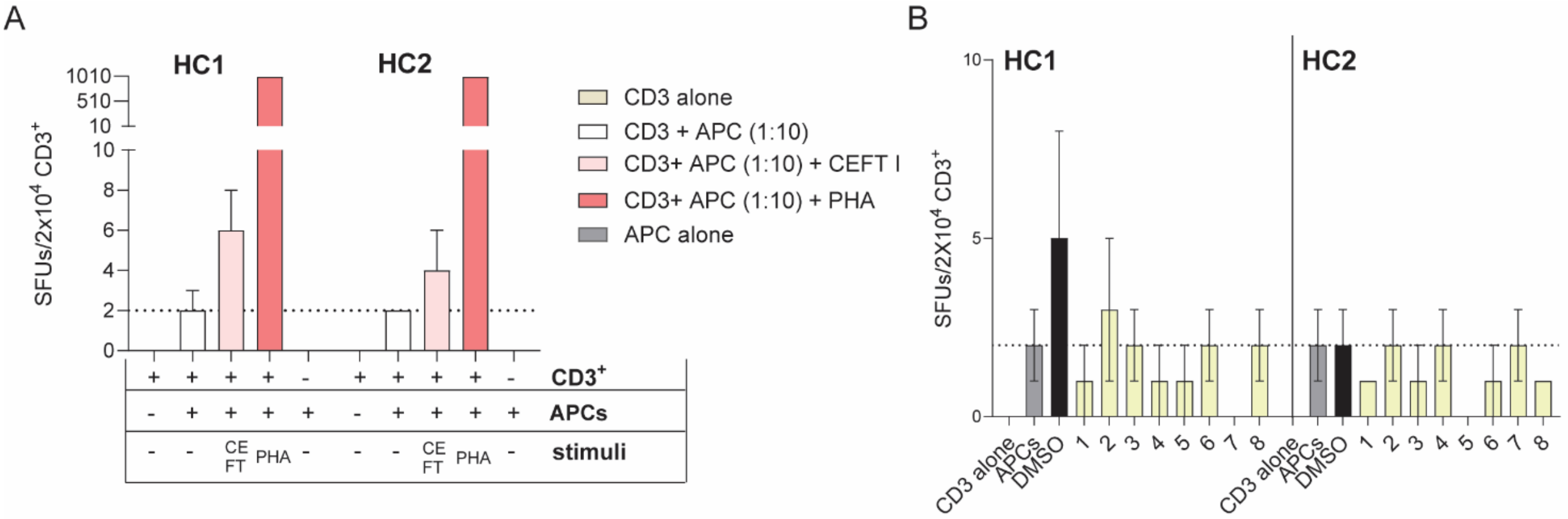

Having established a general assay setup in healthy donors and confirmed the absence of broad non-specific T-cell reactivity against our candidate peptide pools in a non-leukemic context, we next moved to a more disease-relevant autologous setting. Specifically, we co-cultured patient-derived $CD3^+$ T cells with matched AML blasts collected at diagnosis and used as the autologous antigen-presenting/target compartment (n = 2).
Despite strong responses to PHA/CEFT, none of the pools exceeded the ≥1.5× DMSO threshold, consistent with limited leukemia-directed priming and/or reduced functional responsiveness against samples at diagnosis (see figures below).

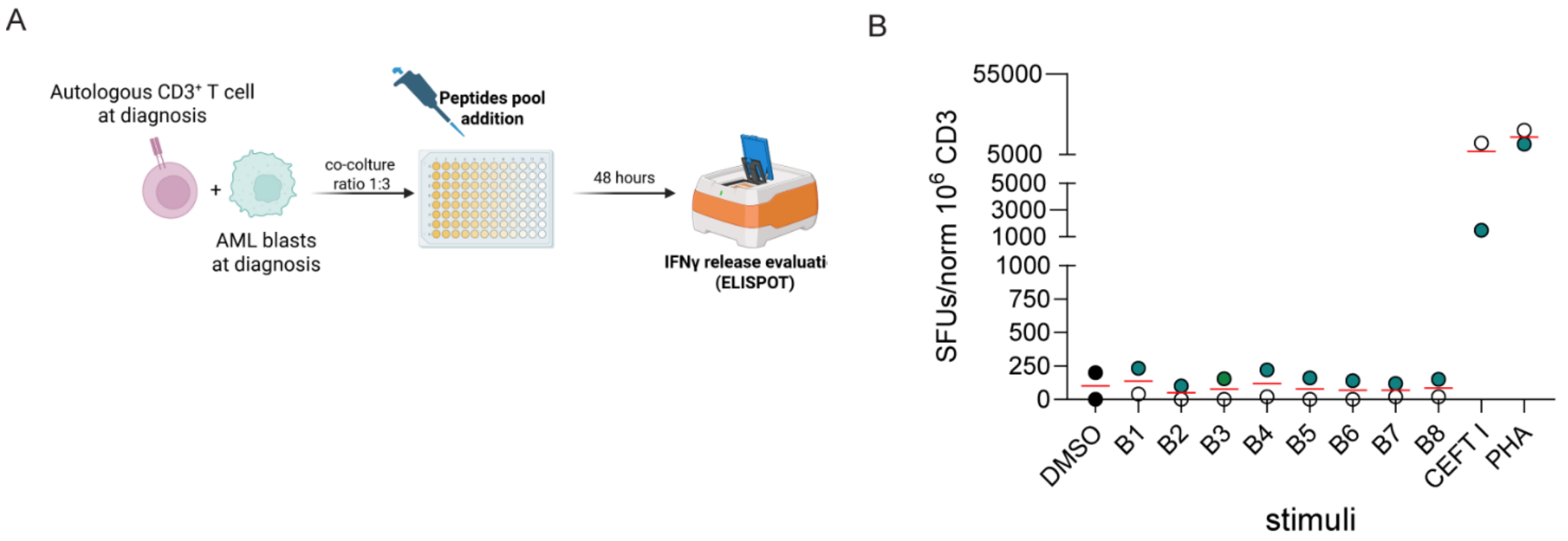

Finally, to maximize the likelihood of detecting antigen-specific responses in our settings, we tested autologous T cells collected 30 days post-chemotherapy, reasoning that these T cells are more likely to have encountered leukemia-associated ligands *in vivo* and may be more reactive against our selected peptides. Notably, although day-30 autologous T cells from 2 AML samples were overall less responsive than T cells at diagnosis (as reflected by reduced ELISpot signals even under PHA or CEFT positive control stimulation), Pool 2 reproducibly elicited a clear increase in IFN-γ spot counts over DMSO in the two AML samples. We therefore focused on Pool 2 as the shared reactive pool across the two patient-derived settings, rather than on pools showing patient-restricted or less consistent increases. Pool 2 approached a ~two-fold increase relative to DMSO and comparable to the effect of CEFT (**Panel A, B** below).

Interestingly, Pool 2 peptides map to factors that are highly consistent with a senescence-linked cellular state and with pathways expected to be engaged during therapy response, including: nuclear architecture and senescence-associated remodeling, proteostasis and protein quality control (VCP; chaperones HSPA8/HSPA1A), RNA/stress-granule biology (G3BP1, SYNCRIP), DNA damage repair (XRCC6), and ER/cell stress and immunogenic remodeling (CALR), alongside proteins implicated in AML/stress signaling and cellular adaptation (SDCBP, S100A13, PKM, AHNAK).

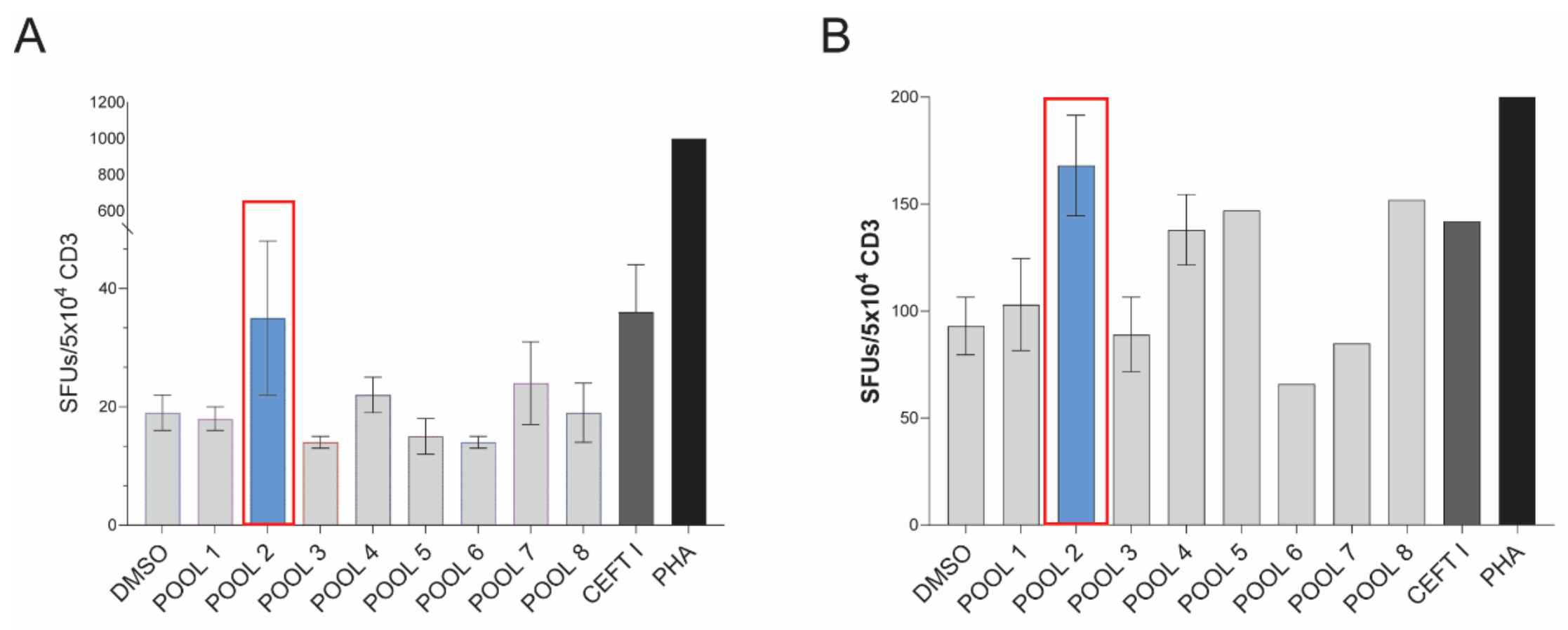

This functional validation is presented for Reviewer only since experiments were performed on a limited number of AML samples. However, we would be happy to include these data in the revised manuscript if the Reviewers and the Editor deem it necessary.

5. Further exploration of the mechanism underlying PRC2 downregulation in "Sen Low" is warranted. Analysis of EZH2, SUZ12, and EED expression, as well as regulators such as ASXL1, could provide insights into the epigenetic profile observed (Fig. 5).

We agree with the Reviewer that clarifying PRC2 regulation across Sen Low and Sen High is important. To address this point, we analyzed our RNA-seq dataset comparing Sen Low vs Sen High (untreated) and found higher transcript levels of most core PRC2 components (e.g. EZH2, EED, RBBP4, RBBP7) in Sen Low samples (see figure on the right).

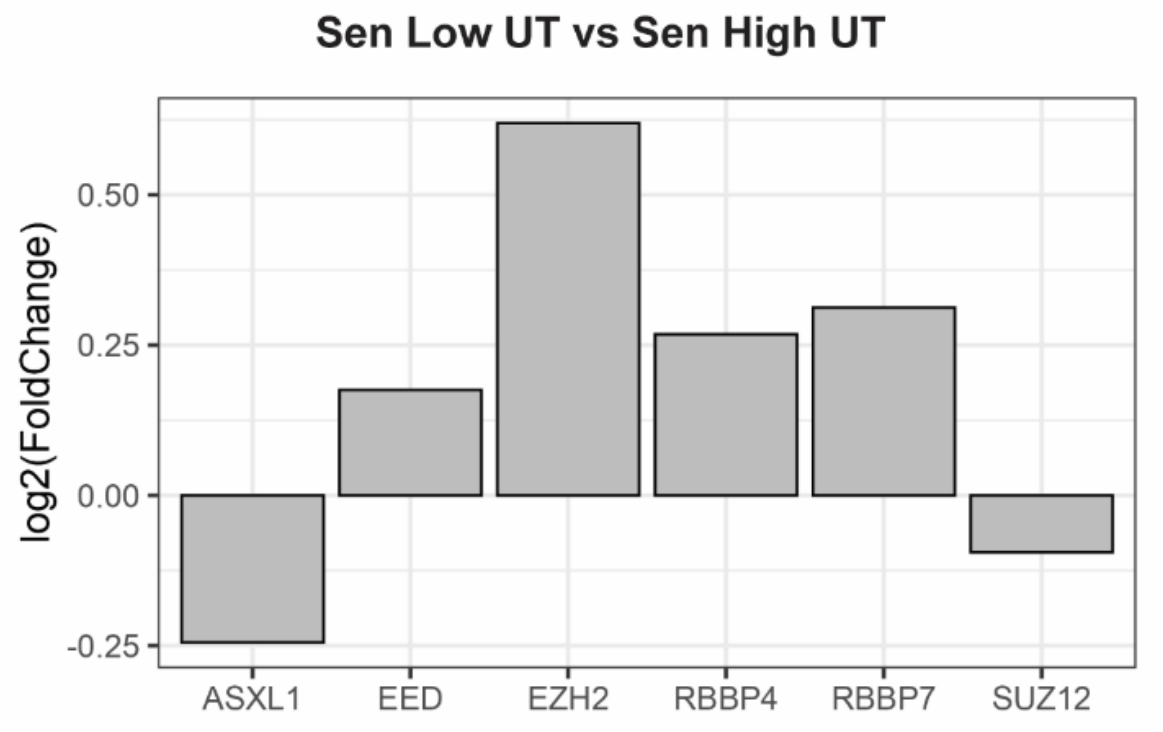

Second, we performed western blot analyses on untreated primary AML samples and observed that Sen Low patients display relatively higher EZH2 protein levels together with increased H3K27me3 (**Panel A, B** on the right), consistent with elevated EZH2 catalytic activity at baseline compared to Sen High.

We have now added these data in new **Fig. 5F** and **Supplementary Fig. 5D**.

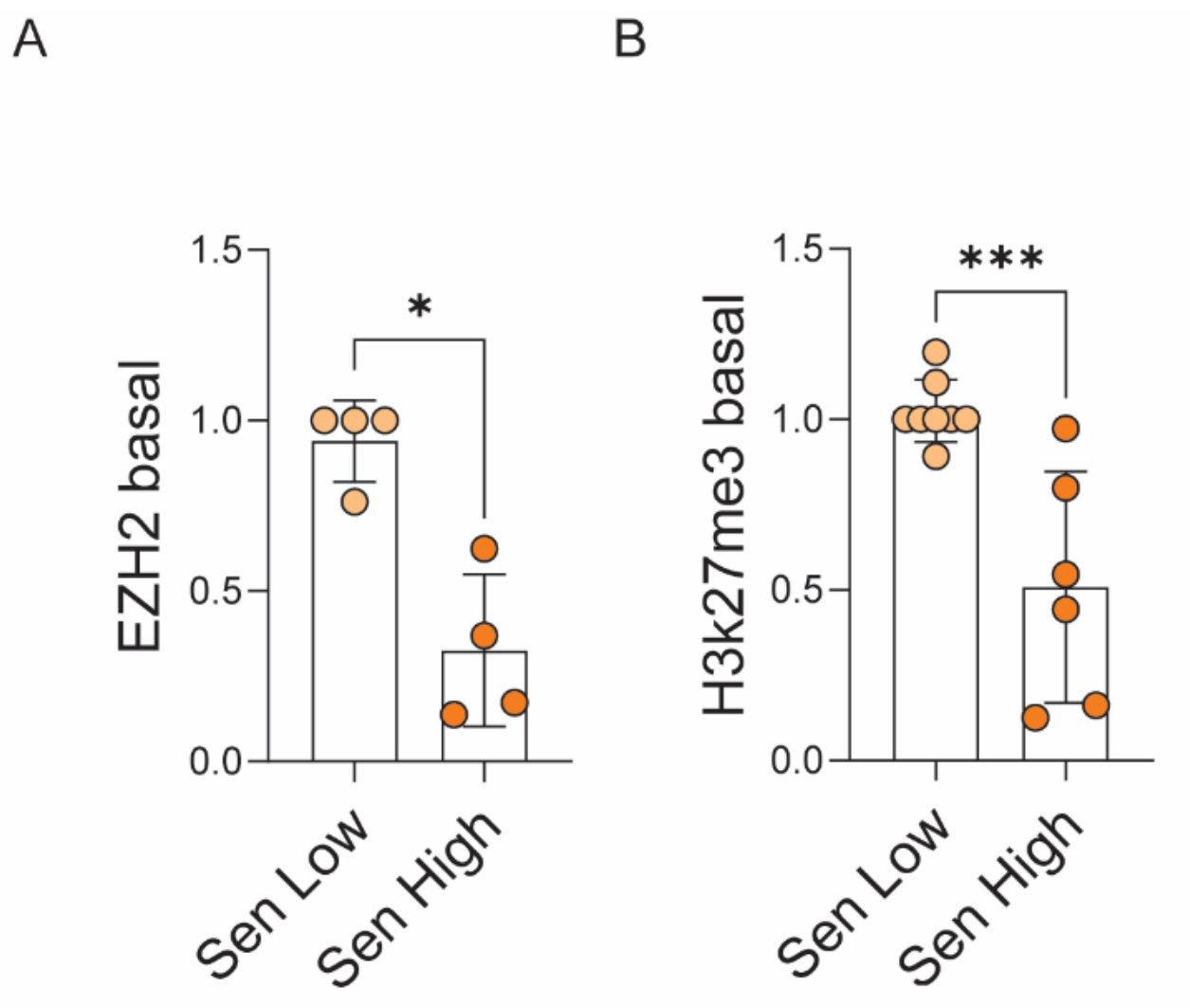

6. The Tazemetostat experiment (Fig. 5F-J) is convincing but lacks an essential control: treatment of “Sen High” samples. This would clarify whether EZH2 inhibition specifically reverts the “Sen Low” phenotype or broadly enhances immunogenicity across all AML samples.

We thank the Reviewer for this important suggestion. As also addressed in our response to **Reviewer#1**, we now provide a side-by-side comparison of HLA levels upon EZH2 inhibition via Tazemetostat (TAZ) treatment in additional Sen Low samples (now up to n = 7 paired samples, UT *vs* TAZ) and in Sen High samples (n = 5 paired samples, UT *vs* TAZ). In Sen Low, the inclusion of additional data confirmed a significant upregulation of HLA class II (HLA-DR$^+$ cells) and HLA class I (HLA-ABC MFI) upon TAZ (paired Wilcoxon test; **Panel A**, below). In contrast, AraC in Sen Low samples did not induce HLA class II and I upregulation (**Fig. 2 C, D** of the main manuscript). In Sen High samples TAZ treatment did not increase HLA class II levels whereas it induced a significant increase in HLA class I (**Panel B**, below). However, AraC treatment in Sen High samples had stronger effects on HLA class II and HLA class I levels compared to TAZ treatment (**Fig. 2 C, D** of the main manuscript).

We have updated the graphs of Sen Low patients in **Fig. 6F**, including the new data on Sen High samples in **Supplementary Fig. 6C** of the manuscript and revised the text accordingly.

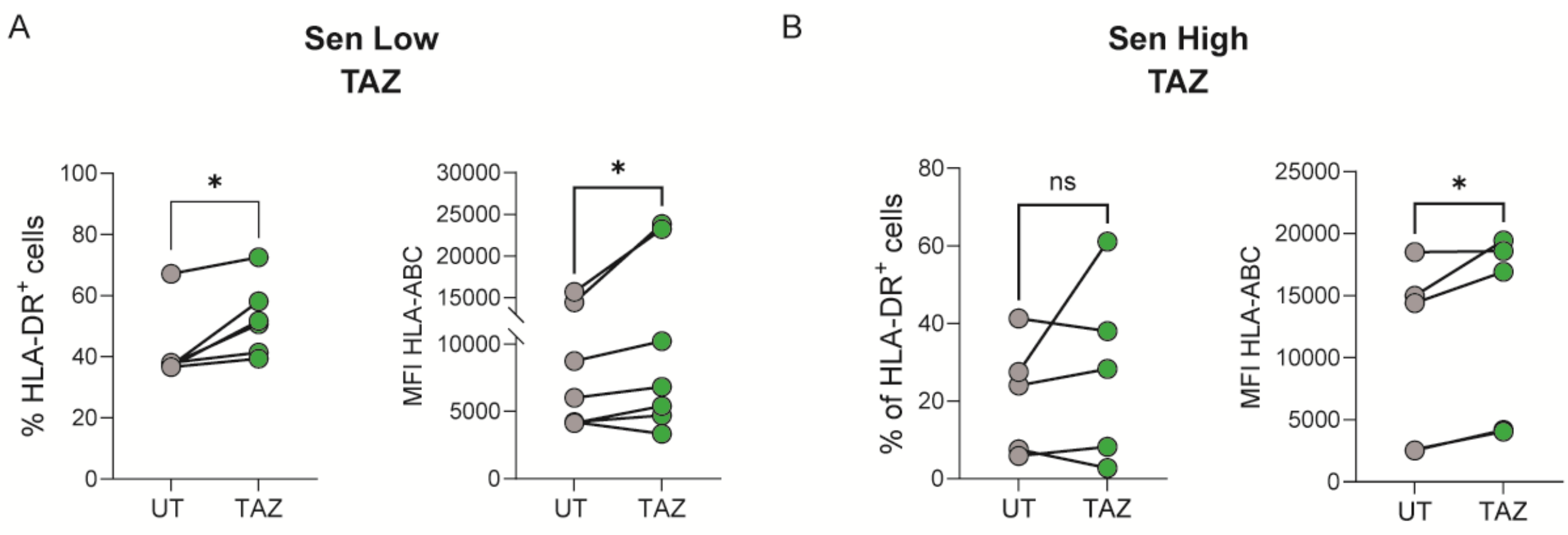

7. It would be useful to expand the discussion of TIS temporal dynamics, as the study focuses on a 5-day window. Public datasets suggest that senescence may be transient; therefore, discussing how this temporal aspect impacts the proposed therapeutic strategy would be valuable.

We agree with the Reviewer that the temporal dynamics of TIS are central for therapeutic translation. Our study was intentionally framed around the early post-chemotherapy response, and this is when we observe the establishment of a senescence program in a subset of AML samples together with immunogenic rewiring. In line with our data, the *ex vivo* AraC-treated scRNA-seq dataset from Duy *et al.*, showed that senescence-enriched clusters displayed increased expression of HLA class I and class II genes, indicating co-occurrence of senescence programs and antigen-presentation features at single-cell resolution. We reason that the early establishment of therapy induced senescence in AML can create an actionable immunogenic window that primes immune recognition and may contribute to long-term benefit through improved control of residual disease (see reference to the clinical TIS outcome in response to point 11 to this Reviewer). However, we cannot rule out the detrimental role of senescent cells on tumor relapse if they persist and/or escape immune-mediated clearance. We have revised the Discussion to frame TIS as a time-dependent double-edged phenomenon and to outline how the transient immunogenic window identified here could be exploited to potentiate immune-based strategies to target senescent cells (including senolytic CAR-T based therapies).

8. To reinforce the claim that T cell activation is specific to leukemia/senescence antigens, I recommend including additional assays such as IFN-γ ELISpot or cytotoxicity assays using expanded T cells against autologous senescent versus non-senescent blasts.

We agree that additional functional readouts can further support the specificity of T cell activation toward leukemia/senescence-associated antigens. To address this point within the constraints of primary material, we strengthened our functional evidence in two ways. We first leveraged our immunopeptidomics-guided functional screening strategy using day-30 post-chemotherapy autologous T cells and patient-derived AML blasts, which provides a biologically relevant context in which T cells have plausibly encountered leukemia/senescence-associated ligands *in vivo* (see response to point 4). In this setting, we detect pool-specific IFN-γ responses above DMSO background (notably Pool 2 in two independent primary AML samples), supporting that the reactivity is not generic but instead compatible with antigen-driven recognition in an autologous leukemia context. As stated above, we would be happy to include these data if Reviewer and Editor deem it necessary.

We next re-analyzed the co-culture endpoints of **Fig. 4A-E** and quantified the residual leukemic fraction (by CD33$^+$ marker) at the end of the assay. This may provide a proxy for cytotoxic control of AML blasts in the same experimental setting. Using this approach, we observed a significant reduction of residual CD33$^+$ blasts in the Sen High condition upon AraC treatment (**Panel A**), whereas no significant changes were detected in the Sen Low condition (**Panel B**). This is in line with the enhanced leukemia eradication following AraC+T cell infusion observed in the Sen High PDX (see **Fig. 4J**) compared to Sen Low (see **Fig. 4O**). We hope this clarifies Reviewer's concerns.

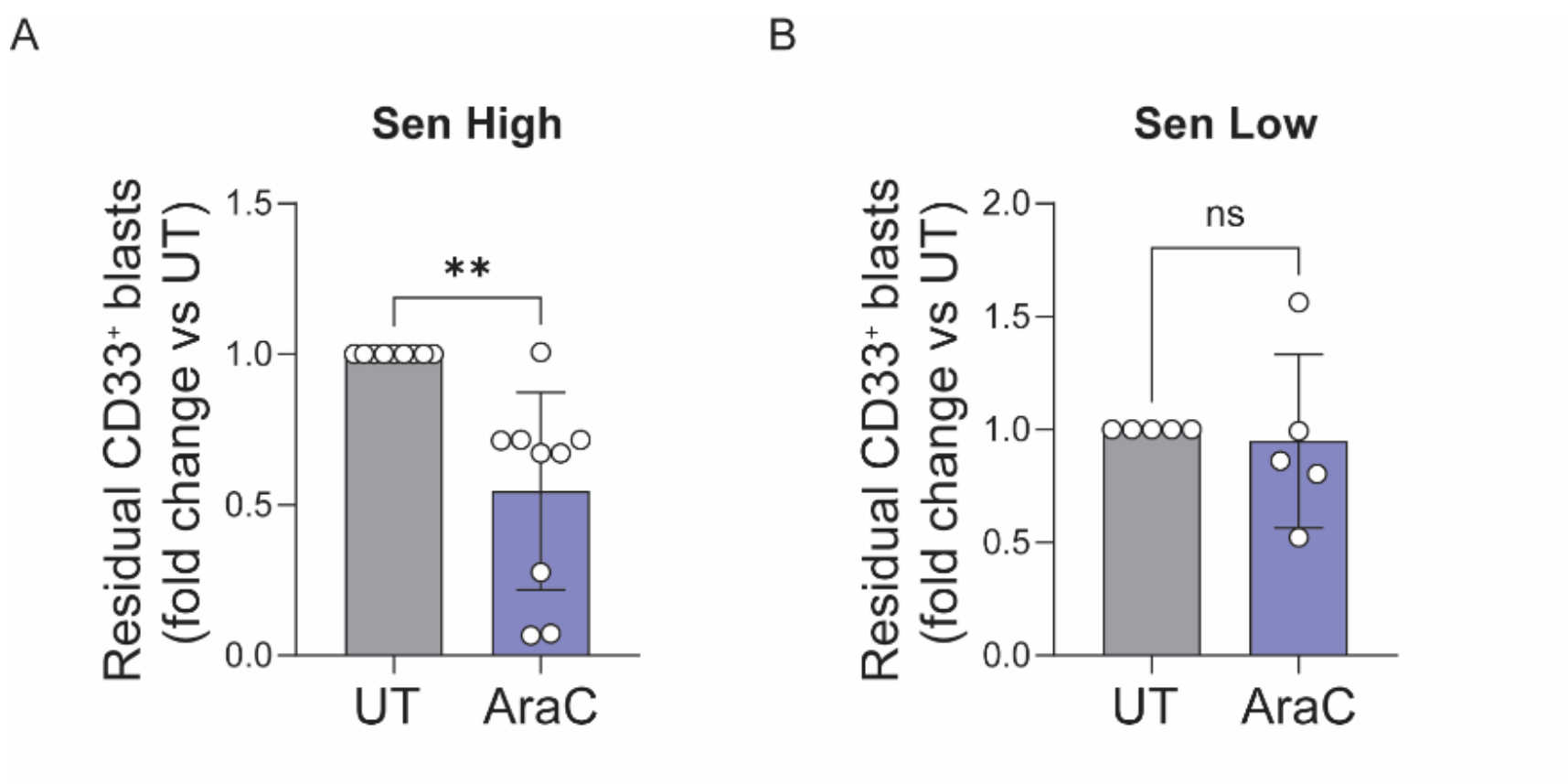

9. In the PDX model (Fig. 4F-K), the analysis centers on human blasts. It would be informative to also assess T cell infiltration and activation status in the bone marrow microenvironment, using immunohistochemistry or flow cytometry for memory and exhaustion markers.

We agree that assessing bone marrow infiltration and the in-situ activation state of T cells would further strengthen the in vivo interpretation. To address this point, we expanded the endpoint flow cytometry analysis in the Sen High PDX model (**Fig. 4F-K**) to include T cell-focused readouts. Specifically, we quantified the frequency of T cells within the human graft in the bone marrow and assessed expression of the activation markers CD25 and CD69 on T cells. This analysis showed detectable T cell engraftment/infiltration in the BM, with higher T cell frequencies in the chemotherapy group (on the right).

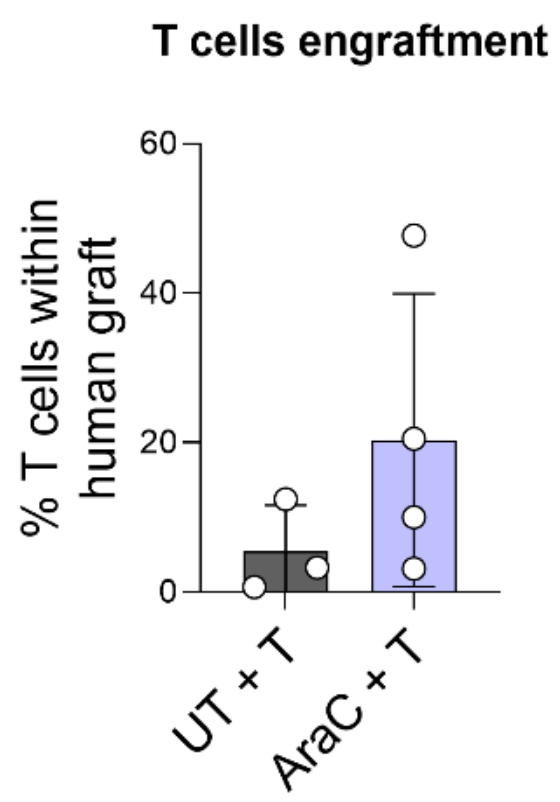

Notably, CD69, a well-established activation marker, increased in the AraC + T group compared with UT + T, whereas CD25 showed no appreciable changes (on le left). While this panel does not provide a full in vivo memory/exhaustion profiling, it offers evidence that transferred autologous T cells can be recovered from the BM and display activation features in the relevant treatment context. We have now included these data in **Supplementary Fig. 4K, L**.

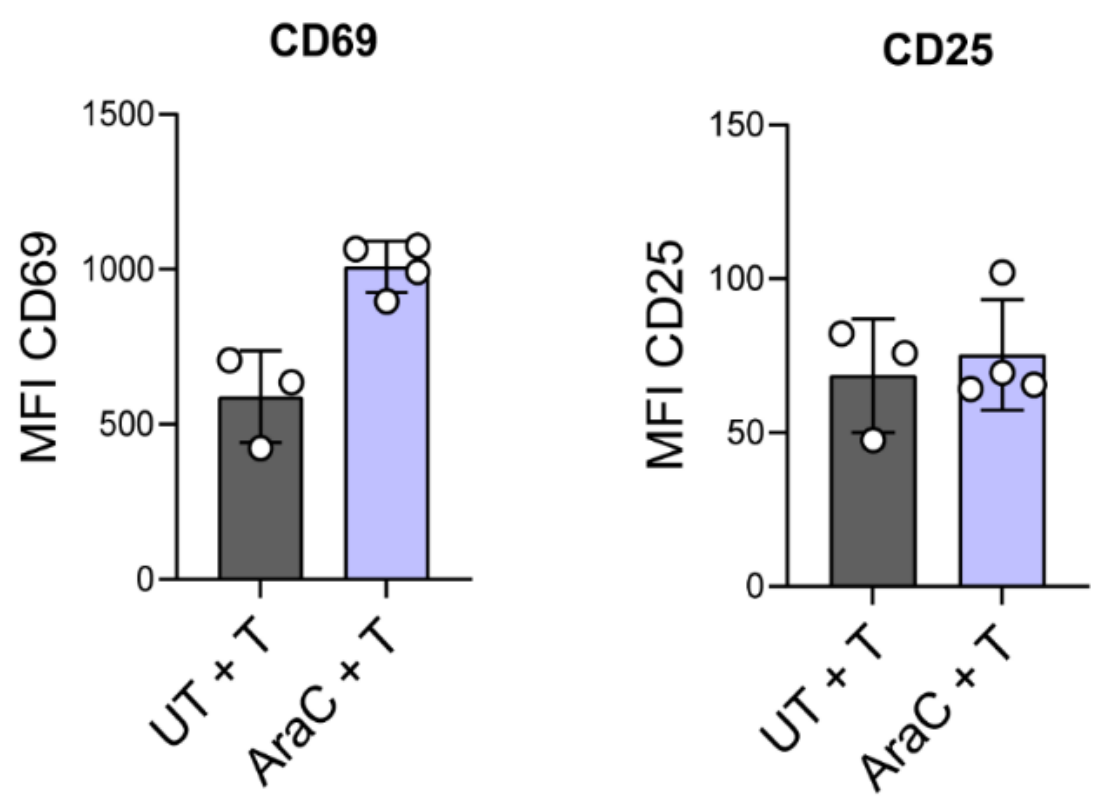

10. The discussion should also acknowledge that EZH2 inhibition may have pleiotropic effects. The improved immunogenicity observed in "Sen Low" samples treated with Tazemetostat may not be exclusively mediated by senescence induction but also by other PRC2-regulated pathways.

We agree with the Reviewer that EZH2/PRC2 inhibition can exert pleiotropic effects. To address this point, we took advantage of our Cut&Tag for H3K27me3 and performed an unbiased pathway enrichment analysis on the loci showing H3K27me3 loss upon Tazemetostat treatment. Notably, the top enriched categories align with the biology emphasized in our model and Interferon gamma response emerged as the leading signature in this analysis (now in **Fig. 6C).**

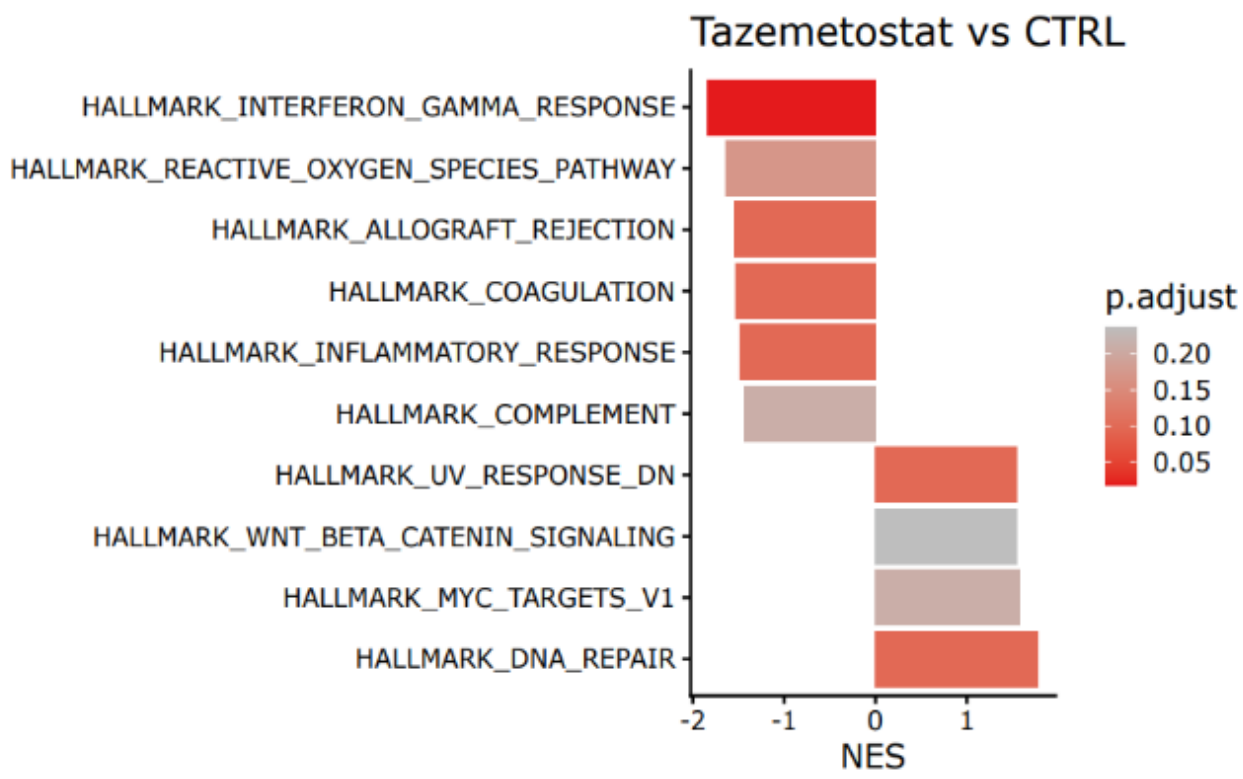

These unbiased results are consistent with loss of H3K27me3 at immune-related regions, including interferon-related genes and HLA-associated loci (see more details in data reported in our last response to **Reviewer#3** and in the revised manuscript in **Figure 6D, E**). These data indicate that Tazemetostat mainly remodels the inflammatory and interferon-related responses as reported in pancreatic cancer models (Chibaya L. *et al.,* Nature Cancer, 2022), rather than establishing a full senescent phenotype (see also lack of SA-β-Gal induction after TAZ in **Supplementary Fig. S6A**).

11. The correlation between "Sen High/Sen Low" status and clinical outcomes is particularly relevant. If feasible, inclusion of Kaplan-Meier analyses or complete remission response data, even in a small cohort, would substantially enhance the translational impact.

To address the translational relevance of our findings, as highlighted for **Reviewer#1** we would like to provide complementary clinical evidence supporting the significance of TIS competence in clinical settings. In our patient cohort (Milan Cohort), Sen High cases, defined by an AraC-induced increase in SA-β-gal positivity measured *ex vivo*, correspond to patients clinically defined as "responders" (residual AML less than 5% at day 14 or 30 post in vivo chemotherapy), whereas Sen Low cases, defined by the absence of such *ex vivo* SA-β-gal induction, correspond to clinically-defined "non-reponders" (residual AML more than 5% at day 14 or 30 post in vivo chemotherapy)(**Panel A**, below). These data are presented in a companion manuscript by C. Schmitt's team currently under revision at *Nature Communications* with potential for back-to-back publication.

In the same clinical work, stratification based on an *ex vivo* SA-β-gal readout (TIS-High vs TIS-Low) in two independent cohort of patient samples (Berlin cohort, n=39, Munich cohort, n=37) correlated with improved outcomes including disease-free survival and overall survival in TIS-high *vs* TIS-Low patients (**Panel B, C,** below).

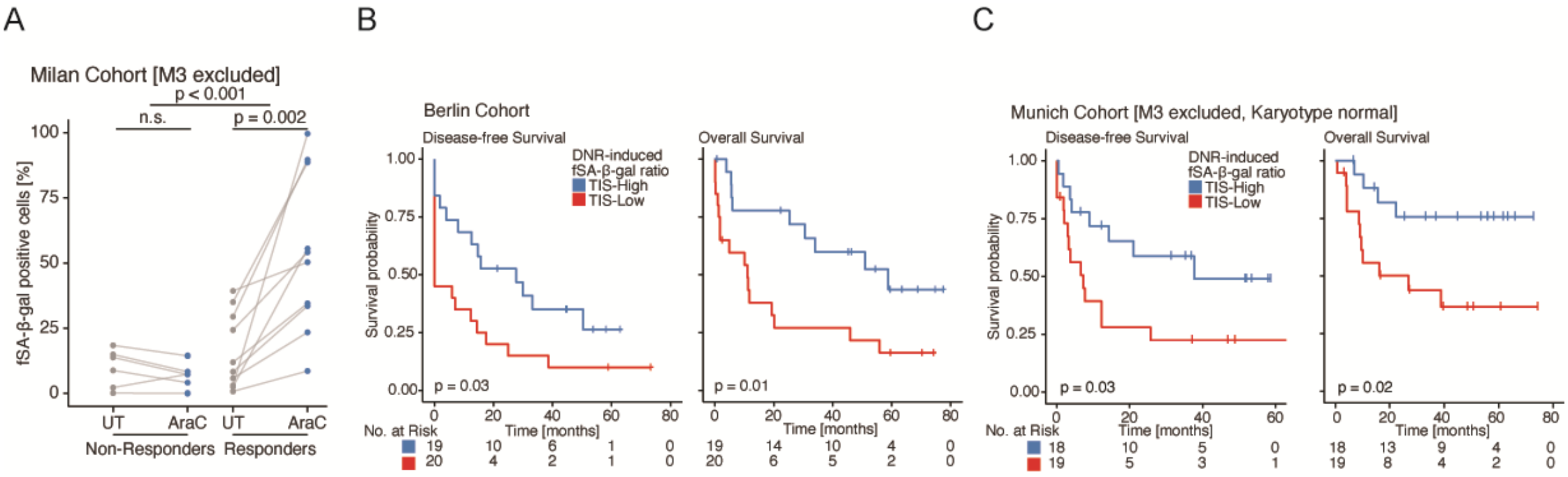

Finally, given the strong immunogenic component emerging from our mechanistic study, we also explored whether TIS competence associates with better outcome after allogeneic-hematopoietic

stem cell transplantation (allo-HSCT), which in AML represents the most established form of immune-mediated disease control.

We combined clinical data from patients across three cohorts (Milan, Berlin, and Munich) who underwent allo-HSCT. Patients who relapsed before transplant were excluded. To ensure consistency across cohorts, the analysis was restricted to cases with normal karyotype, as one contributing cohort (Munich) included only patients with normal karyotype. The final study population comprised 25 patients.

As shown in the figure on the right, TIS-high patients had improved overall survival after allo-HSCT compared with TIS-low patients, with a clear separation of the Kaplan–Meier curves over time and a p value approaching statistical significance (log-rank p = 0.086). We provide these data for Reviewer and Editor only, given the limited sample size after clinical filtering. However, this trend is consistent with the concept that TIS competence associates with a more immunologically controllable disease state and may positively influence outcome in the context of immune-mediated therapies such as allo-HSCT.

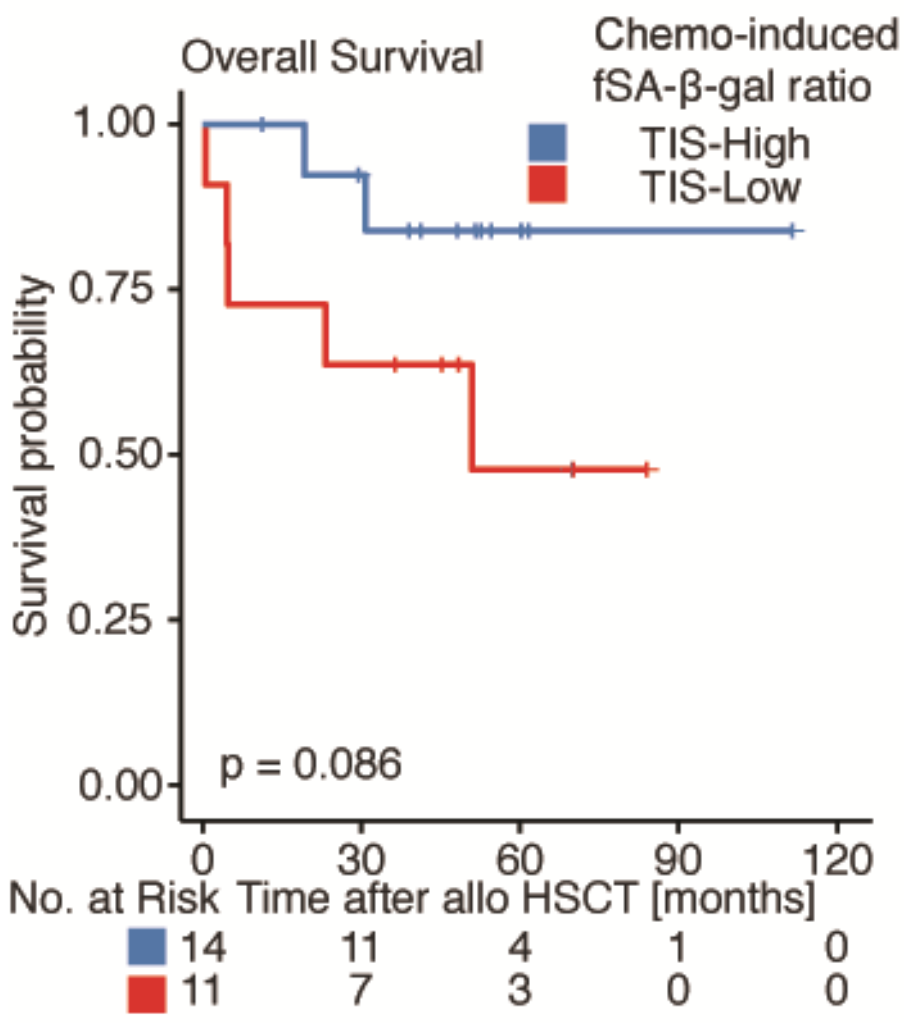

12. Please also justify the choice of the 400 nM AraC dose used *ex vivo* (Fig. 1A) relative to plasma concentrations achieved in patients, and discuss the relevance of the 60 mg/kg dose in the PDX model compared with the standard clinical "7+3" regimen.

We have now clarified the rationale for the 400 nM AraC concentration used *ex vivo* by relating it to clinically reported plasma levels, and we expanded the description of the PDX AraC dosing regimen in the context of standard "7+3" induction, including appropriate literature support and a corrected figure legend. Specifically, the 400 nM AraC concentration was selected empirically based on our dose-response experiments to identify a condition that reproducibly induces senescence-associated features while preserving sufficient viability for downstream phenotyping and functional assays. In the clinics, 400 nM (equal to 0.4 μM) is in the same order of magnitude as the steady-state plasma concentrations reported during standard induction therapy based on continuous infusion cytarabine (Fleming, R.A., *et al.,* Cancer Chemother. Pharmacol., 1995).

In our PDX experiments, mice were treated for five consecutive days (every 24 h) with i.p. injections of cytarabine at 100 mg/kg. This schedule is widely used as an induction-like regimen in AML mouse studies; for example, Zuber *et al*. established a chemotherapy regimen involving cytarabine (100 mg/kg/day ×5) (in combination with an anthracycline) "which mimics the standard induction therapy used to treat AML in human patients" (Becker P. *et al.*, British Journal of Haematology, 2011).

We acknowledge that preclinical dosing cannot replicate all aspects of clinical "7+3" (e.g., infusion kinetics, supportive care, and anthracycline component). In our NSG PDX setting, we administered AraC alone because SCID-based NSG models can show marked sensitivity to chemotherapy in combination with anthracyclines, with increased toxicity and mortality depending on conditioning and regimen intensity. Notably, AraC-only regimens are also commonly used in NSG AML models to study early response biology, including physiologically relevant schedules in the context of chemotherapy-induced senescence/resilience (Duy C. *et al.*, Cancer Discovery, 2021).

On a separate note, we noted that the AraC dose was incorrectly reported in the legend of **Fig. 4F** of the manuscript. We apologize for this mistake, the correct dose is 100 mg/kg, as stated in the Methods, and we have now updated the figure accordingly.

13. I recommend including a more comprehensive clinical data table in the main manuscript, covering cytogenetic/molecular risk (ELN), blast counts, and post-induction outcomes, to allow readers a better understanding of the study cohort.

We agree that a more comprehensive clinical table will improve cohort interpretability. We have therefore updated the table to include cytogenetic/molecular risk classification according to ELN 2022 (as assigned by our clinical collaborators) and key post-induction outcomes. In addition, we now report whether patients underwent hematopoietic stem cell transplantation (HSCT) and whether they experienced relapse after treatment, when available. The revised table (Supplementary Data 1) has been incorporated into the main manuscript.

14. Finally, some figure legends and descriptions require clarification. In Fig. 1C, the exact meaning of the "Interaction-Log2FC" axis should be explained; in Fig. 2J, the enrichment analysis of peptides should be described more clearly in the main text; and, in general, the statistical tests used in the LME models should be indicated more explicitly either in the figure legends or results section.

We have addressed these clarity issues in the revised manuscript: (i) we now explicitly define the "Interaction–Log2FC" axis in the **Fig. 1C** legend (and briefly in the Results section), (ii) we expanded the main-text description of the peptide enrichment analysis shown in **Fig. 2L**, and (iii) significance annotations shown in the Figures correspond to post hoc pairwise comparisons performed after estimation of the Linear Mixed-Effects (LME) models. We have revised the Figure legends accordingly to explicitly include this information. Moreover, Supplementary Table 1 reports the complete LME model output, including fixed-effect estimates, standard errors, and associated p-values, together with details on outcome transformations, if applied, to fulfill model assumptions and the complete results of the post hoc pairwise comparisons (Pinheiro and Bates, 2000). We hope to have provided a clearer description of the LME analyses and tests performed.

Reviewer#3

The manuscript "Therapy-Induced Senescence Promotes Immunogenicity in Acute Myeloid Leukemia Blasts through loss of PRC2-Mediated Gene Regulation" from Gilioli, Fusco and colleagues show that upon treatment a proportion of the AML patients respond with therapy-induced cellular senescence (TIS) that is characterized by senescence-associated secretory phenotype (SASP). Furthermore, they show evidence that this phenotype provides with beneficial for the cancer clearance immunogenicity by HLA-I and HLA-II upregulation and subsequent CD8+ and CD4+ cell response. Finally, in the proportion of patients where TIS is not induced the authors suggest the existence of an underlying epigenetic vulnerability by the accumulation of negative chromatin marks such as H3K27me3 that may further be exploited in novel treatment approaches against AML. The study is thorough and well-build, and the manuscript is well-written with very clear graphical presentation.
I find the work of Gilioli, Fusco and colleagues to be of significance to the AML field where to the best of my understanding no previous reports have been published about the utility of TIS in particularly AML. I find the work also of interest to the wider cancer haematology and related fields.

We thank the Reviewer for the thoughtful comments and positive evaluation. We appreciate the assessment that the work is well executed and that our conclusions and claims are well substantiated. We also value positive feedback on the manuscript's clarity and graphical presentation, and the Reviewer's emphasis on the relevance of these findings to the AML field and broader cancer research field.

Previous authors have defined the therapy-inducible SA-β-gal-based senescence responsiveness as either senescence-competent or -incompetent for what Gilioli, Fusco and

colleagues define as Sen High and Sen Low patients/response, respectively (Belenki D, et al. Nat Commun. 2025 Mar 31;16(1):3079. doi: 10.1038/s41467-025-57429-x. PMID: 40159497; PMCID: PMC11955568.). As both groups ultimately strive to establish the importance of the same phenomenon, I would suggest it is beneficial to adhere to the same terminology especially since Gilioli, Fusco and colleagues do use the term "senescence competence" when they discuss their data.

We agree that consistent terminology improves clarity and cross-study comparison. We are aware of the "senescence-competent/incompetent" nomenclature used in prior work and in a companion manuscript from our clinical collaborators currently under invited revision at *Nature Communications*, for a potential back-to-back publication. We decided to stick to the Sen High/Sen Low terminology throughout the manuscript, including the Discussion section. Nonetheless, If the Editor prefers full harmonization with the "senescence-competent/incompetent" terms, particularly in the context of a coordinated back-to-back publication with the companion study, we would be happy to adopt that terminology across the manuscript.

The work is well-executed and presents well-substantiated conclusions and claims. My main concerns with the study remain the balance of the double-sided nature of TIS and SASP in cancer and whether the phenomena may also negatively affect the long-term clearance of AML cells. Would it be possible for the authors to deepen their analysis and discussion on the matter?

We agree with the Reviewer that TIS/SASP is a time-dependent double-edged phenomenon: early senescence can enhance immune visibility, whereas persistent senescent cells and chronic SASP may be detrimental (Xiao S. *et al*., Front. Oncol., 2021; Colucci M, *et al.*, Cancer Cell, 2025). We would like to further highlight that our study focuses on the early treatment-response window and therefore does not experimentally address long-term persistence or relapse dynamics, and we avoid claims of durable disease control driven by TIS. Following this Reviewer suggestion, we now expanded the Discussion to summarize the potential long-term consequences of persistent TIS/SASP in AML and to outline how the transient immunogenic window identified here could be exploited to potentiate immune-based strategies to target senescent cells (including senolytic CAR-T based therapies).

Furthermore, the authors state that "neither the mutational profile nor the karyotypic status explained the senescence-based stratification of our cohort" but this at present is not entirely convincing because of the small size of the cohort. If possible, the authors should increase their cohort and provide cytogenetics for those patients who are currently lacking.

In our cohort (n = 21), we examined the available cytogenetic and mutational annotations and did not observe an obvious enrichment of Sen High or Sen Low cases within specific cytogenetic categories or recurrent mutations. Thus, within our dataset, the senescence-based stratification is not readily explained by mutational or cytogenetic profile and is most consistent with the epigenetic state highlighted by our mechanistic data (PRC2/EZH2 axis), which functionally modulates senescence-associated immunogenic rewiring. Regarding missing cytogenetics in our cohort, conventional karyotyping was generated as part of routine diagnostic/biobanking workflows at the time of sample collection; for cases where these data were not performed or not available at that time, they cannot be reliably reconstructed retrospectively from stored material. We now explicitly indicate missing values in the updated table (Supplementary Data 1).

To provide additional context beyond our sample size, we also referred to analyses performed and presented in the accompanying manuscript from the team of C. Schmitt based on a larger independent cohort of AML samples. In that dataset, TIS competence showed significant differences across ELN risk groups and karyotype categories (e.g., higher TIS competence in favorable-risk *vs*

adverse-risk, and in normal *vs* aberrant karyotype; see below), and it also associates with recurrent AML mutations (e.g., higher TIS competence in *NPM1* and *DNMT3A^R882*-mutant cases, while *SRSF2*-mutant cases show a weaker/negative association; see below). These results are provided here for Reviewer's consideration.

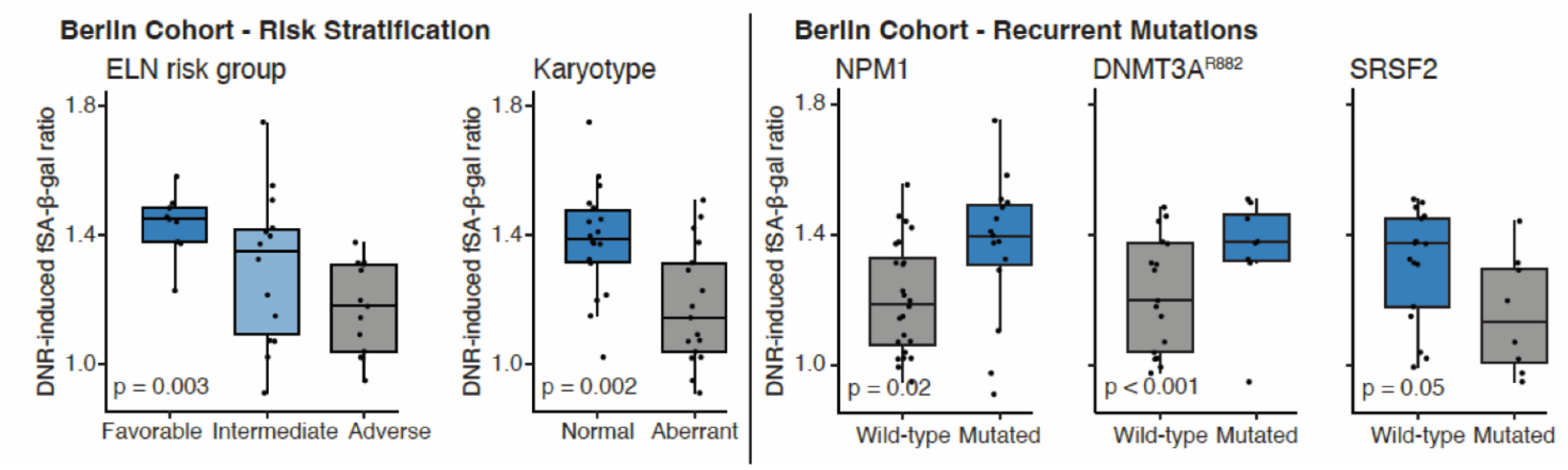

Given these data, we mitigated the claim of non-association between mutations/karyotype and TIS by stating: "Mutational and cytogenetic features did not reveal an obvious segregation according to senescence status, although the cohort size does not allow formal exclusion of genotype-associated effects"

Is it possible that younger (female?) patients are more likely to have senescence-competent AML? If the authors increase their patent pool, they could include appropriate statistics on the effect of age and gender distribution? The authors should at least discuss the possible implications.

We agree that sex- and age-related effects could, in principle, influence treatment responses and therefore warrant further consideration.

Gender. In our cohort, 5 out of 6 patients in the Sen Low group are male, while a more balanced distribution between female and males is observed in the Sen High group (see revised Supplementary Data 1, **Panel A below**). However, to increase sample size we evaluated the impact of gender on senescence establishment in the Berlin cohort (n= 39) of the accompanying manuscript from C. Schmitt lab and did not find a gender-dependent effect on senescence establishment (**Panel B**, below).

Age. In our cohort, patients span a broad age range (30-76 years), and Sen Low (median age 62.83) and Sen High (median age 54.80) samples are distributed across this range without an apparent enrichment in younger patients (**Panel C**, below). Likewise, no significant differences between TIS-Low (median age 68.5) and TIS-High (median age 63) were reported in terms of age range in the Berlin cohort (**Panel D**, below).

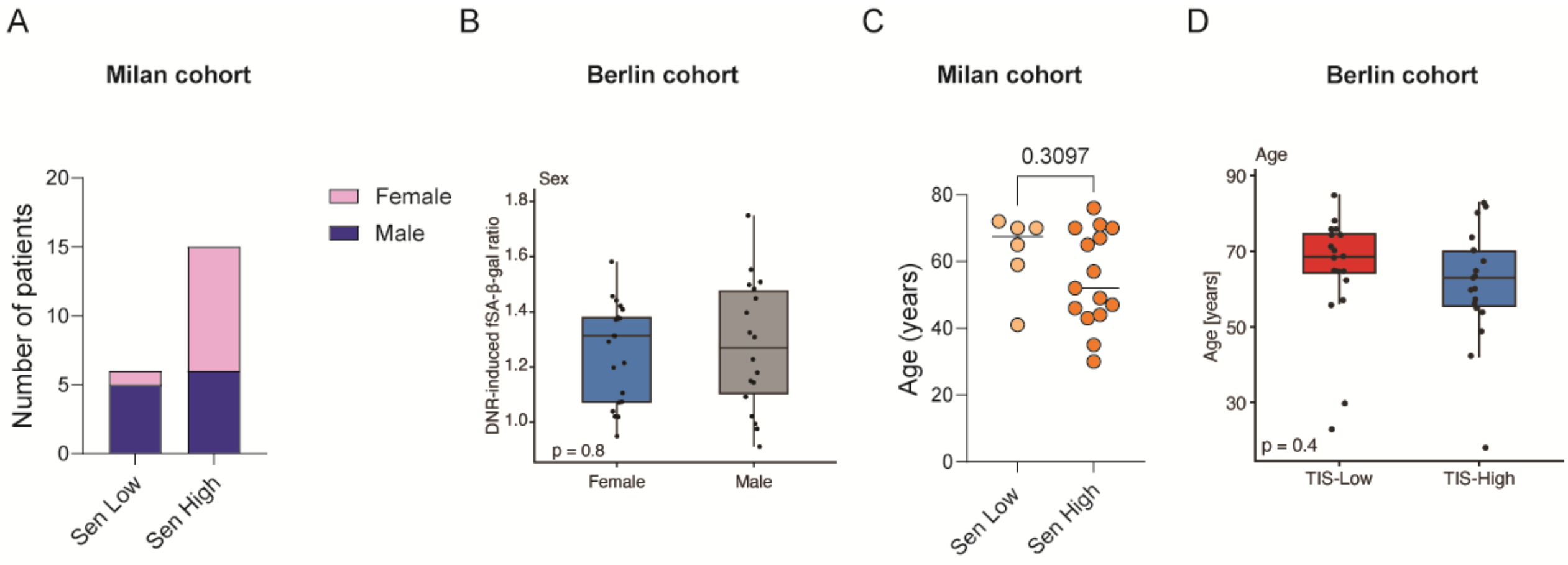

The authors state "Sen Low patients present with inferior long-term outcome to treatment (R. Di Micco and C. Schmitt, personal communications)". This is potentially very interesting and thus should not be provided without the support of the actual data that can be evaluated. The authors must provide the data or remove any personal communication statements.

We thank the Reviewer for this suggestion. We have therefore removed the personal communication phrasing from the revised manuscript discussion and reworded the text accordingly. For transparency, we provide here the data that motivated the statement. In our patient cohort (Milan Cohort), Sen High cases, defined by an AraC-induced increase in SA-β-Gal positivity measured *ex vivo*, correspond to patients clinically defined as "responders" (residual AML less than 5% at day 14 or 30 post *in vivo* chemotherapy), whereas Sen Low cases, defined by the absence of such *ex vivo* SA-β-Gal induction, correspond to clinically-defined "non-responders" (residual AML more than 5% at day 14 or 30 post *in vivo* chemotherapy) (**Panel A**, below). These data are presented in a companion manuscript by C. Schmitt's team currently under revision at *Nature Communications* with potential for back-to-back publication.

In the same clinical work, stratification based on an *ex vivo* SA-β-gal readout (TIS-High vs TIS-Low) in two independent cohort of patient samples (Berlin cohort, n=39, Munich cohort, n=37) correlated with improved outcomes including disease-free survival and overall survival in TIS-high *vs* TIS-Low patients (**Panel B, C,** below).

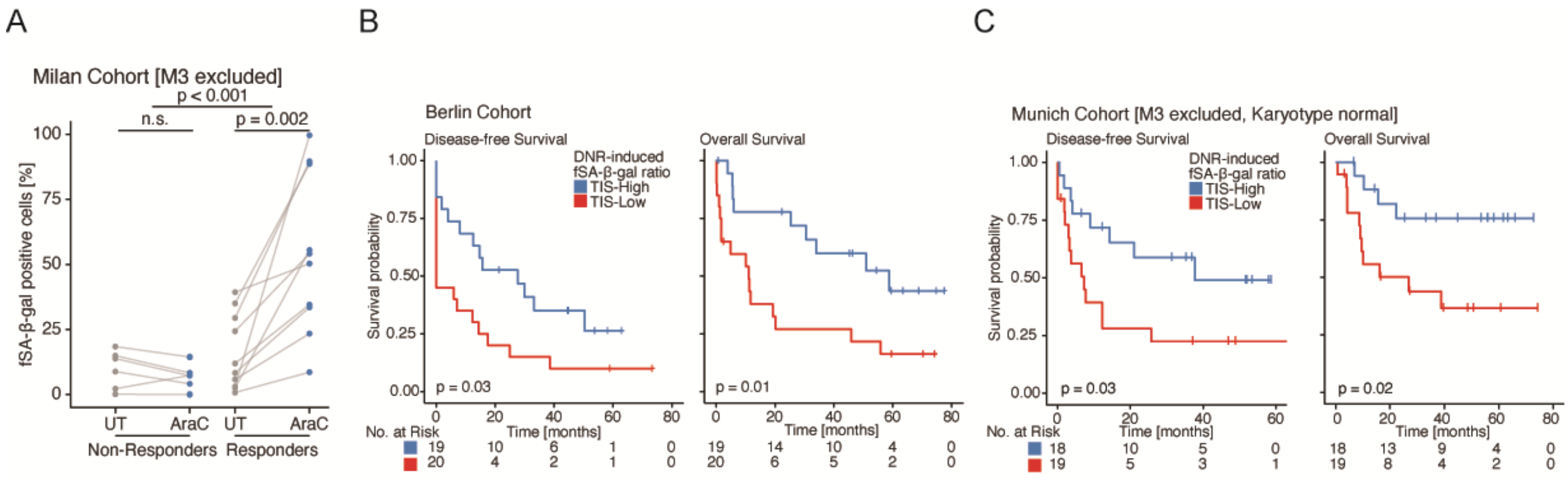

Finally, given the strong immunogenic component emerging from our mechanistic study, we also explored whether TIS competence associates with better outcome after allogeneic-hematopoietic stem cell transplantation (allo-HSCT), which in AML represents the most established form of immune-mediated disease control.

We combined clinical data from patients across three cohorts (Milan, Berlin, and Munich) who underwent allo-HSCT. Patients who relapsed before transplant were excluded. To ensure consistency across cohorts, the analysis was restricted to cases with normal karyotype, as one contributing cohort (Munich) included only patients with normal karyotype. The final study population comprised 25 patients.

As shown in the figure on the right, TIS-high patients had improved overall survival after allo-HSCT compared with TIS-low patients, with a clear separation of the Kaplan–Meier curves over time and a p value approaching statistical significance (log-rank p = 0.086). We provide these data for Reviewer and Editor only, given the limited sample size after clinical filtering. However, this trend is consistent with the concept that TIS competence associates with a more immunologically controllable disease state and may positively influence outcome in the context of immune-mediated therapies such as allo-HSCT.

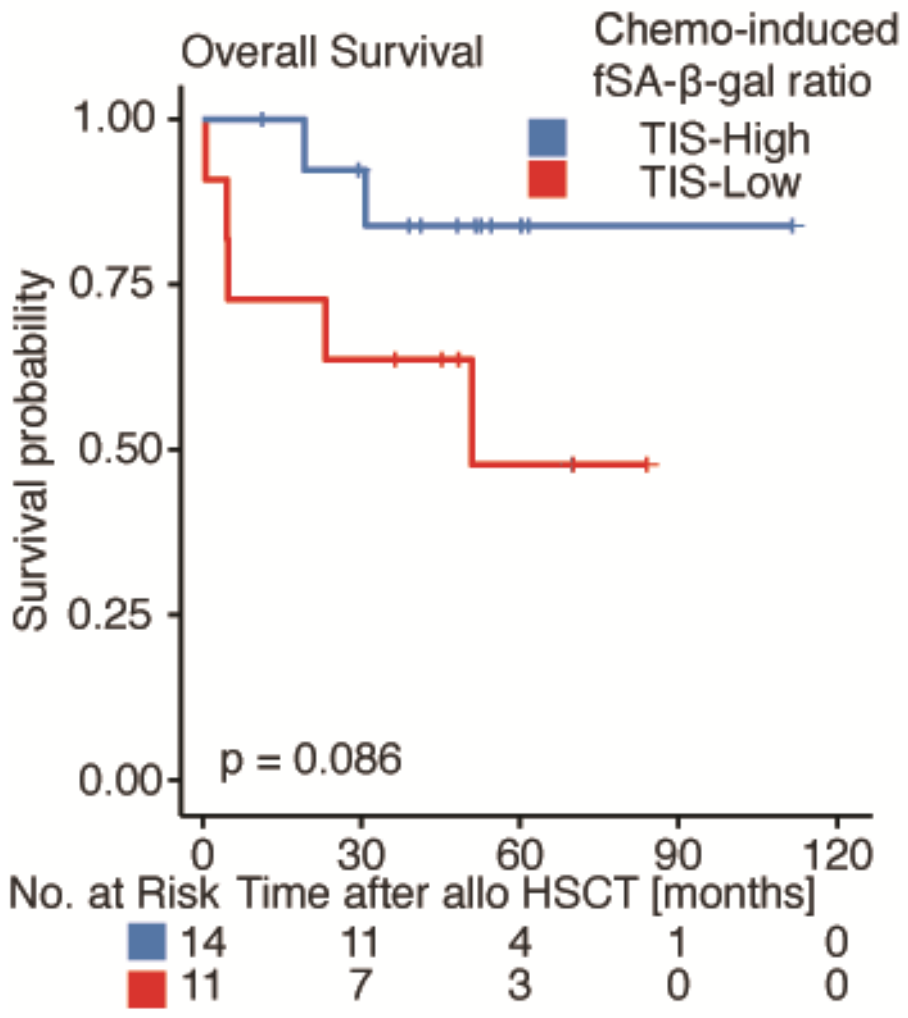

It would be of great interest if the authors provide in vivo validation of tumour clearance after co-culture of TAZ-treated AML blasts from Sen Low samples in the humanized NSG mouse model.

and attempted to perform the *in vivo* validation of immune-mediated leukemia clearance following EZH2-inhibition via Tazemetostat. As also shown in our response to **Reviewer#1**, we transplanted NSG mice with patient-derived AML blasts from a Sen Low sample and, after confirming leukemia engraftment by bone marrow aspirates, we administered the EZH2 inhibitor TAZ (300 mg/kg, oral gavage, daily for 7 days, see **Panel A** below). At day 8 post-treatment we observed a clear reduction of global H3K27me3 levels in leukemic cells from TAZ-treated animals compared to controls (**Panels B, C**, below). TAZ treatment did not reduce leukemia burden (**Panel D**, below), but it induced a significant increase in HLA class I expression (**Panel E**, below), consistent with its immune-priming effects. In a group of TAZ-treated animals we subsequently administered patient-matched autologous T cells, previously expanded *in vitro*. However, in this experiment, due to limited availability of the autologous T-cell product, we performed only one i.v. infusion rather than two as in previously described *in vivo* experiments. At endpoint, we observed a trend toward reduced leukemia burden in the TAZ + autologous T-cell condition compared to TAZ alone (**Panel F**, below). Importantly, this trend emerges despite a reduced T-cell dosing intensity, suggesting that EZH2 inhibition can sensitize Sen Low AML to immune-mediated control; a full two-T cell infusion schedule would be expected to further strengthen this effect. We have now included these data in **Figure 6H-L** of the manuscript and added a brief section in the Discussion explicitly acknowledging the limitations of this *in vivo* setting and outlining it as a key consideration for future longitudinal studies.

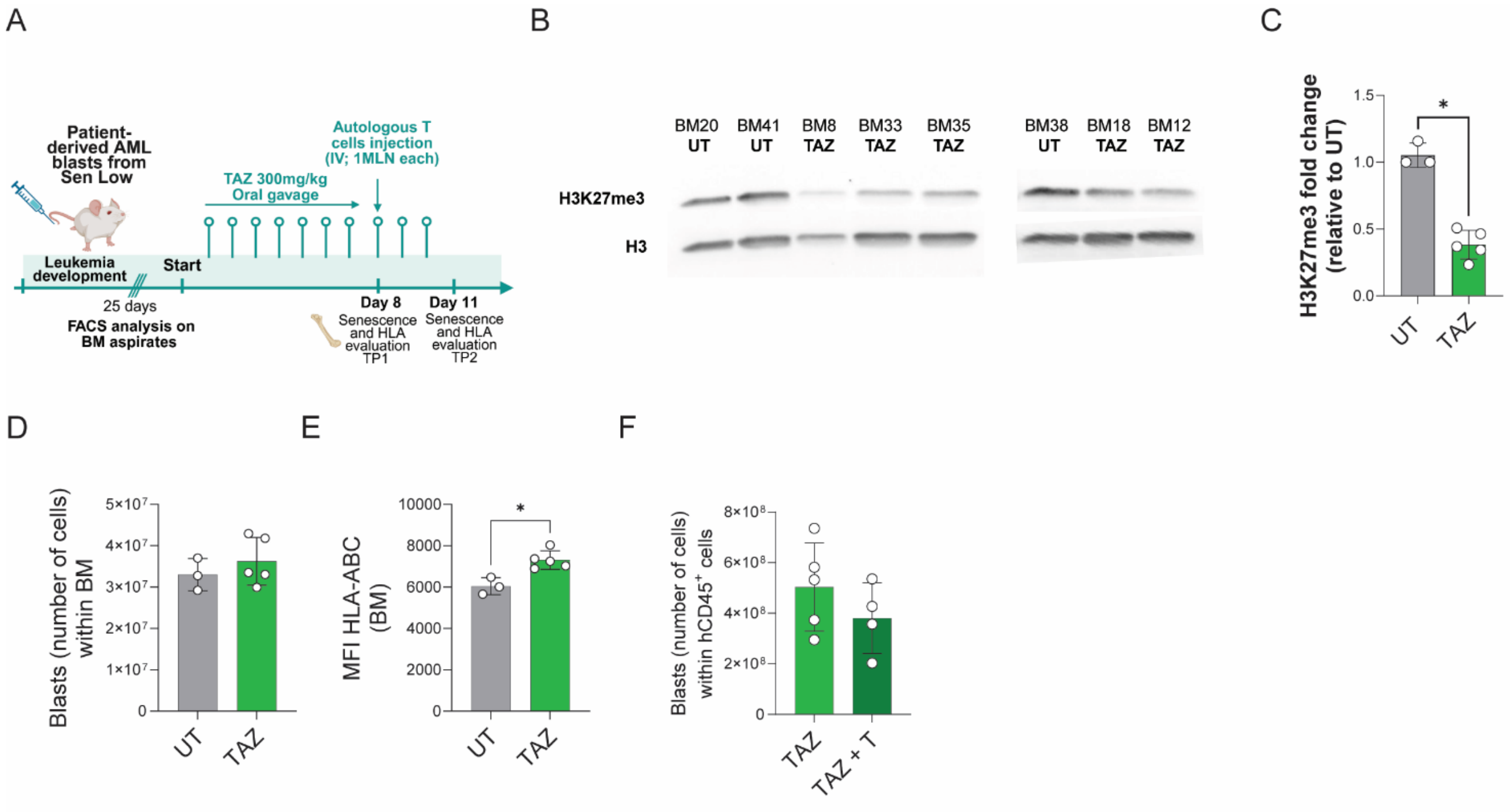

In the current experiment with Sen High blasts in the humanized NSG mice (experiment presented in figures 4F - K) it seems that in some groups about 50% of the mice were excluded from the analysis whereas I could not find an explanation to why and as such this undermines the conclusions. Better explanation and evaluation of the possible impact is warranted.

We apologize for the lack of clarity. We now better distinguished the two in vivo experimental endpoints (Endpoint 1, day 8, **Fig. 4F-I**; Endpoint 2, day 22, **Fig. 4J, K**) both in the figure and in the figure legend. For each timepoint, all the mice were included in the subsequent analyses, except for **Fig.4I**, where 4 mice in total were not included due to technical issues with the HLA-ABC staining.

Could the authors provide any evaluation on how persistent are TIS/SASP in patients or in vitro from the data available and discuss any possible negative implications?

While directly measuring how long TIS/SASP persists in patients would be highly informative, our study is designed around early, endpoint-based readouts and does not allow longitudinal quantification of persistence over time. We nevertheless agree that if senescent cells and chronic SASP persist, this could be detrimental, and we have expanded the Discussion to frame TIS as a time-dependent double-edged phenomenon and to outline how the transient immunogenic window identified here could be exploited to potentiate immune-based strategies to target senescent cells (including senolytic CAR-T based therapies).

The authors should provide proof that the H3K27me3 enrichment over the affected loci presented in figure 5G was depleted after TAZ treatment or at least global depletion of the mark.

We now provided direct evidence that EZH2 inhibition by Tazemetostat reduces H3K27me3 both globally and at immune-related loci highlighted. To this end, we set up and performed H3K27me3 Cut&Tag on primary AML blasts untreated (UT) or Tazemetostat-treated (TAZ). The libraries showed robust sequencing depth (>40 million paired-end reads per sample; values reported in text), good alignment metrics (**Panel A**), and the expected nucleosomal ladder insert-size profile (**Panel B**), supporting data quality. After peak calling and generation of a merged peak catalog, we observed that the vast majority of H3K27me3 regions are shared between UT and TAZ conditions (**Panel C**).

Importantly, quantitative differential analysis revealed a global loss of H3K27me3 upon TAZ (**Panel D**), consistent with global depletion of this histone repressive mark.
We then asked whether this loss occurs at the loci central to our mechanistic model. Focusing on senescence-associated loci corresponding to the core enriched gene set used in **Figure 6B**, we detected a consistent shift toward reduced H3K27me3 signal after TAZ (**Panel E**). We similarly observed H3K27me3 loss at interferon-related loci (**Panel F**), with representative genome tracks illustrating reduced signal at selected IFN genes (**Panel G**). Finally, we detect broad loss of H3K27me3 across multiple HLA genes (**Panel H**). Together, these analyses provide direct evidence that H3K27me3 levels over the senescence, IFN and HLA loci are reduced following TAZ treatment, and we added data from **Panel F-H** in new **Fig. 6D, E and Supplementary 6B**.
In addition to chromatin profiling, we validated global H3K27me3 depletion at the protein level. Western blot analysis of several Sen Low primary samples treated *ex vivo* with TAZ showed reduced H3K27me3 levels compared to untreated controls (**Panel I**). Consistently, in our *in vivo* Sen Low PDX setting,TAZ treatment decreased H3K27me3 levels in leukemic cells *in vivo* (**Fig. 6I**, **Supplementary Fig. 6D**).

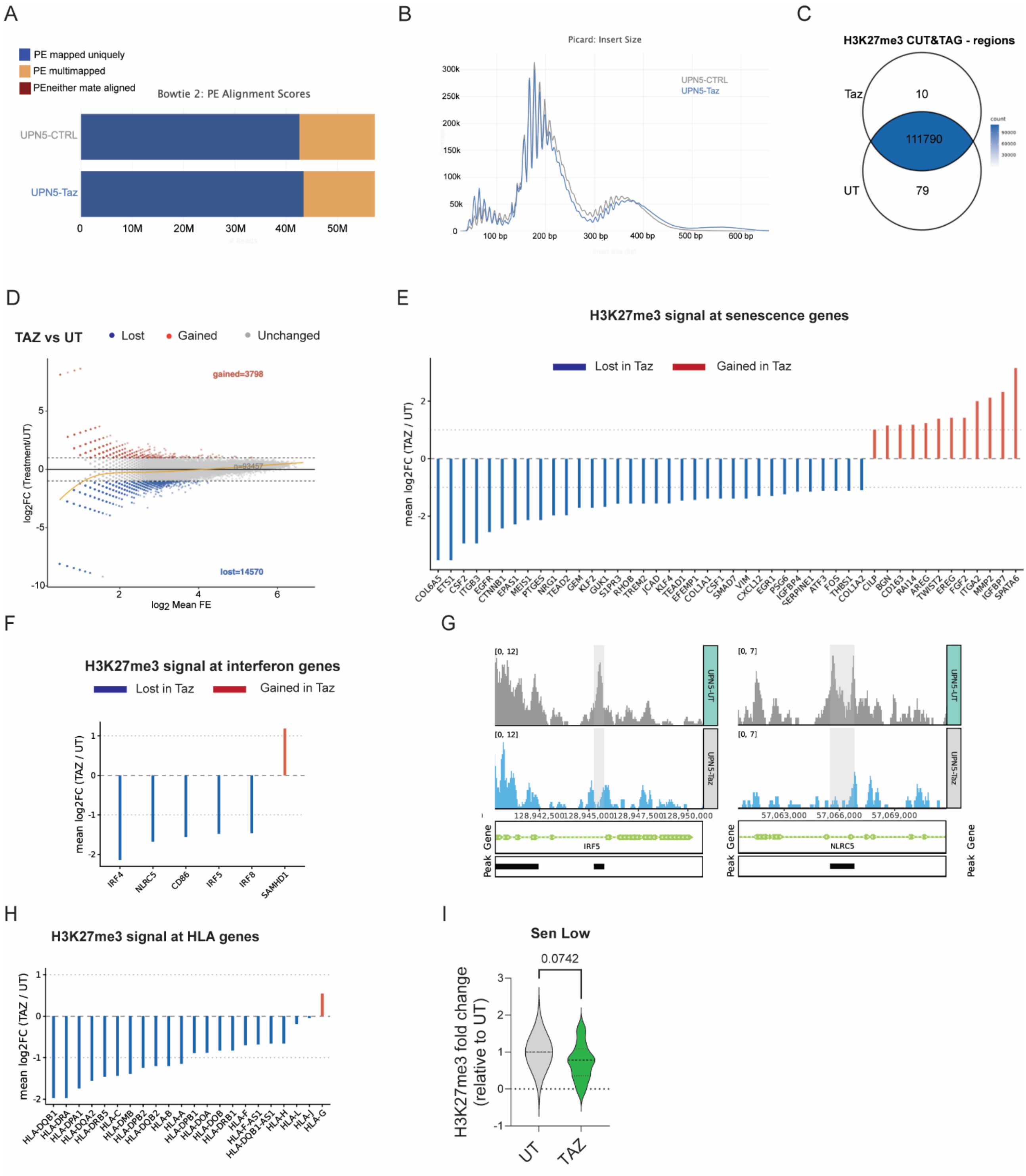

Minor comments:

a. SAHF is senescence-associated heterochromatin foci, on line 165 "foci" is missing.

b. On line 286, tazemetostat whereas on line 989 is Tazemetostat, please adhere to one and see reference article.

c. Spell out NES as Normalized Enrichment Score the first time it occurs.

We have corrected all points accordingly: (a) added the missing word "foci" in the SAHF definition (b) standardized the compound name to a single format throughout the manuscript (Tazemetostat, TAZ) and (c) spelled out NES as Normalized Enrichment Score at its first occurrence.
